# Adiposity and cancer at major anatomical sites: umbrella review of the literature

**DOI:** 10.1136/bmj.j477

**Published:** 2017-02-28

**Authors:** Maria Kyrgiou, Ilkka Kalliala, Georgios Markozannes, Marc J Gunter, Evangelos Paraskevaidis, Hani Gabra, Pierre Martin-Hirsch, Konstantinos K Tsilidis

**Affiliations:** 1 Department of Surgery and Cancer, Institute of Reproductive and Developmental Biology, Faculty of Medicine, Imperial College London, London W12 0NN, UK; 2 West London Gynaecological Cancer Centre, Queen Charlotte’s and Chelsea Hospital, Hammersmith Hospital, Imperial Healthcare NHS Trust, London, UK; 3 Department of Hygiene and Epidemiology, University of Ioannina School of Medicine, Ioannina, Greece; 4 Section of Nutrition and Metabolism, International Agency for Research on Cancer, Lyon, France; 5 Department of Obstetrics and Gynaecology, University of Ioannina, Ioannina, Greece; 6 Department of Gynaecologic Oncology, Lancashire Teaching Hospitals, Preston, UK; 7 Department of Biophysics, University of Lancaster, Lancaster, UK; 8 Department of Epidemiology and Biostatistics, School of Public Health, Imperial College London, London UK

## Abstract

**Objective** To evaluate the strength and validity of the evidence for the association between adiposity and risk of developing or dying from cancer.

**Design** Umbrella review of systematic reviews and meta-analyses.

**Data sources** PubMed, Embase, Cochrane Database of Systematic Reviews, and manual screening of retrieved references.

**Eligibility criteria** Systematic reviews or meta-analyses of observational studies that evaluated the association between indices of adiposity and risk of developing or dying from cancer.

**Data synthesis** Primary analysis focused on cohort studies exploring associations for continuous measures of adiposity. The evidence was graded into strong, highly suggestive, suggestive, or weak after applying criteria that included the statistical significance of the random effects summary estimate and of the largest study in a meta-analysis, the number of cancer cases, heterogeneity between studies, 95% prediction intervals, small study effects, excess significance bias, and sensitivity analysis with credibility ceilings.

**Results** 204 meta-analyses investigated associations between seven indices of adiposity and developing or dying from 36 primary cancers and their subtypes. Of the 95 meta-analyses that included cohort studies and used a continuous scale to measure adiposity, only 12 (13%) associations for nine cancers were supported by strong evidence. An increase in body mass index was associated with a higher risk of developing oesophageal adenocarcinoma; colon and rectal cancer in men; biliary tract system and pancreatic cancer; endometrial cancer in premenopausal women; kidney cancer; and multiple myeloma. Weight gain and waist to hip circumference ratio were associated with higher risks of postmenopausal breast cancer in women who have never used hormone replacement therapy and endometrial cancer, respectively. The increase in the risk of developing cancer for every 5 kg/m^2^ increase in body mass index ranged from 9% (relative risk 1.09, 95% confidence interval 1.06 to 1.13) for rectal cancer among men to 56% (1.56, 1.34 to 1.81) for biliary tract system cancer. The risk of postmenopausal breast cancer among women who have never used HRT increased by 11% for each 5 kg of weight gain in adulthood (1.11, 1.09 to 1.13), and the risk of endometrial cancer increased by 21% for each 0.1 increase in waist to hip ratio (1.21, 1.13 to 1.29). Five additional associations were supported by strong evidence when categorical measures of adiposity were included: weight gain with colorectal cancer; body mass index with gallbladder, gastric cardia, and ovarian cancer; and multiple myeloma mortality.

**Conclusions** Although the association of adiposity with cancer risk has been extensively studied, associations for only 11 cancers (oesophageal adenocarcinoma, multiple myeloma, and cancers of the gastric cardia, colon, rectum, biliary tract system, pancreas, breast, endometrium, ovary, and kidney) were supported by strong evidence. Other associations could be genuine, but substantial uncertainty remains. Obesity is becoming one of the biggest problems in public health; evidence on the strength of the associated risks may allow finer selection of those at higher risk of cancer, who could be targeted for personalised prevention strategies.

## Introduction

Cancer is a leading cause of death worldwide, with an estimated 12.7 million new cases and 7.6 million deaths from cancer annually.^1^ Excess body weight is associated with an increased risk of developing and dying from many diseases, including cancer, type 2 diabetes, and cardiovascular disease.^2^ Obesity has become a major public health challenge^3^; its prevalence worldwide has more than doubled among women and tripled among men in the past four decades.^4^ The number of overweight and obese people has risen from approximately 857 million in 1980 to 2.1 billion in 2013.^5^

Several meta-analyses support the link between obesity and cancer, but substantial heterogeneity exists between studies.^6^ The reported associations may be causal, but they may also be flawed, as inherent study biases such as residual confounding and selective reporting of positive results may exaggerate the effect of obesity on cancer.^7^ ^8^ ^9^ ^10^ A recent umbrella review found that, despite strong claims of a statistically significant association between type 2 diabetes and several cancers, only a fraction (14%) of the 27 studied associations were supported by robust evidence, without any potential bias.^11^

To summarise and evaluate the existing evidence and appraise its quality, we performed an umbrella review of systematic reviews and meta-analyses that investigated the association between adiposity indices and risk of developing or dying from cancer. Umbrella reviews systematically appraise the evidence on an entire topic across many meta-analyses of multiple putative risk factors on multiple outcomes.^11^ ^12^ ^13^ ^14^ ^15^ ^16^ ^17^ ^18^

## Methods

### Literature search

We searched PubMed, Embase, and the Cochrane Database of Systematic Reviews from inception to May 2015 for systematic reviews or meta-analyses that investigated the association between adiposity indices and risk of developing or dying from any cancer using a predefined search algorithm (see the supplementary file for details of the algorithm). Adiposity indices were body mass index, waist circumference, hip circumference, waist to hip ratio, weight, weight gain, and weight loss from bariatric surgery. We hand searched the references of the retrieved systematic reviews and meta-analyses and the proceedings of relevant conferences for articles missed by the electronic search and unpublished data.

### Inclusion and exclusion criteria

We included systematic reviews and meta-analyses of observational epidemiological studies in humans that assessed continuous or categorical measures of adiposity. We excluded studies in which physical activity, height, or prenatal factors (such as birth weight or maternal obesity during pregnancy) were the exposures of interest and those in which cancer incidence or mortality were not the outcomes of interest. We excluded meta-analyses of prognostic studies that investigated cancer survival or other outcomes among patients with cancer and meta-analyses that did not include comprehensive data from individual studies—such as relative risks, 95% confidence intervals, number of cases and controls, or total population. When we found more than one meta-analysis on the same association between exposure and outcome, we included the one with the largest number of primary studies to avoid duplication. We subsequently examined whether the summary estimates showed concordance between the included and duplicate meta-analyses.

### Data extraction

We extracted the name of the first author, year of publication, outcome, and exposure from each eligible systematic review or meta-analysis. From each individual study in a meta-analysis, we extracted the first author, year of publication, epidemiological design, number of cases and controls in case-control studies or total population in cohort studies, maximally adjusted relative risk (odds ratio in case-control studies and hazard ratio or standardised incidence or mortality ratio in cohort studies) and 95% confidence intervals. Two investigators (IK, MK) independently searched the literature, assessed the eligibility of the retrieved papers, and extracted the data. Disagreements were resolved by discussion with a third investigator (KT).

### Data analysis

*Estimation of summary effect*—For each exposure and outcome pair, we calculated the summary effect and the 95% confidence interval using fixed and random effects methods.^19^

*Assessment of heterogeneity*—The heterogeneity between studies was assessed with Cochran’s Q test^20^ and the I^2^ statistic.^21^ Substantial inconsistency could reflect either genuine heterogeneity between studies or bias. To assess the uncertainty around heterogeneity estimates, we calculated 95% confidence intervals.^22^

*Estimation of prediction intervals*—To further account for heterogeneity between studies we calculated 95% prediction intervals for the summary random effect estimates, which represent the range in which the effect estimates of future studies will lie.^23^

*Assessment of small study effects*—We examined whether smaller studies gave higher risk estimates than larger studies, which is an indication of publication bias, true heterogeneity, or chance.^11^ ^24^ Indication of small study effects was based on the Egger’s regression asymmetry test (P≤0.10) and whether the random effects summary estimate was larger than the point estimate of the largest study in the meta-analysis.

*Evaluation of excess significance*—We assessed excess significance bias by evaluating whether the observed number of studies with nominally statistically significant results (“positive” studies, P<0.05) in the published literature was different from the expected number of studies with statistically significant results.^25^ The expected number of statistically significant studies in each meta-analysis was calculated from the sum of the statistical power estimates for each component study using an algorithm from a non-central *t* distribution.^26^ ^27^ The power estimates of each component study depend on the plausible effect size for the tested association, which was assumed to be the effect of the largest study (that is, the smallest standard error) in each meta-analysis.^28^ Sensitivity analyses were conducted using the summary fixed and random effects estimates as alternative plausible effect sizes. Excess significance for individual meta-analyses was determined at P≤0.10.^25^

*Credibility ceilings*—We used credibility ceilings, a sensitivity analysis tool, to account for potential methodological limitations of observational studies that might lead to spurious precision of combined effect estimates.^29^ The main assumption of this method is that every observational study has a probability *c* (credibility ceiling) that the true effect size is in a different direction from the one suggested by the point estimate. The pooled effect size and the heterogeneity between studies were re-estimated using a wide range of credibility ceiling values.^29^ ^30^

### Grading the evidence

The associations between measures of adiposity and cancer were categorised into strong, highly suggestive, suggestive, or weak depending on the strength and validity of the evidence.^13^ ^15^ ^18^ A strong association was claimed when the P value of the random effects meta-analysis was smaller than 10^−6^, a threshold that might substantially reduce false positive findings^31^ ^32^ ^33^ if the meta-analysis has more than 1000 cancer cases, the largest study in the meta-analysis is nominally statistically significant (P<0.05), the I^2^ statistic of heterogeneity between studies is smaller than 50%, the 95% prediction intervals excludes the null value, small study effects or excess significance bias are not indicated, and the association survives a credibility ceiling of at least 10%.

The association was considered highly suggestive if P<10^−6^, the meta-analysis had more than 1000 cases, and the largest study in the meta-analysis was nominally statistically significant. The criteria for suggestive were: random effects P<10^−3^ and the meta-analysis had more than 1000 cases. All other associations with a nominally statistically significant P value for the random effects meta-analysis were deemed to provide weak evidence.

The primary analysis in this umbrella review focused on cohort studies using continuous measures of adiposity, which was considered a more valid and standardised way of presenting and synthesising individual study estimates than using categorical measures. Sensitivity analyses were conducted using case-control studies and categorical measures. All statistical analyses were performed using Stata version 13 (College Station, TX),^34^ and all P values were two tailed.

### Patient involvement

No patients were involved in setting the research question or outcome measures, nor were they involved in developing plans for design or implementation of the study. No patients were asked to advise on interpretation or writing up on results. The results will be disseminated to the general public through public presentations and the authors’ involvement in different charities.

## Results

### Characteristics of the meta-analyses

Overall, 49 eligible papers were identified that included a total of 204 meta-analyses. These meta-analyses summarised 2179 individual study estimates from 507 unique cohort or case-control studies (fig 1).^35^ ^36^ ^37^ ^38^ ^39^ ^40^ ^41^ ^42^ ^43^ ^44^ ^45^ ^46^ ^47^ ^48^ ^49^ ^50^ ^51^ ^52^ ^53^ ^54^ ^55^ ^56^ ^57^ ^58^ ^59^ ^60^ ^61^ ^62^ ^63^ ^64^ ^65^ ^66^ ^67^ ^68^ ^69^ ^70^ ^71^ ^72^ ^73^ ^74^ ^75^ ^76^ ^77^ ^78^ ^79^ ^80^ ^81^ ^82^ ^83^ Of the 507 unique studies, 371 (73.2%) had a cohort design, 134 (26.4%) were case-control studies, and two (0.4%) were cross sectional studies. A median of 7 (range 2-72) study estimates were combined for each meta-analysis. The median number of cases and population or controls in each meta-analysis was 5645 and 1 766 389, respectively. A total of 177 meta-analyses had at least 1000 cancer cases.

**Figure f1:**
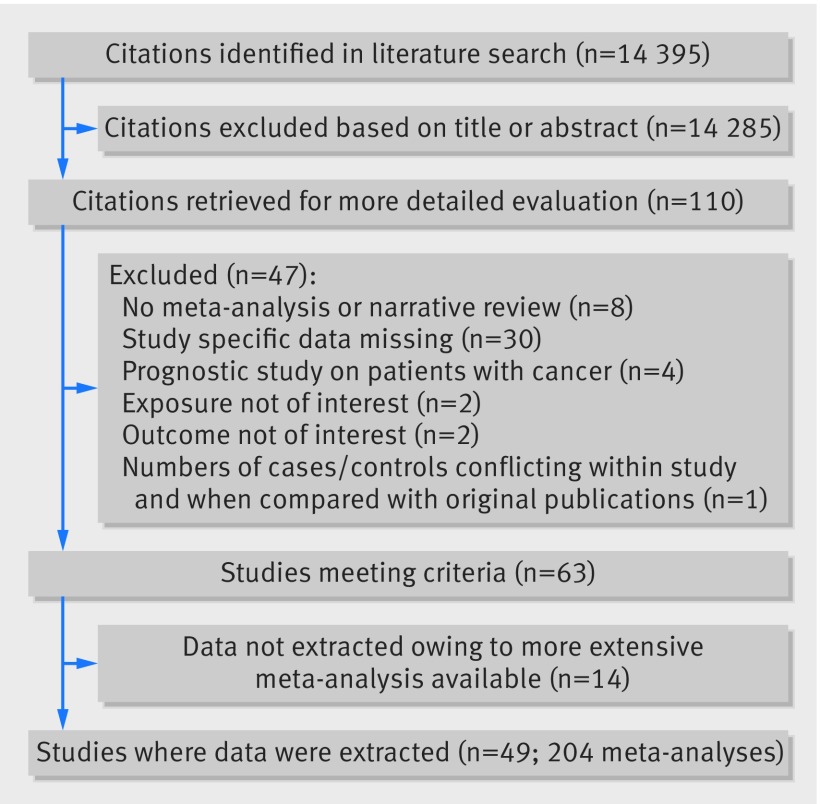
**Fig 1** Study flowchart

The 204 meta-analyses included associations between seven indices of adiposity (body mass index, waist circumference, hip circumference, waist to hip ratio, weight, weight gain, and weight loss through bariatric surgery) and the incidence (n=196) or mortality (n=8) from cancer at 36 anatomical sites and by subtypes. A total of 194 meta-analyses included cohort studies (see supplementary tables 1 and 2). Of these, 95 used a continuous scale to measure adiposity (for example, body mass index in adults or at around age 20 per 5 kg/m^2^, waist circumference per 10 cm, hip circumference per 10 cm, waist to hip ratio per 0.1, weight per 5 kg, and weight gain per 1 kg or 5 kg). These 95 meta-analyses studied 28 different cancer sites or subtypes and included 818 individual study estimates, with body mass index in adults being investigated most frequently (n=57 meta-analyses).

We identified more than one published meta-analysis with the same exposure and outcome pair for 11 cancers. All duplicate meta-analyses showed agreement on the direction, magnitude, and statistical significance of the summary associations (see supplementary table 3).

### Summary effect size

When we used a threshold of P<0.05, the summary fixed effects estimates were significant in 76 of the 95 meta-analyses (80%) and the summary random effects were significant in 72 (76%) (see supplementary table 1 and supplementary figures 1-28). At a stricter threshold of P<0.001, 66 (69%) and 59 (62%) meta-analyses produced significant summary results using the fixed and random effects models, respectively. At P<10^−6^, 54 (57%) and 35 (37%) meta-analyses were significant, respectively.

Of those 35 meta-analyses, 31 found an increased risk of cancer with higher adiposity for oesophageal adenocarcinoma, multiple myeloma, and cancers of the colon, rectum, liver, biliary tract system (cancers of gallbladder, extrahepatic bile duct, and ampulla of Vater), pancreas, postmenopausal breast, endometrium, and kidney. Four of the 35 meta-analyses indicated an inverse association for oesophageal squamous cell carcinoma and lung cancer. The magnitude of the observed summary random effect estimates ranged from 0.57 to 1.90; 61% of the estimates lay between 0.80 and 1.20 (fig 2). In meta-analyses with small variances a trend of summary effects towards 1.00 was observed. The largest study of each meta-analysis showed a nominally statistically significant association in 65 meta-analyses (68%), and the relative risks of the largest studies were more conservative (closer to null) than the summary random effects in 53 (56%) meta-analyses.

**Figure f2:**
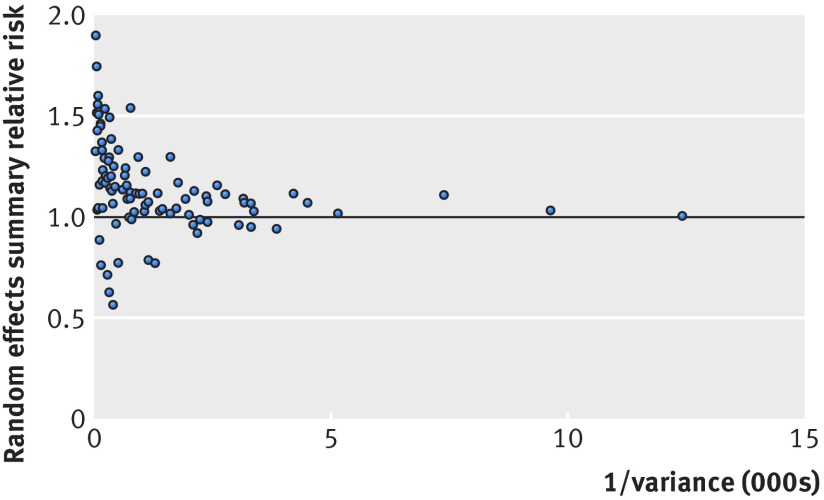
**Fig 2** Association of meta-analysis summary effect sizes with inverse of the variance

The summary estimates were similar between men and women for eight of 11 cancers (oesophageal adenocarcinoma, oesophageal squamous cell carcinoma, multiple myeloma, leukaemia, and gastric, lung, kidney, and thyroid cancers). However, the incidence of colon and rectal cancer was higher per 5 kg/m^2^ increase of body mass index in men (relative risk 1.30, 95% confidence interval 1.25 to 1.35) and 1.09 (1.06 to 1.13), respectively) than in women (1.12 (1.06 to 1.17) and 1.02 (0.99 to 1.05), respectively). This was also true for other adiposity indices. Moreover, body mass index was associated with an increased risk of melanoma in men (relative risk per 5 kg/m^2^ 1.17, 1.05 to 1.30) but not in women (0.96, 0.93 to 1.00).

### Heterogeneity between studies

Τhe Q test was significant at P≤0.10 in 42 of 95 meta-analyses (44%) (see supplementary table 2). Twenty meta-analyses (21%) showed considerable heterogeneity (I^2^=50-75%) and 15 meta-analyses (16%) showed substantial heterogeneity (I^2^ >75%) for some adiposity indices and several cancers (oesophageal squamous cell carcinoma; colon, liver, lung, endometrial, prostate, and thyroid cancers; melanoma; and leukaemia). We further assessed the uncertainty of the summary effects by calculating 95% prediction intervals; the null value was excluded in only 27 associations.

### Small study effects

Thirteen meta-analyses had evidence indicating small study effects, but only seven included 10 or more studies and therefore had enough statistical power for the Egger’s test to adequately identify presence of small study effects (colon, liver, lung, ovarian, and advanced prostate cancer; see supplementary table 2). Supplementary figures 1 to 28 show the funnel plots for each cancer site.

### Excess significance

Nineteen meta-analyses had evidence of excess significance bias for some adiposity indices using the largest study estimate as the plausible effect size (colon, rectal, liver, pancreatic, endometrial, ovarian, prostate, advanced prostate, and kidney cancer; supplementary table 2). Using summary fixed or random effects estimates as plausible effect sizes gave similar results.

### Credibility ceilings

A credibility ceiling of 0% corresponds to the random effects model calculation. Of the 95 meta-analyses, 72 (76%) retained nominal statistical significance (P<0.05) with a credibility ceiling of 5%. Fifty (53%), 33 (35%), and 19 (20%) meta-analyses remained statistically significant with ceilings of 10%, 15%, and 20%, respectively. Heterogeneity between studies gradually decreased with increasing ceilings. With a ceiling of 10%, no meta-analyses of cohort studies with continuous exposure gave an I^2^ estimate larger than 50% (supplementary table 4).

### Grading the evidence

We explored whether the reported associations between adiposity and developing or dying from cancer were supported by strong, highly suggestive, suggestive, or weak evidence (table 1). We found that 77% of the 95 meta-analyses presented at least weak evidence (P<0.05 for the summary random effects). Supplementary table 5 shows these associations in cohort studies using continuous measures of adiposity (also see supplementary table 6 for associations using categorical measures of adiposity in cohort studies and supplementary table 7 for all associations regardless of study type or exposure metric).

**Table 1 tbl1:** Summary of evidence grading for meta-analyses associating continuous measures of obesity and risk of cancer—cohort studies only. Risk refers to cancer incidence unless otherwise stated

**Evidence**	**Criteria used**	**Decreased risk**	**Increased risk**
Strong (n=12)	P<10^−6^*; >1000 cases; P<0.05 of largest study in meta-analysis; I^2^ <50%; no small study effect†; prediction interval excludes null value; no excess significance bias‡; survive 10% credibility ceiling	None	Oesophageal adenocarincoma (BMI); colon cancer, men (BMI); rectal cancer, men (BMI); biliary tract system cancer§ (BMI); pancreatic cancer (BMI); postmenopausal breast cancer, never HRT use (WG); endometrial cancer (WHR); premenopausal endometrial cancer (BMI); kidney cancer, men and women (BMI); multiple myeloma, overall and women (BMI)
Highly suggestive (n=17)	P<10^−6^*; >1000 cases; P<0.05 of largest study in meta-analysis	Oesophageal squamous cell carcinoma, overall and women (BMI); lung cancer, overall and men (BMI)	Colon cancer (BMI and waist circumference per 10 cm); liver cancer (BMI); postmenopausal breast cancer (BMI); endometrial cancer (BMI, BMI in young adulthood, weight per 5 kg, WG); postmenopausal endometrial cancer (BMI); endometrial cancer, type I (BMI); endometrial cancer, type II (BMI); kidney cancer (BMI)
Suggestive (n=23)	P <10^−3^*; >1000 cases	Oesophageal squamous cell carcinoma, men (BMI); lung cancer, smokers (BMI); premenopausal breast cancer (BMI); localised prostate cancer (BMI)	Colon cancer, women (BMI); colon cancer, men and overall (WG); colon cancer (WHR and WC); colorectal cancer (WG per 1kg); rectal cancer (BMI); pancreatic cancer (WHR and WC); ovarian cancer (BMI and BMI in young adulthood); prostate cancer mortality (BMI); thyroid cancer, overall and women (BMI); non-Hodgkin’s lymphoma (BMI); multiple myeloma, men (BMI); leukaemia (BMI)
Weak (n=19)	P<0.05*	Lung cancer, women (BMI); melanoma, women (BMI)	Oesophageal adenocarcinoma in men and women (BMI); melanoma, men (BMI); endometrial cancer (HC per 10 cm); postmenopausal endometrial cancer, never HRT use (BMI and WG); postmenopausal endometrial cancer, ever HRT use (BMI and WG); endometrial cancer mortality (BMI); ovarian cancer (weight per 5 kg); postmenopausal ovarian cancer, never HRT use (WG); prostate cancer, advanced (BMI); prostate cancer, countries with high screening rate for prostate specific antigen (WG); thyroid cancer, men (BMI); non-Hodgkin’s lymphoma mortality (BMI); leukaemia, men and women (BMI)

BMI=body mass index (per 5 kg/m^2^); HC=hip circumference; HRT=hormone replacement therapy; WHR=waist to hip ratio (per 0.1 units); WC=waist circumference (per 10 cm); WG=weight gain (per 5 kg unless otherwise stated)

*P value of meta-analysis random effects model.

†Small study effect is based on P value from Egger’s regression asymmetry test (P>0.1) where the random effects summary estimate was larger compared to the point estimate of the largest study in a meta-analysis.

‡Based on P>0.1 of excess significance test using largest study (smallest standard error) in meta-analysis as plausible effect size.

§Includes cancers of gallbladder, extrahepatic bile duct, and ampulla of Vater.

Only 13% of meta-analyses (12 of 95) were supported by strong evidence. They summarised data on body mass index (n=10), waist to hip ratio (n=1), and weight gain (n=1). An increase in body mass index was associated with a higher risk of developing oesophageal adenocarcinoma; colon and rectal cancer in men; biliary tract system and pancreatic cancer; endometrial cancer in premenopausal women; kidney cancer; and multiple myeloma. Weight gain and waist to hip circumference ratio were associated with higher risks of postmenopausal breast cancer in women who have never used hormone replacement therapy and endometrial cancer, respectively. The increase in the risk of developing cancer for every 5 kg/m^2^ increase in body mass index ranged from 9% (relative risk 1.09, 95% confidence interval 1.06 to 1.13) for colorectal cancer among men to 56% (1.56, 1.34 to 1.81) for biliary tract system cancer. The risk of postmenopausal breast cancer among women who have never used HRT increased by 11% for each 5 kg of weight gain (1.11, 1.09 to 1.13), and the risk of endometrial cancer increased by 21% for each 0.1 increase in waist to hip ratio (1.21, 1.13 to 1.29).

Seventeen meta-analyses (18%) were supported by highly suggestive evidence, and they found positive associations for colon (with body mass index and waist circumference), liver (with body mass index), postmenopausal breast (with body mass index), total endometrial (with body mass index in adults and at around age 20, waist circumference, weight, and weight gain), postmenopausal, type I and type II endometrial (with body mass index), and kidney cancer (with body mass index). Inverse associations were found for body mass index in adults with oesophageal squamous cell carcinoma (overall and in women) and lung cancer (overall and in men). Twenty four meta-analyses (25%) were supported by suggestive evidence for an association, and 19 meta-analyses (20%) were supported by weak evidence.

The evidence was graded similarly in the 194 meta-analyses of cohort studies that assessed continuous and categorical measures of adiposity (supplementary table 6). Five additional associations were supported by strong evidence: weight gain with incidence of colorectal cancer, body mass index with incidence of gallbladder, gastric cardia, and ovarian cancers, and mortality from multiple myeloma. When both cohort and case-control studies were assessed for continuous and categorical measures of adiposity (supplementary table 7), two additional associations were supported by strong evidence: body mass index and risk of melanoma and meningioma, both of which were judged as weak associations when only cohort studies were evaluated. The strong evidence for an association between body mass index and incidence of biliary tract system cancerwas downgraded to suggestive evidence when both case-control and cohort studies were evaluated.

## Discussion

We reviewed 204 meta-analyses to evaluate the current evidence for associations between seven adiposity indices and the risk of developing or dying from 36 primary cancers and their subtypes. Twelve associations, stemming from cohort studies using continuous measures of adiposity, were supported by strong evidence, inferred by strongly statistically significant results and no suggestion of bias. These associations were primarily between body mass index and malignancies of digestive organs (oesophageal adenocarcinoma and cancers of the colorectum (in men only), biliary tract system, and pancreas), hormone related cancers (such as postmenopausal breast in women who have never used HRT), premenopausal and overall endometrial cancer, kidney cancer, and multiple myeloma. Five additional associations were supported by strong evidence when categorical measures of adiposity were used: weight gain with risk of colorectal cancer risk and body mass index with risk of gallbladder, gastric cardia, and ovarian cancer, and mortality from multiple myeloma.

The effect of obesity on the incidence and mortality of cancer is well recognised^6^ ^62^ ^84^ and was evident in our umbrella review, with approximately 77% of the included meta-analyses reporting a nominally statistically significant summary random effects estimate. Although the reported associations are plausibly accurate and potentially causal, the risk of reporting, selection, and other inherent biases may overestimate the suggested associations, as shown in other recently published umbrella reviews in cancer epidemiology.^11^ ^13^ ^27^ The extent to which the literature is affected by such biases is difficult to prove definitively. We used statistical tests and sensitivity analyses to look for evidence of bias. When lower P value thresholds (P<10^−6^) were used, the proportion of significant associations decreased to 37%. Large heterogeneity (I^2^ ≥50%) was observed in 37% of the meta-analyses. When we calculated the 95% prediction intervals, which further account for heterogeneity, we found that the null value was excluded in only about one third of the associations. Moreover, some meta-analyses had evidence of small study effects or excess significance bias. Most meta-analyses (53%) preserved their statistical significance with a 10% credibility ceiling, but only one in five preserved significance at a ceiling of 20%, indicating that many associations between adiposity and cancer remain uncertain.

### Comparison with other studies

Our grading of the evidence largely agrees with systematic analyses of the literature performed by the World Cancer Research Fund (WCRF) and the International Agency for Research on Cancer (IARC).^6^ ^84^ ^85^ The WCRF currently lists seven cancers for which the evidence supports a convincing causal relationship with obesity (oesophageal adenocarcinoma and cancers of the pancreas, colorectum, postmenopausal breast, endometrium, kidney, and liver). We also found strong evidence that obesity increases the risk of these cancers, except for liver cancer, for which the evidence was considered highly suggestive because of small study effects, excess significance bias, and substantial heterogeneity between studies. The association between obesity and five more malignancies (gallbladder, stomach cardia, ovarian, advanced prostate, and premenopausal breast cancer) was graded as probably causal by WCRF and received lower evidence grades in our main analysis using only continuous measures of adiposity. IARC found sufficient evidence to support the association between excess body fat and 13 of 24 cancer sites (oesophagus (adenocarcinoma), gastric cardia, colorectum, liver, gallbladder, pancreas, postmenopausal breast, endometrium, ovary, kidney, meningioma, thyroid, and multiple myeloma).^84^ Our results are similar to those of the IARC report for most cancers, except for gastric cardia, liver, ovarian, meningioma and thyroid cancers. These cancers received lower than strong evidence grades in our main analysis, owing to small numbers of cancer cases, very large heterogeneity between studies, or evidence of small study effects and excess significance bias. However, the associations between adiposity and risk of gastric cardia and ovarian cancer were judged to be supported with strong evidence when we evaluated categorical measures of adiposity. Similarly, evidence for an association between adiposity and risk of meningioma was considered strong when case-control studies were included in the evaluation.

Several methods exist for rating evidence, but they are inconsistent and allow some degree of arbitrariness.^86^ The WCRF and IARC used expert groups and criteria similar to Bradford Hill criteria to assess the association between obesity and risk of 17 and 24 primary cancers, respectively, whereas we assessed the robustness of the evidence using sensitivity analyses and statistical tests to evaluate 36 primary cancers and their subtypes, defined by anatomical location, histology, and receptor status. We also explored associations by potential effect modifiers (eg, sex, menopausal status, smoking status, and use of HRT). Our criteria for grading evidence should not be considered causal criteria, especially when used individually, but we think that they are useful for identifying biases when used together.^12^

Most of the associations between adiposity indices and endometrial cancer were supported by strong or highly suggestive evidence. In particular, the association between waist to hip ratio and risk of total endometrial cancer was supported by strong evidence, indicating that central obesity, which is linked to hyperinsulinaemia and type 2 diabetes, has a major role in the development of this disease. Strong evidence also supported the association between body mass index and premenopausal endometrial cancer. We found the associations between body mass index in adults or at around age 20, waist circumference, weight, and weight gain and total endometrial cancer, and between body mass index and postmenopausal type I and II endometrial cancer to be highly suggestive owing to substantial heterogeneity between studies and potential for excess significance bias. These results were in agreement with the WCRF and IARC, which did not provide separate ratings for menopausal status or histological subtype of disease.^84^ ^87^ The difference in evidence ratings between premenopausal and postmenopausal endometrial cancer is probably related to heterogeneity caused by use of HRT among postmenopausal women.^88^ We found that the associations between body mass index or weight gain and postmenopausal endometrial cancer were statistically significantly stronger in never users compared with ever users of HRT, but the relevant meta-analyses included only 2-6 studies and had fewer than 1000 cancer cases, so the evidence was deemed weak. The WCRF found that the associations between waist circumference or waist to hip ratio and total endometrial cancer were probably causal,^87^ which largely agreed with our findings.

We found strong evidence to support the association between weight gain in adulthood and postmenopausal breast cancer among women who have never used HRT, yielding an 11% higher risk per 5 kg of weight gain. We found highly suggestive evidence to support the positive association of body mass index with postmenopausal breast cancer, irrespective of HRT. Weight gain may be a better metric than body mass index for measuring the dynamic nature of adiposity during adulthood, when obesity becomes central and has more metabolic effects.^49^ Another reason for the lower grading of the evidence for body mass index was the large heterogeneity between studies,^62^ which could at least partially be explained by a potential interaction with HRT use. Some cohort studies have found a positive association between body mass index and postmenopausal breast cancer only among women who have never used HRT,^89^ ^90^ but we did not capture this potential interaction because published meta-analyses have not performed this subgroup analysis.^62^ ^78^ The evidence for an association between adiposity indices and premenopausal breast cancer was weaker and only reached suggestive evidence for an inverse association with body mass index in a meta-analysis of 20 cohort studies. These results largely agree with the WCRF and IARC findings of a convincing causal positive association between adiposity and postmenopausal breast cancer^84^ ^91^ and a probable causal inverse association for premenopausal disease.^91^ Two recent Mendelian randomisation studies found that a higher body mass index in adults was associated with a lower risk of postmenopausal breast cancer, contradicting the epidemiological evidence.^92^ ^93^ This may be due to the genetic risk score for body mass index in adults being a stronger determinant of early compared with later life body mass index; epidemiological studies have found inverse associations between body mass index in childhood and both premenopausal and postmenopausal breast cancer.^94^ ^95^ The positive association between body mass index in adults and postmenopausal breast cancer found in epidemiological studies may be driven by weight gain in adulthood. We found strong evidence to support the association between weight gain in adults and postmenopausal breast cancer, but weight gain is probably linked to environmental factors that are not captured by genetic risk factors.^96^ Moreover, some evidence exists that these associations may differ according to oestrogen and progesterone receptor status of the tumour (supplementary tables 6 and 7),^68^ ^97^ but large investigations in this area are lacking.

The association between body mass index and colon cancer was supported by strong evidence in men and suggestive evidence in women. Twenty four cohorts investigated the association in men, totalling a 30% higher risk of colon cancer for each 5 kg/m^2^ increase in body mass index. The risk was 12% higher for each 5 kg/m^2^ increase in body mass index among women in a meta-analysis of 20 cohorts that showed substantial heterogeneity and evidence of small study effects and excess significance bias. The association between body mass index and rectal cancer in men was supported by strong evidence, but with a summary relative risk considerably smaller than for colon cancer (1.09 *v* 1.30). We found no association between body mass index and rectal cancer in women. The evidence for most of the other adiposity indices was suggestive, as few studies have investigated these associations. The insulin signalling pathway is a possible mechanism underlying the association between obesity and colorectal cancer in men.^98^ The increased concentrations of circulating insulin induced by adiposity are higher among men than women,^99^ and men are more prone to abdominal fatness than women.^100^ Furthermore, endogenous and exogenous oestrogens have been associated with protective effects against colorectal cancer among women, which might explain why the association of adiposity with colorectal cancer is stronger in men than in women.^101^ ^102^ Two Mendelian randomisation studies found statistically significant positive associations between adult body mass index and colorectal cancer but did not perform analyses by sex.^92^ ^103^

We found that higher body mass index was associated with an increased risk of oesophageal adenocarcinoma, and this was supported by strong evidence. We found an inverse association for oesophageal squamous cell carcinoma, which was supported by highly suggestive evidence due to considerable heterogeneity between studies. Similar summary associations were found by the WCRF and IARC, which concluded that adiposity convincingly increased the risk of oesophageal adenocarcinoma.^84^ ^104^ A Mendelian randomisation study supports this association.^105^ Oesophageal squamous cell carcinoma has stronger associations with smoking and alcohol consumption than oesophageal adenocarcinoma,^106^ but we were unable to capture the potential residual confounding or effect modification of smoking and alcohol in the associations between adiposity and oesophageal squamous cell carcinoma because the published meta-analysis did not report these subgroup analyses.^62^

We found an inverse association between body mass index and lung cancer that was supported by highly suggestive evidence but had substantial heterogeneity between studies and evidence of small study effects. When we investigated this association by smoking status, we found an inverse association between body mass index and lung cancer among smokers, which was supported by suggestive evidence. We found a non-significant association among non-smokers. These results may be due to residual confounding, reverse causation, or effect modification by smoking, which led the WCRF and IARC to grade this evidence as inadequate.^84^ ^107^ Two recent Mendelian randomisation studies found that body mass index was statistically significantly associated with an increased risk of squamous and small cell lung cancer but had a borderline significant inverse association with lung adenocarcinoma.^92^ ^108^ One of the Mendelian randomisation studies found a statistically significant inverse association between body mass index and total lung cancer among never smokers and a null association in ever smokers,^108^ which contradicts the epidemiological evidence. However, these analyses were underpowered. Future large prospective studies should evaluate associations according to smoking status among different disease subtypes.

Furthermore, we found that the associations between body mass index and multiple myeloma and cancers of the biliary tract system, pancreas, and kidney were supported by strong evidence. The WCRF and IARC ratings were similar, except the WCRF did not study multiple myeloma.^84^ ^85^ The associations for less common malignancies are supported by limited data and show substantial heterogeneity between studies; we need prospective studies to better characterise these associations.

### Limitations of this study

Our review relied on previously published meta-analyses and the literature searches performed by the authors of those meta-analyses. Some studies may have been missed, although this is unlikely to have influenced our findings because our assessment of duplicate meta-analyses on the same exposure-outcome associations gave similar summary results. Some of the meta-analyses that used continuous measures of adiposity were published as long ago as 2008, which may mean that we didn’t include the most recent evidence. However, we found that the evidence grading for recently published meta-analyses investigating the association of categorical terms of body mass index with the same cancers was similar.

We evaluated all study specific results that were reported in the meta-analyses (for example, primary cancers, cancer subtypes, sex, menopausal status, smoking status, and HRT use), but we may have missed other subanalyses that were not reported with sufficient study specific data. Assessing the quality of the primary studies included in the meta-analyses was beyond the scope of this umbrella review. Finally, the statistical tests we used to assess bias do not prove its definitive presence or its exact source. However, our estimates are likely to be conservative, as a negative test does not exclude the potential for bias. Moreover, our evidence grading was not sensitive to the use of 95% prediction intervals, excess significance tests, or credibility ceilings, because when we consecutively removed these criteria from the quality of evidence algorithm, the resulting inference remained the same.

### Conclusion

The association between obesity and risk of developing or dying from cancer has been extensively studied. We found strong evidence to support the positive association between obesity and 11 of the 36 cancer sites and subtypes that we examined, predominantly comprising cancers of the digestive organs and hormone related malignancies in women. Substantial uncertainty remains for the other cancers. To draw firmer conclusions we need prospective studies and large consortiums with better assessment of the changing nature of body fatness and with comprehensive standardised reporting of analyses. As obesity becomes one of the greatest public health problems worldwide, evidence of the strength of the associations between obesity and cancer may allow finer selection of people at high risk, who could be selected for personalised primary and secondary prevention strategies.

**What is already known on this topic**

Cancer is a leading cause of death worldwide, and the prevalence of obesity has more than doubled over the past 40 years
Numerous meta-analyses support the link between excess body weight and increased risk of developing and dying from several types of cancer
The reported associations may be causal for some malignancies, but they may be flawed owing to inherent biases that exaggerate the effect of obesity on cancer incidence and mortality

**What this study adds**

Strong evidence supported the association between obesity and only 11 of 36 cancer sites and subtypes, predominantly comprising cancers of digestive organs and hormone related malignancies in women
Other associations could be genuine, but substantial uncertainty remains

## Web Extra

Extra material supplied by the author

## Notes

Ethical approval: Not required.

Data sharing: No additional data available.

Transparency: The lead author (MK) affirms that the manuscript is an honest, accurate, and transparent account of the study being reported; that no important aspects of the study have been omitted; and that any discrepancies from the study as planned have been explained.
