# Supplementary material for: Adiposity and cancer at major anatomical sites: umbrella review of the literature
Source: BMJ. 2017 Feb 28;356:j477. doi: 10.1136/bmj.j477 (PMC5421437; doi:10.1136/bmj.j477)

BMI per 5kg/m2:  Oeophageal adenocarcinoma inc, overall

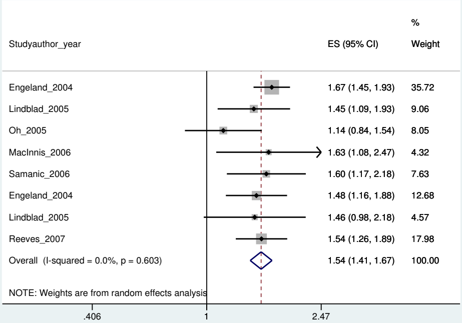


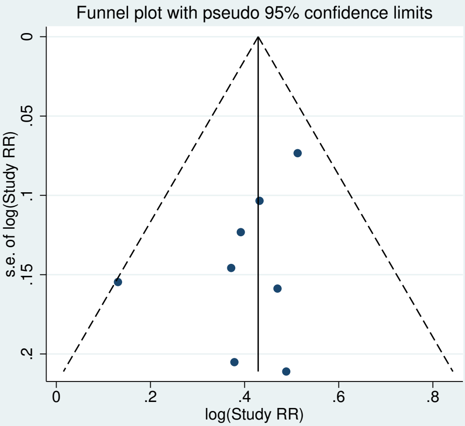


BMI per 5kg/m2: Oesophageal adenocarcinoma inc, men

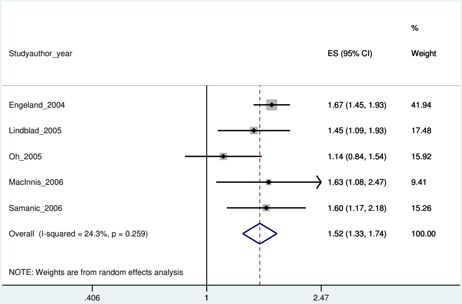


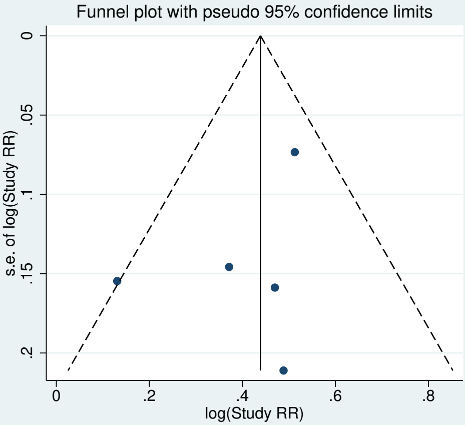


BMI per 5kg/m2: Oesophageal adenocarcinoma inc, women

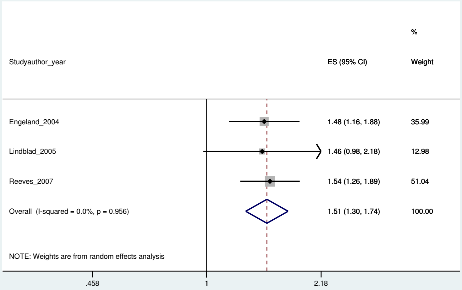


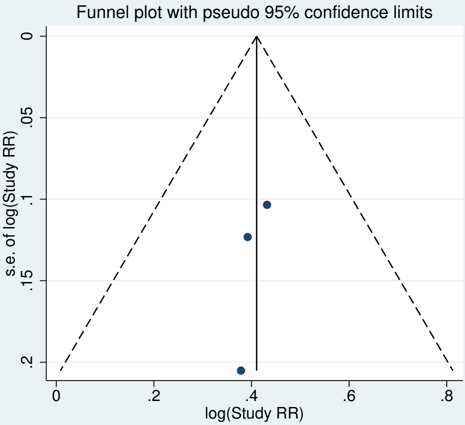

BMI per 5kg/m2: Oesophageal squamous cell carcinoma inc, overall

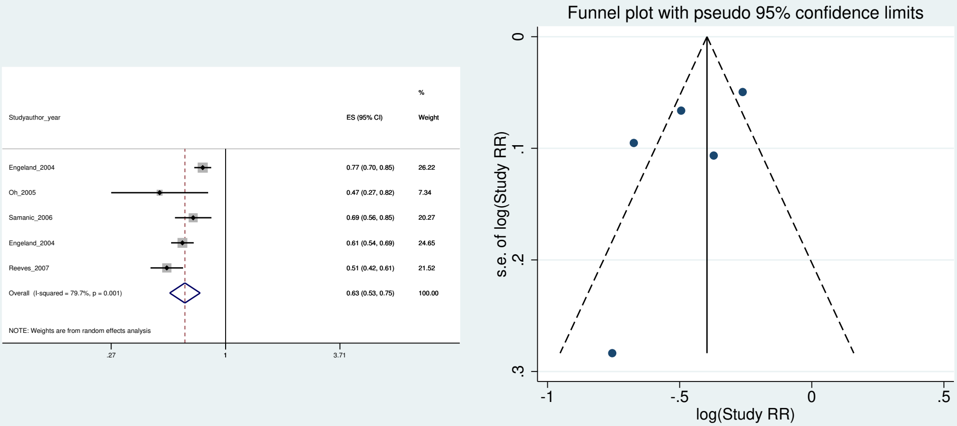


BMI per 5kg/m2: Oesophageal squamous cell carcinoma inc, men

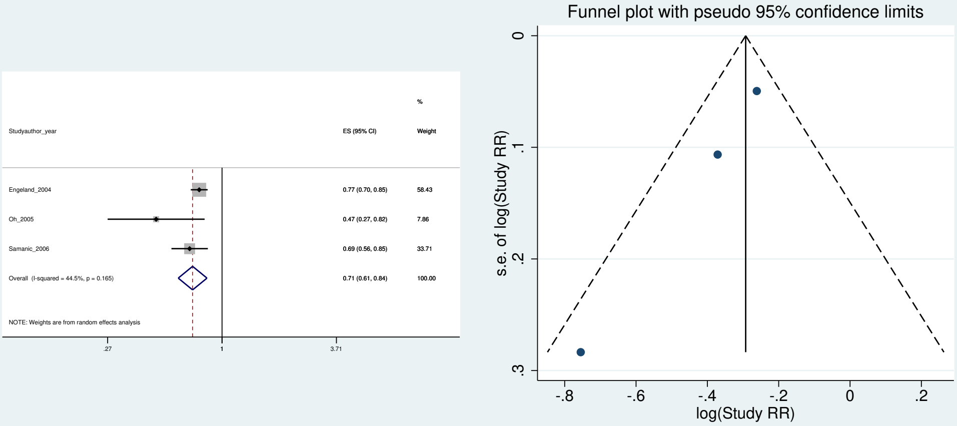


BMI per 5kg/m2: Oesophageal squamous cell carcinoma inc, women

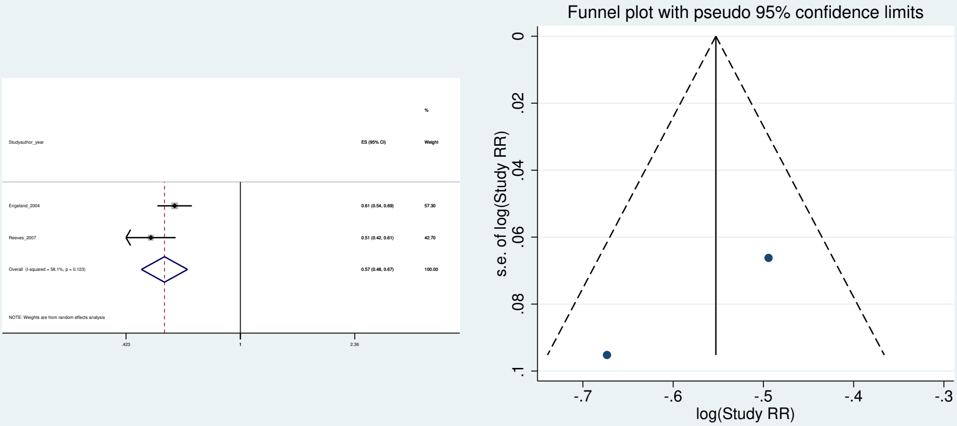

BMI per 5kg/m2: Gastric cancer inc, overall

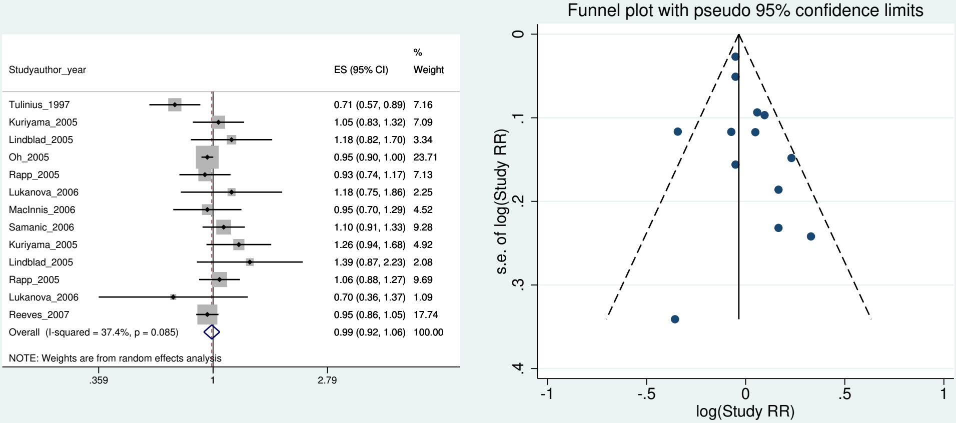


BMI per 5kg/m2: Gastric cancer inc, men

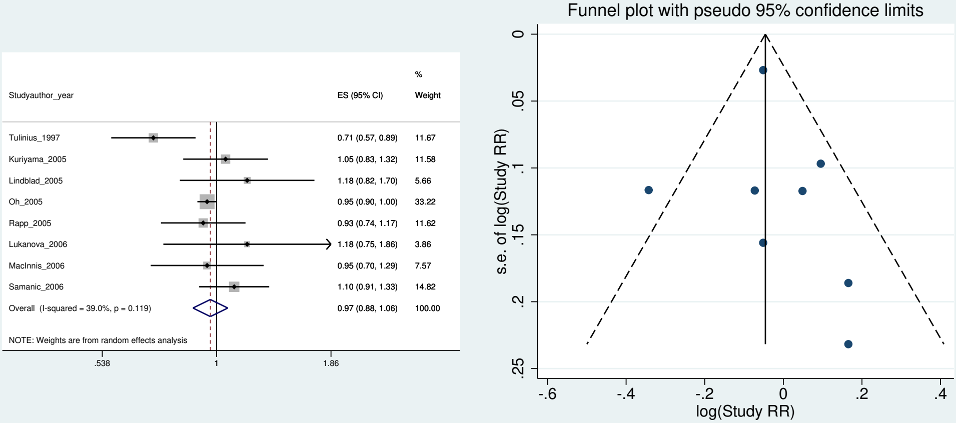


BMI per 5kg/m2: Gastric cancer inc, women

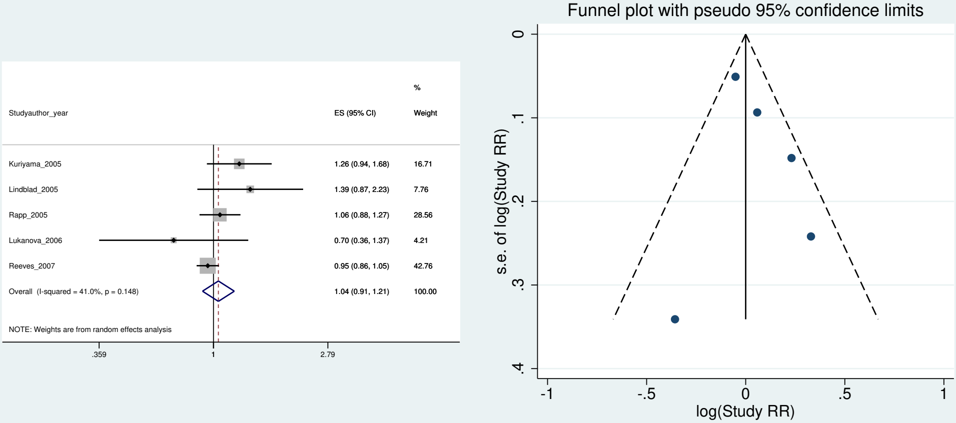

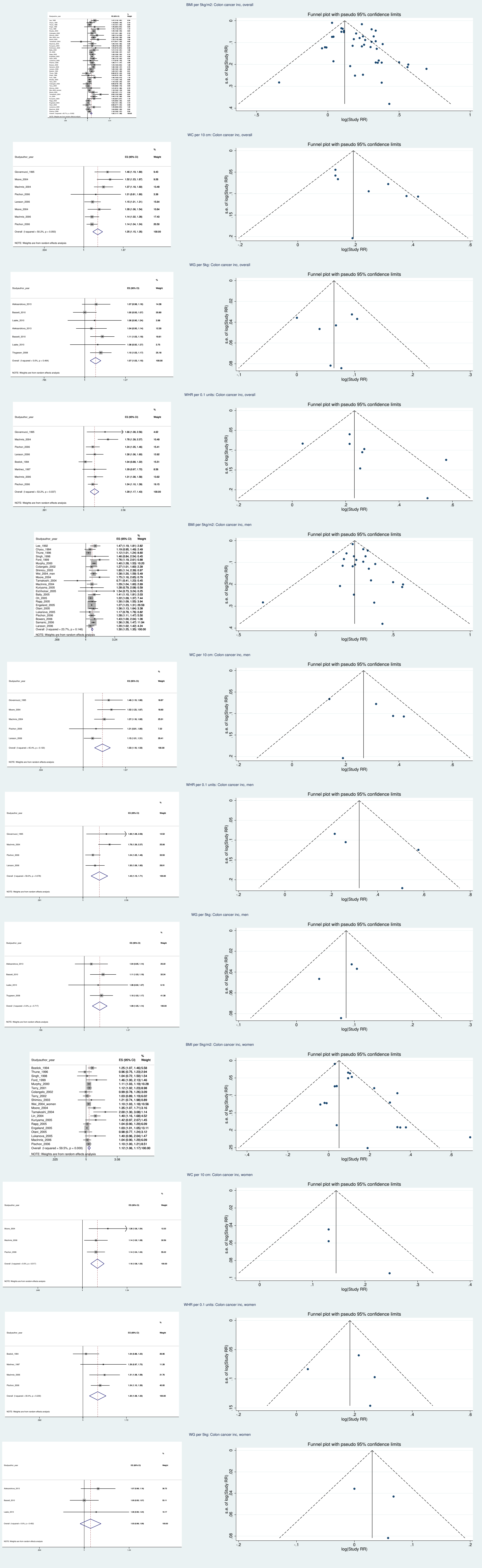
BMI per 5kg/m2: Colon cancer inc, overall
Funnel plot with pseudo 95% confidence limits
WC per 10 cm: Colon cancer inc, overall
Funnel plot with pseudo 95% confidence limits
WG per 5kg: Colon cancer inc, overall
Funnel plot with pseudo 95% confidence limits
WHR per 0.1 units: Colon cancer inc, overall
Funnel plot with pseudo 95% confidence limits
BMI per 5kg/m2: Colon cancer inc, men
Funnel plot with pseudo 95% confidence limits
WC per 10 cm: Colon cancer inc, men
Funnel plot with pseudo 95% confidence limits
WHR per 0.1 units: Colon cancer inc, men
Funnel plot with pseudo 95% confidence limits
WG per 5kg: Colon cancer inc, men
Funnel plot with pseudo 95% confidence limits
BMI per 5kg/m2: Colon cancer inc, women
Funnel plot with pseudo 95% confidence limits
WC per 10 cm: Colon cancer inc, women
Funnel plot with pseudo 95% confidence limits
WHR per 0.1 units: Colon cancer inc, women
Funnel plot with pseudo 95% confidence limits
WG per 5kg: Colon cancer inc, women
Funnel plot with pseudo 95% confidence limits

WG per 1kg/year: Colorectal cancer inc

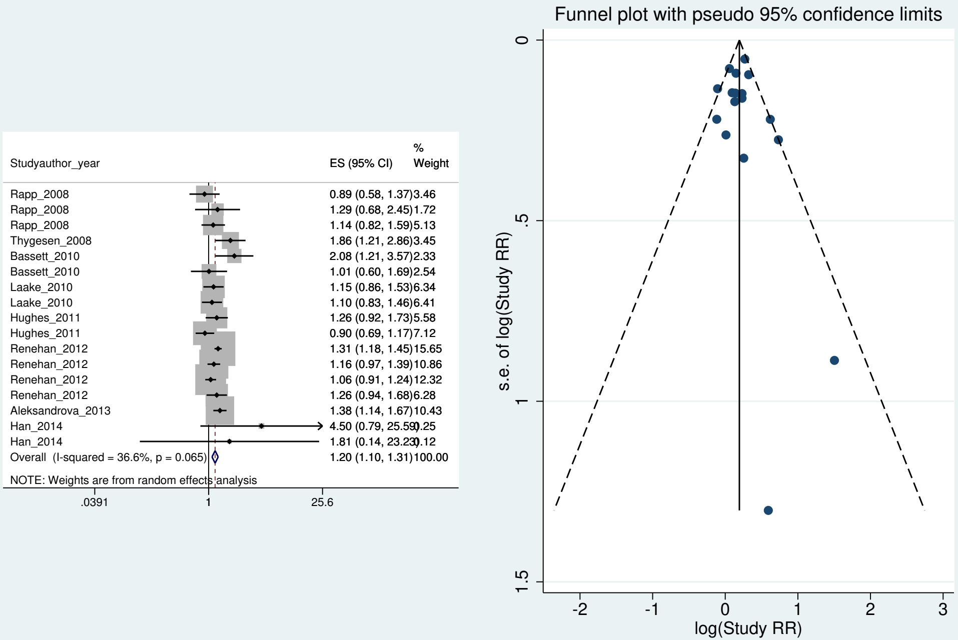

BMI per 5kg/m2: Rectal cancer inc, overall

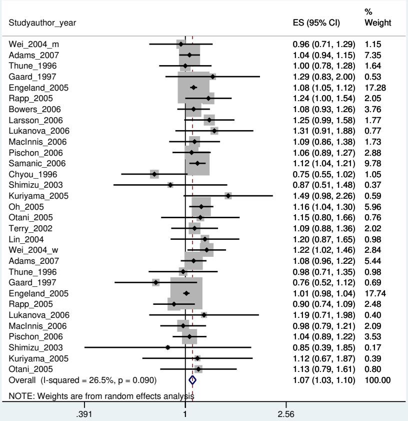


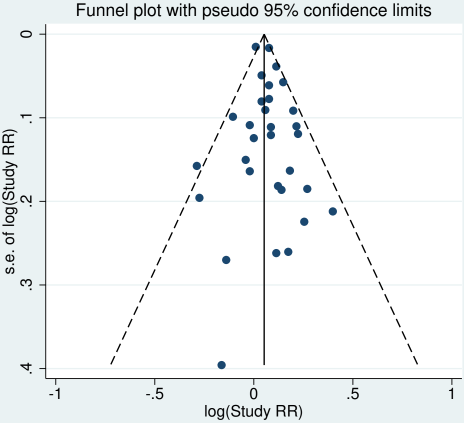


BMI per 5kg/m2: Rectal cancer inc, men

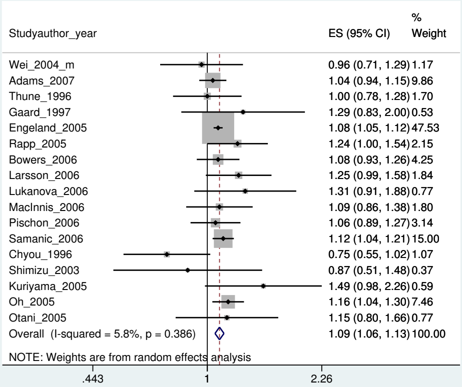


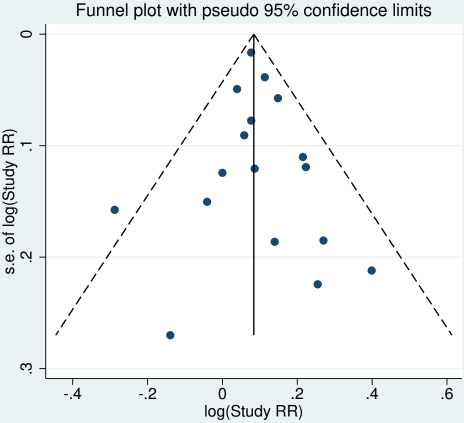


BMI per 5kg/m2: Rectal cancer inc, women

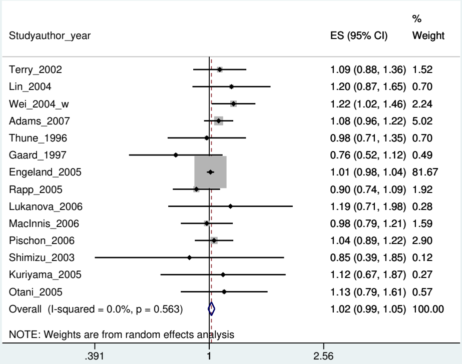


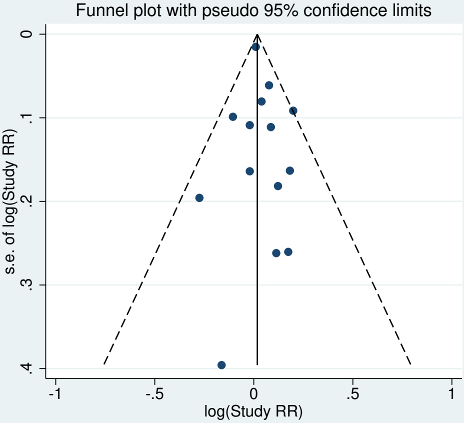

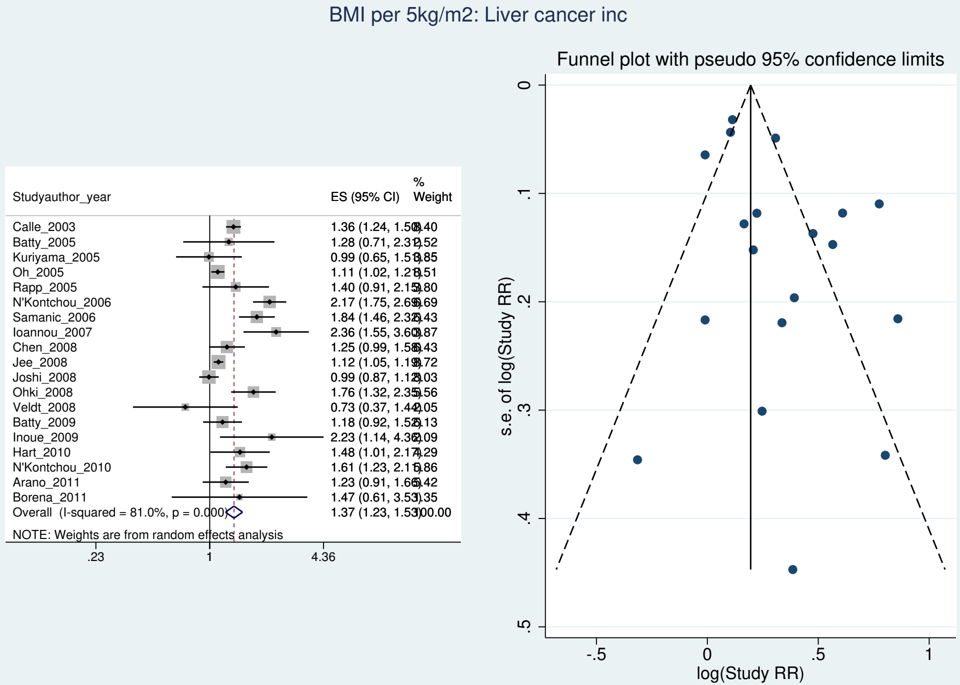
BMI per 5kg/m2: Liver cancer inc
Studyauthor_year
ES (95% CI)
% Weight
Calle_2003 1.36 (1.24, 1.50) 8.40
Batty_2005 1.28 (0.71, 2.31) 2.52
Kuriyama_2005 0.99 (0.65, 1.51) 3.85
Oh_2005 1.11 (1.02, 1.21) 8.51
Rapp_2005 1.40 (0.91, 2.15) 3.80
N'Kontchou_2006 2.17 (1.75, 2.69) 6.69
Samanic_2006 1.84 (1.46, 2.32) 6.43
Ioannou_2007 2.36 (1.55, 3.60) 3.87
Chen_2008 1.25 (0.99, 1.58) 6.43
Jee_2008 1.12 (1.05, 1.19) 8.72
Joshi_2008 0.99 (0.87, 1.12) 8.03
Ohki_2008 1.76 (1.32, 2.35) 5.56
Veldt_2008 0.73 (0.37, 1.44) 2.05
Batty_2009 1.18 (0.92, 1.52) 6.13
Inoue_2009 2.23 (1.14, 4.36) 2.09
Hart_2010 1.48 (1.01, 2.17) 4.29
N'Kontchou_2010 1.61 (1.23, 2.11) 5.86
Arano_2011 1.23 (0.91, 1.66) 5.42
Borena_2011 1.47 (0.61, 3.53) 1.35
Overall (I-squared = 81.0%, p = 0.000) 1.37 (1.23, 1.53) 100.00
NOTE: Weights are from random effects analysis
.23
1
4.36
Funnel plot with pseudo 95% confidence limits
s.e. of log(Study RR)
0
.1
.2
.3
.4
.5
-.5
0
.5
1
log(Study RR)

## BMI per 5kg/m2: Biliary tract cancer inc overall

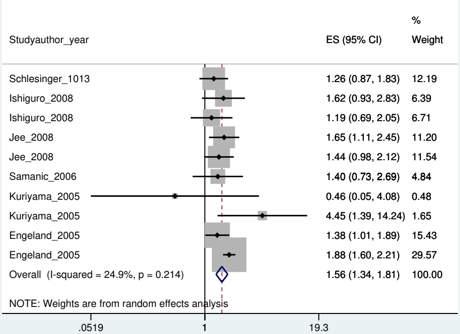


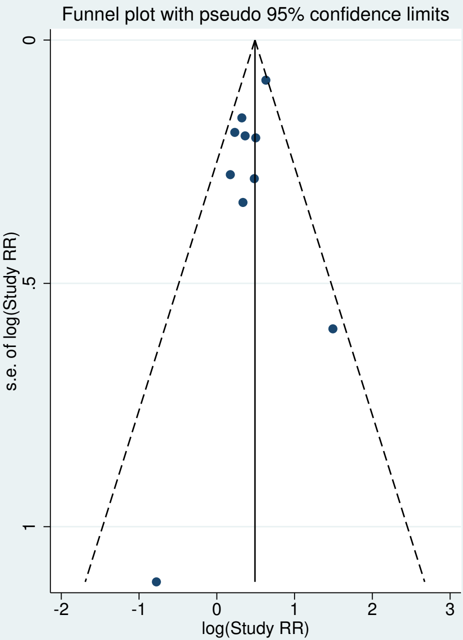

BMI per 5kg/m2: Pancreatic cancer inc

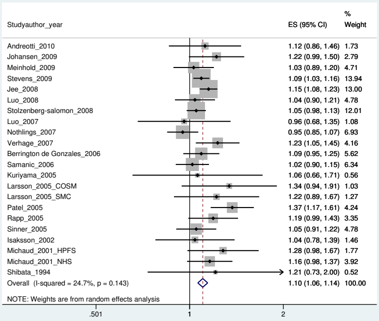


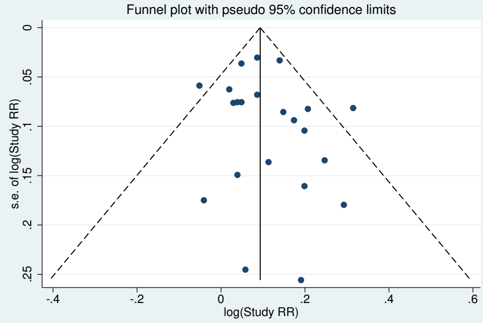


WC per 10 cm: Pancreatic cancer inc

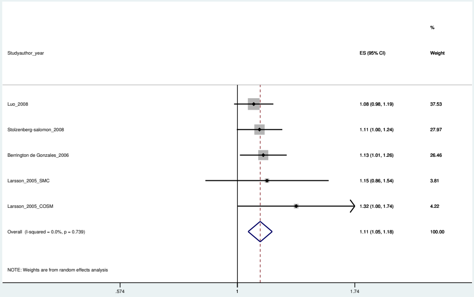


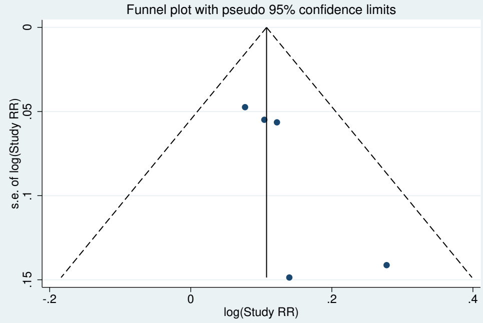


WG per 5kg: Pancreatic cancer inc

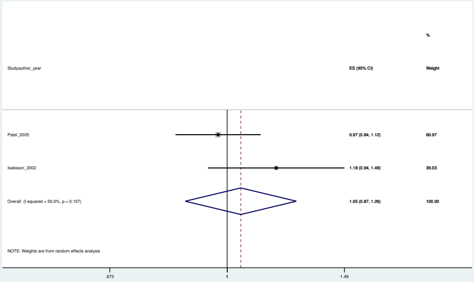


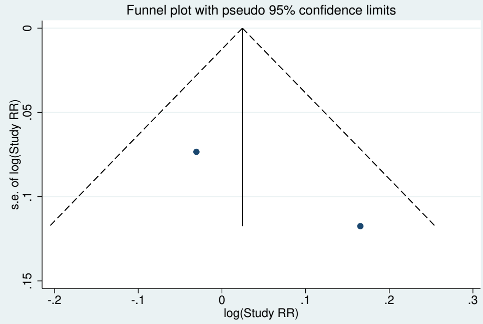


WHR per 0.1 units: Pancreatic cancer inc

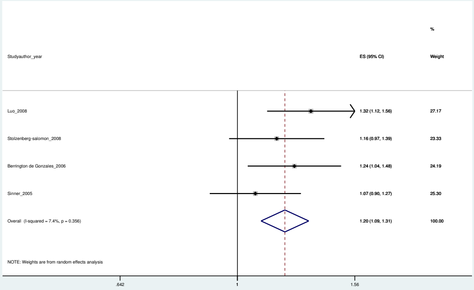


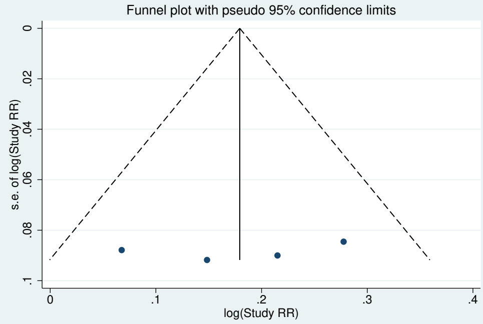


BMI per 5kg/m2: Pancreatic cancer mortality

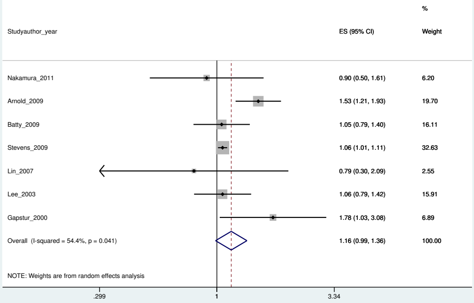


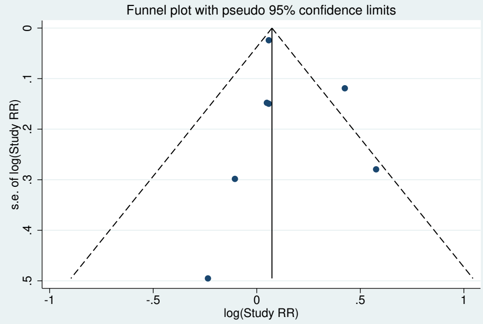

BMI per 5kg/m2: Lung cancer inc, overall

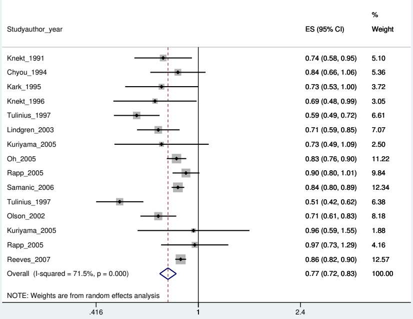


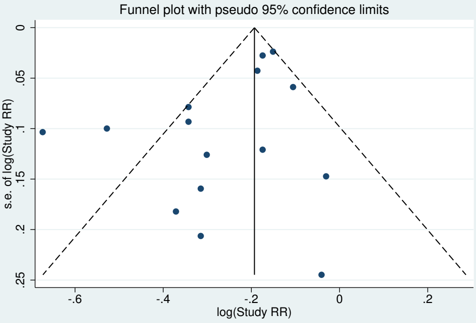


BMI per 5kg/m2: Lung cancer inc, men

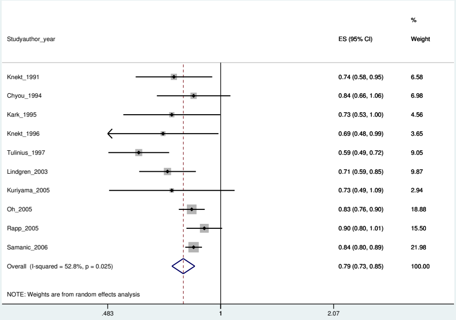


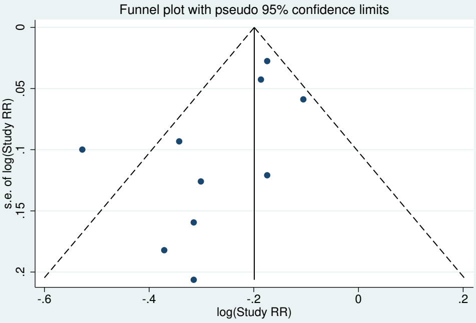


BMI per 5kg/m2: Lung cancer inc, women

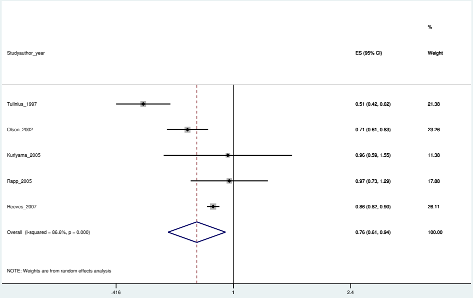


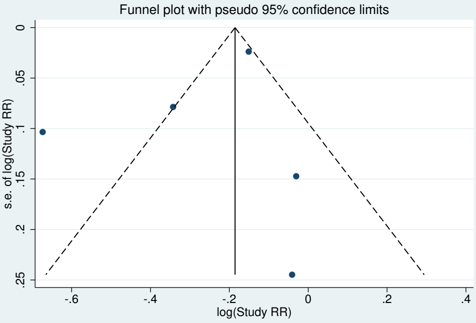


BMI per 5kg/m2: Lung cancer inc, smokers

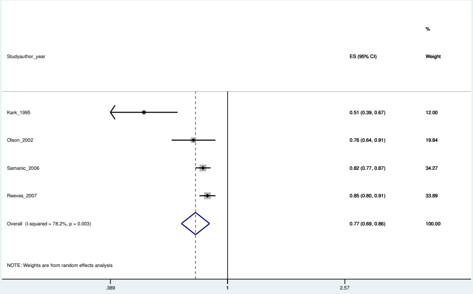


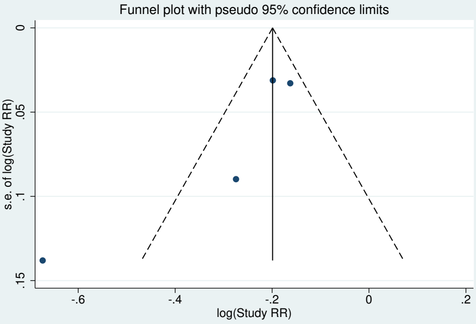


BMI per 5kg/m2: Lung cancer inc, non-smokers

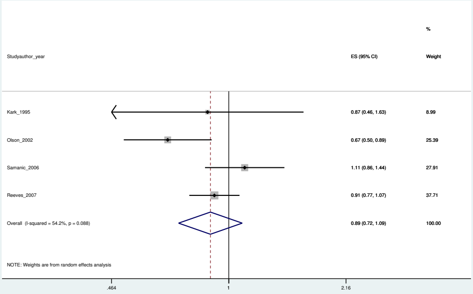


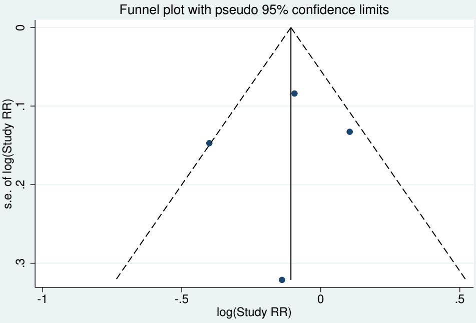

BMI per 5kg/m2: Melanoma inc, overall

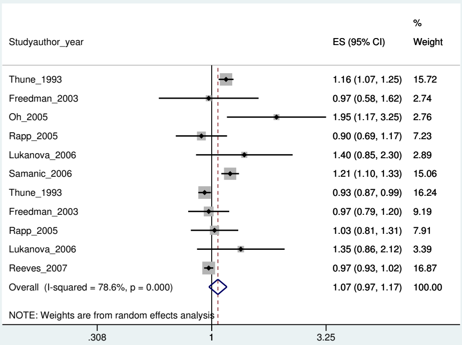


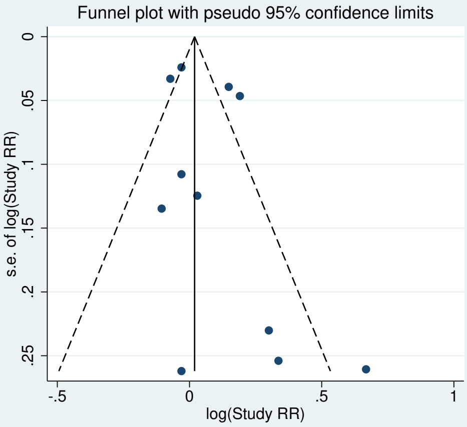


BMI per 5kg/m2: Melanoma inc, men

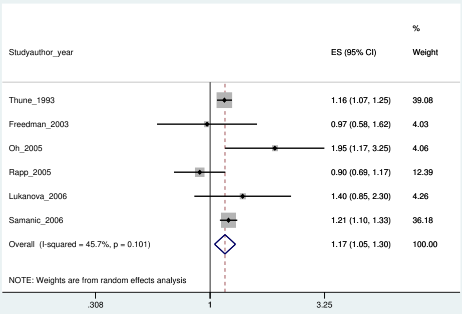


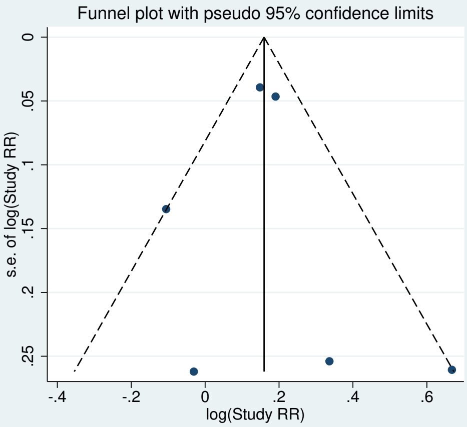


BMI per 5kg/m2: Melanoma inc, women

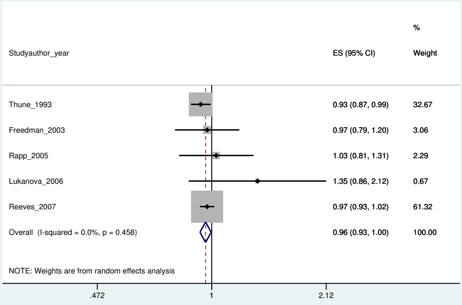


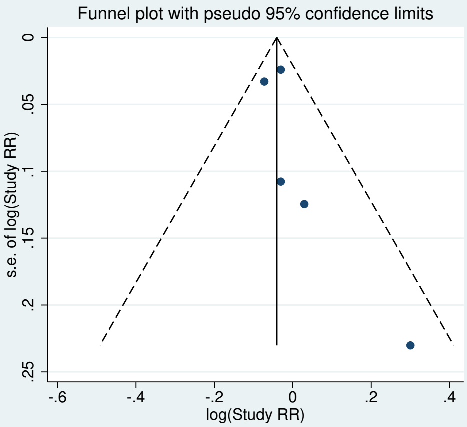

BMI per 5kg/m2: Breast cancer inc, premenopausal

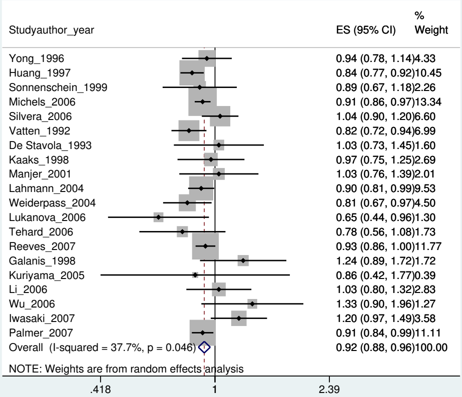


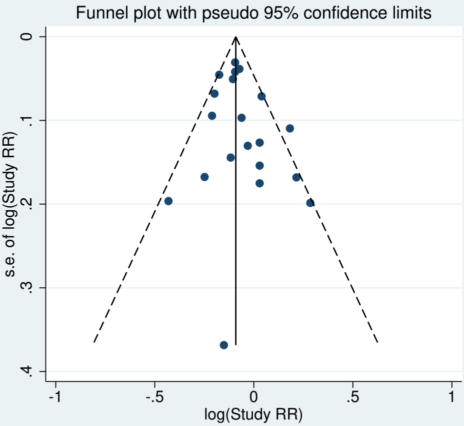


WG per 5kg: Breast cancer inc, premenopausal

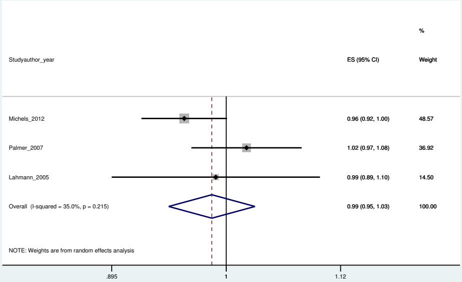


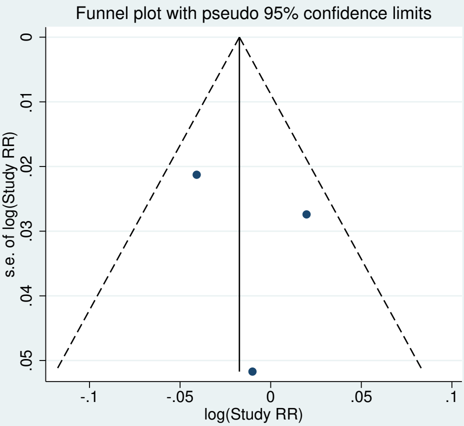


WHR per 0.1 units: Breast cancer inc, premenopausal

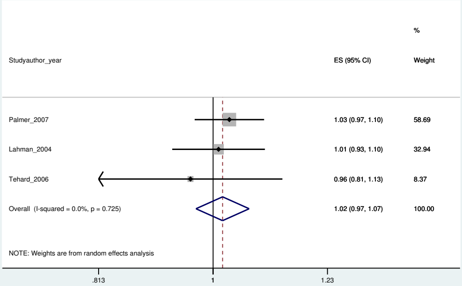


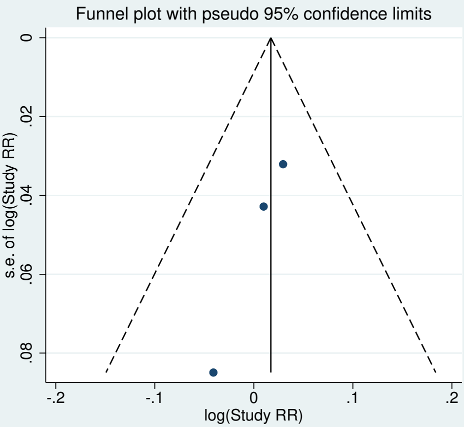

## BMI per 5kg/m2: Breast cancer inc, postmenopausal

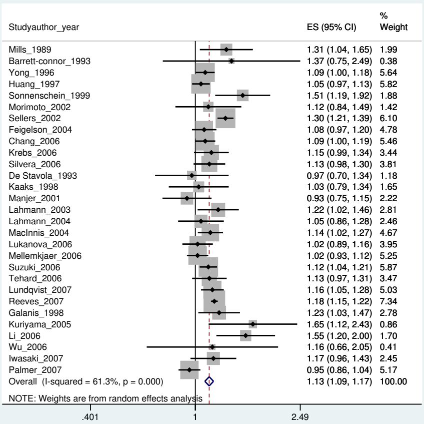


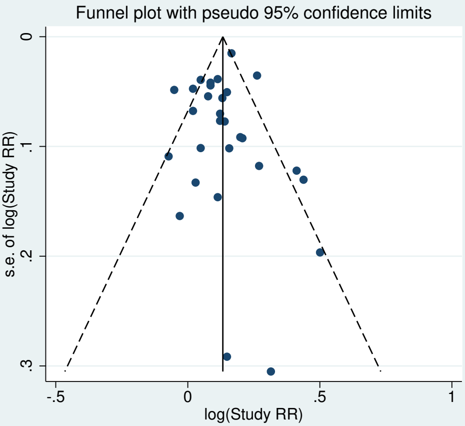


## WG per 5kg: Breast cancer inc, postmenopausal, HRT -

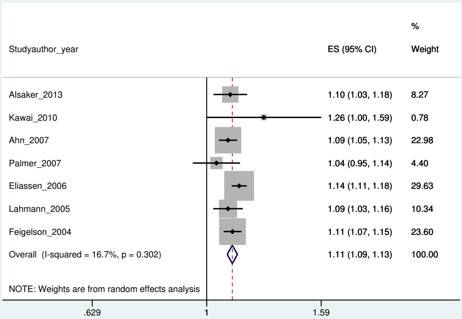


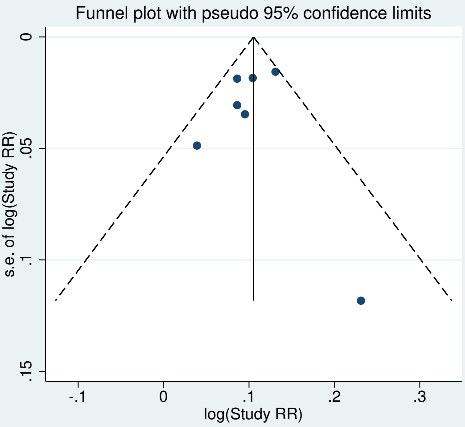


## WG per 5kg: Breast cancer inc, postmenopausal, HRT +

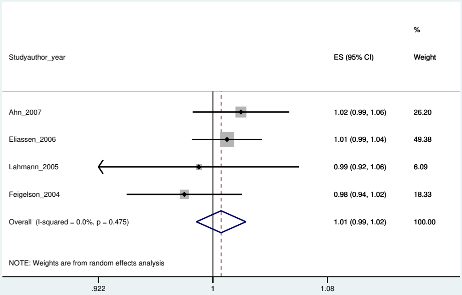


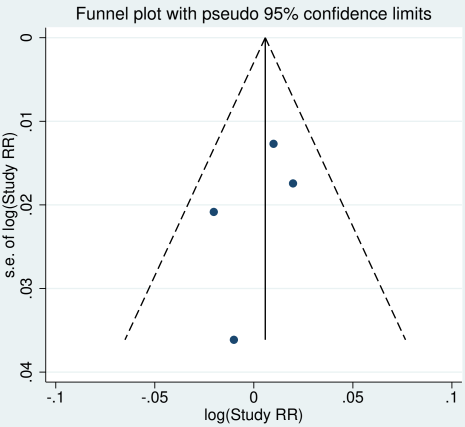

BMI iya, per 5kgm2: Endometrial cancer inc

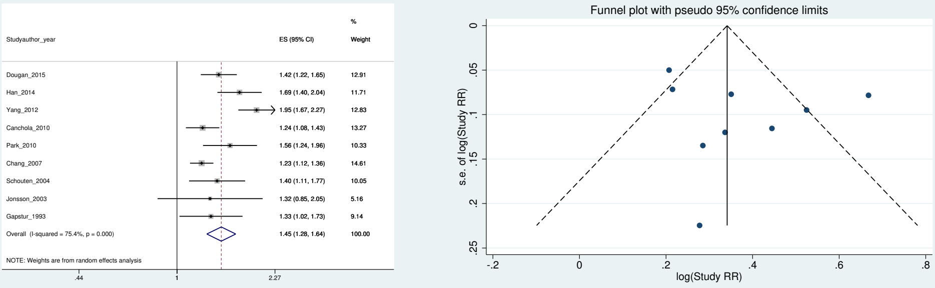


BMI per 5kg/m2: Endometrial cancer inc

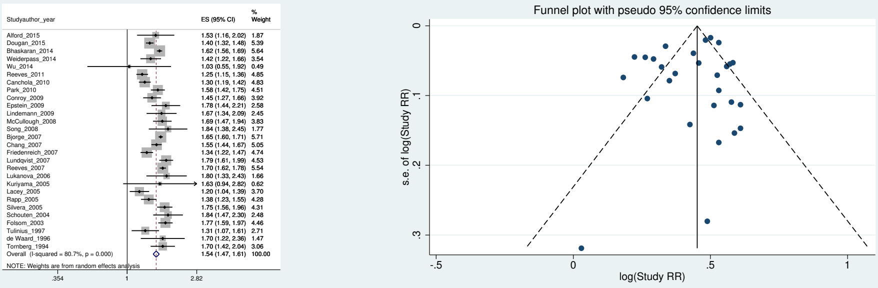


HC per 10cm: Endometrial cancer inc

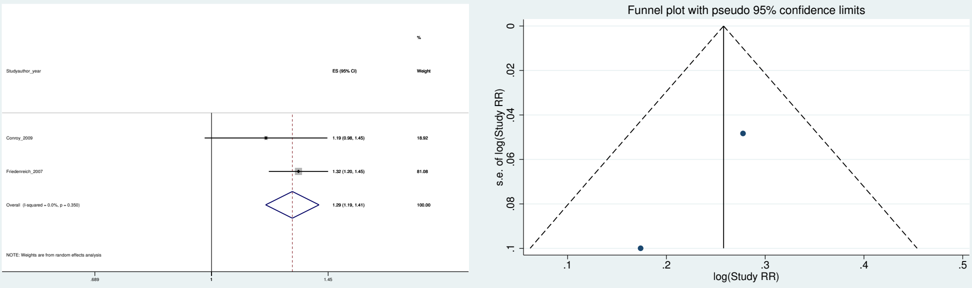


WG per 5kg: Endometrial cancer inc

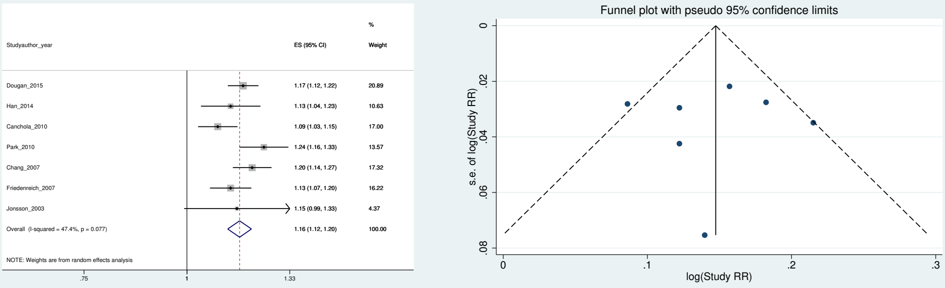


WC per 10 cm: Endometrial cancer inc

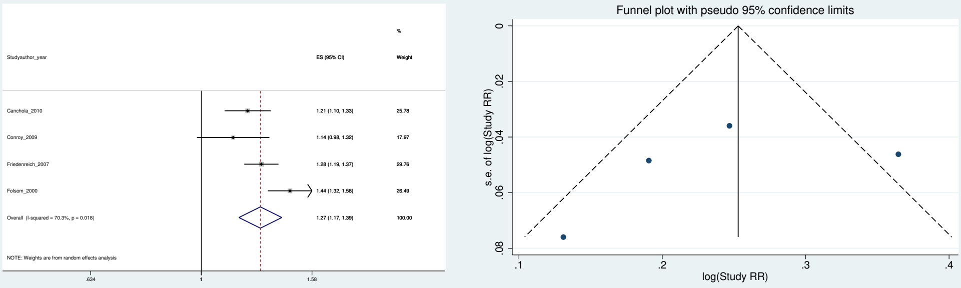


Weight per 5kg: Endometrial cancer inc

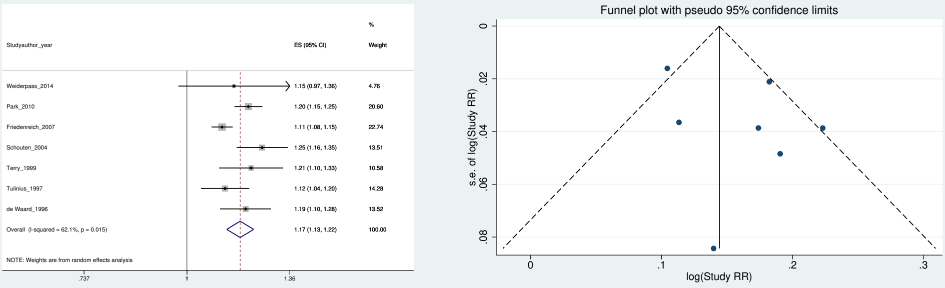


WHR per 0.1 units: Endometrial cancer inc

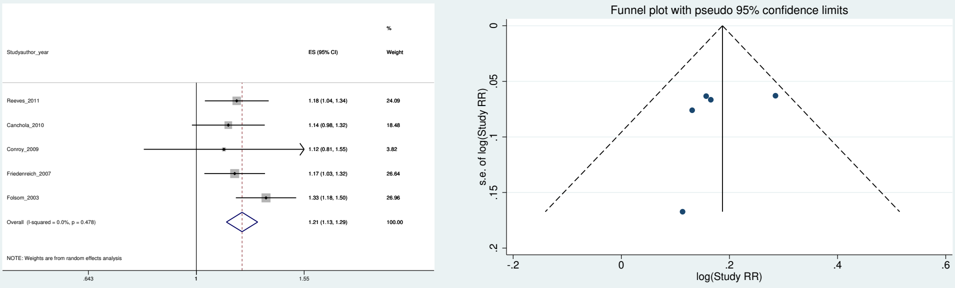


BMI per 5kg/m2: Endometrial cancer mortality

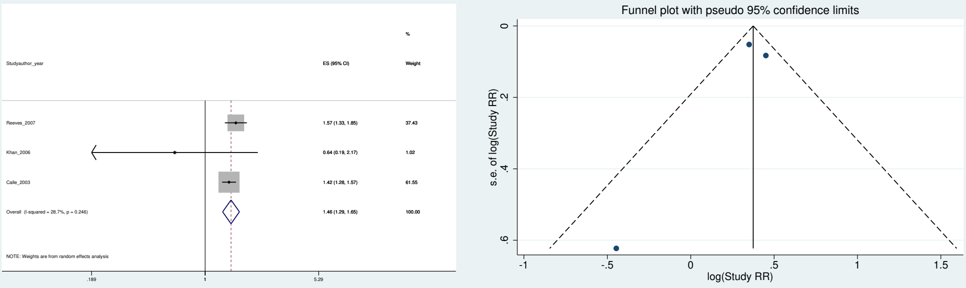

# BMI per 5kg/m2: Endometrial cancer inc, premenopausal

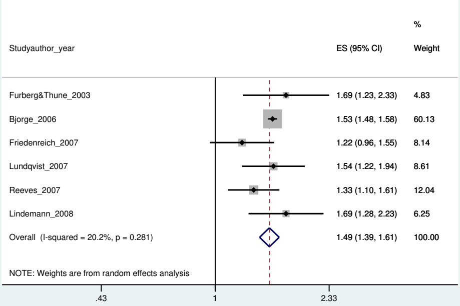


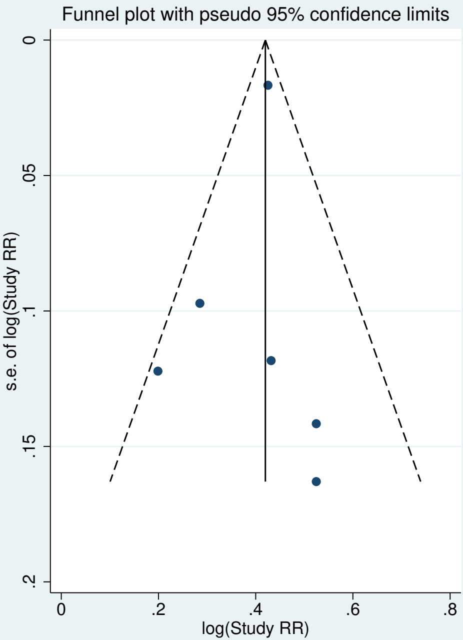

BMI per 5kg/m2: Endometrial cancer inc, postmenopausal

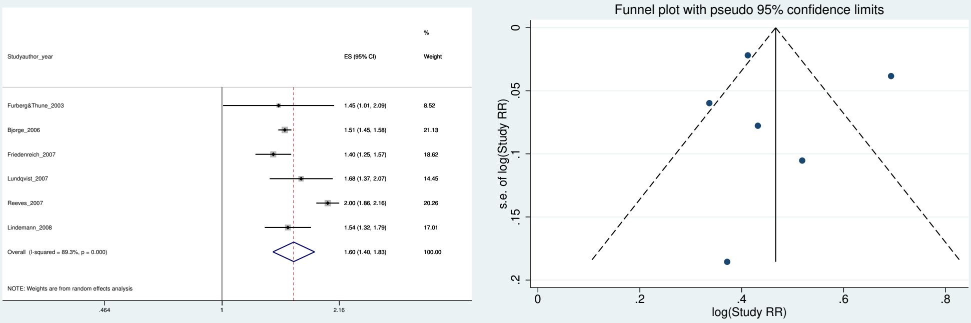


BMI per 5kg/m2: Endometrial cancer inc, postmenopausal HRT -

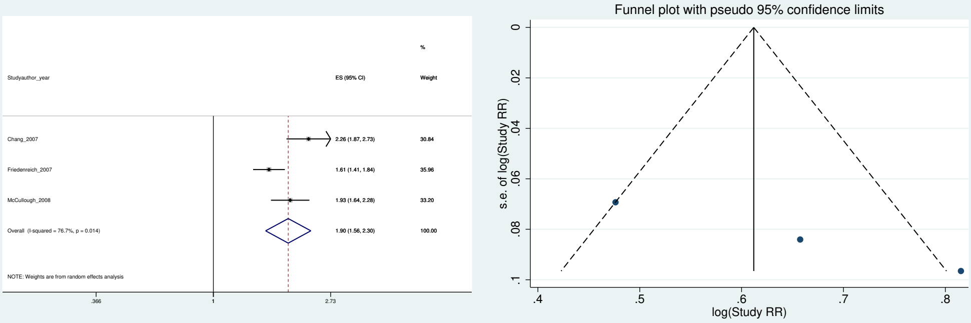


BMI per 5kg/m2: Endometrial cancer inc, postmenopausal HRT +

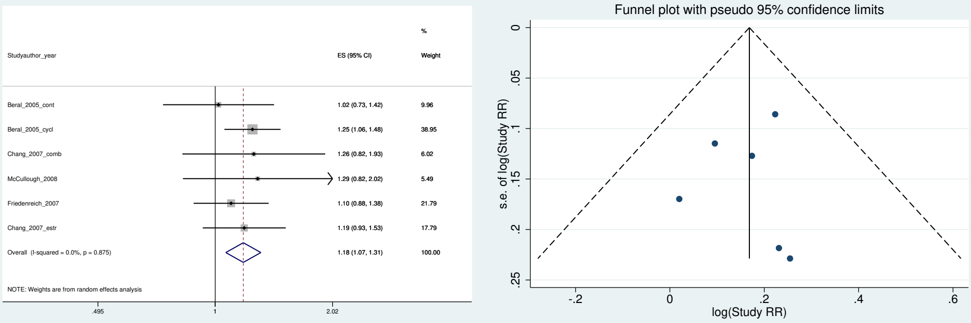


WG per 5kg: Endometrial cancer inc, postmenopausal, HRT -

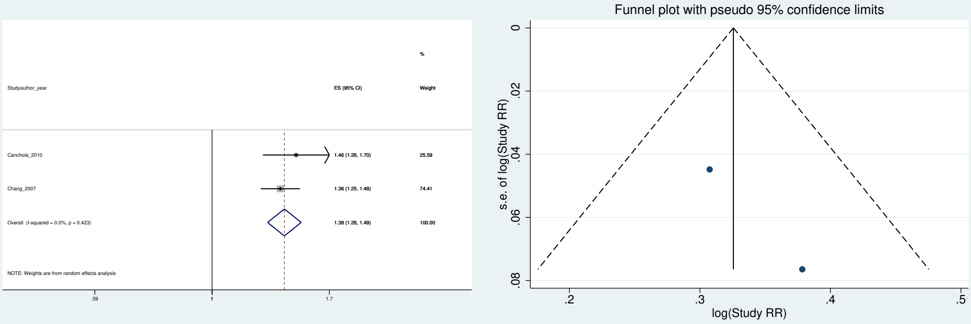


WG per 5kg: Endometrial cancer inc, postmenopausal, HRT +

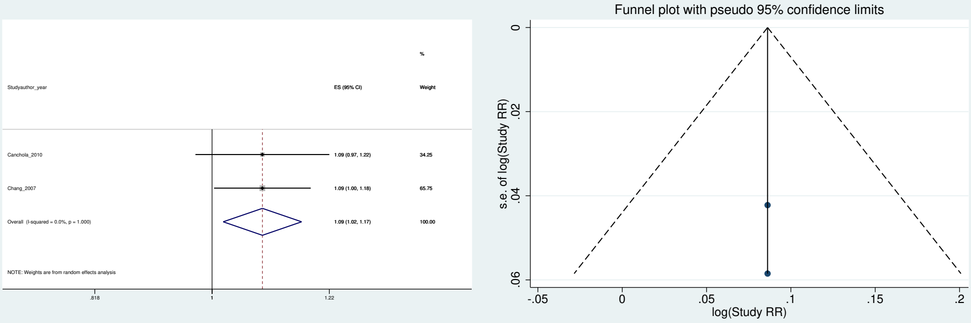

BMI per 5kg/m2: Endometrial cancer inc, Type I

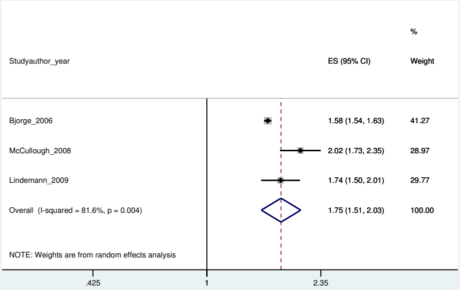


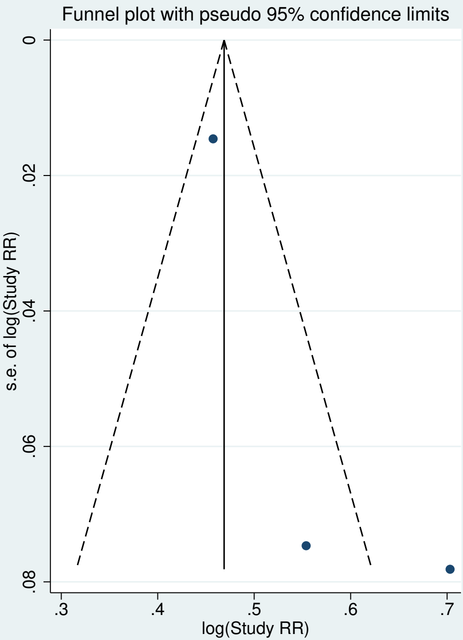

# BMI per 5kg/m2: Endometrial cancer inc, Type II

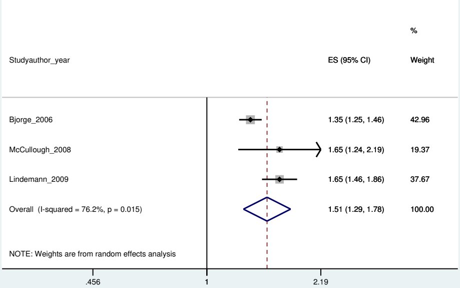


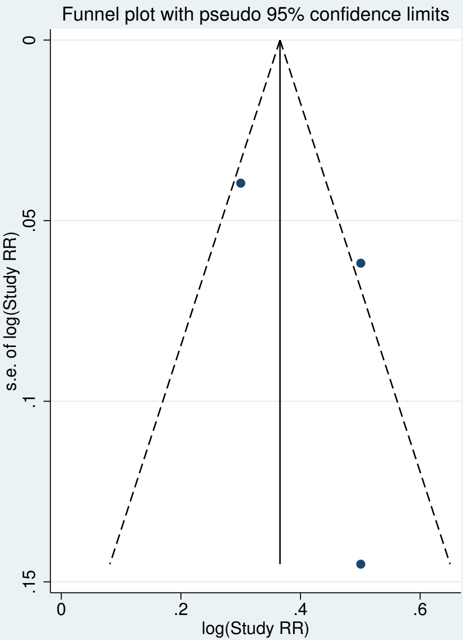

BMI iya, per 5kgm2: Ovarian cancer inc

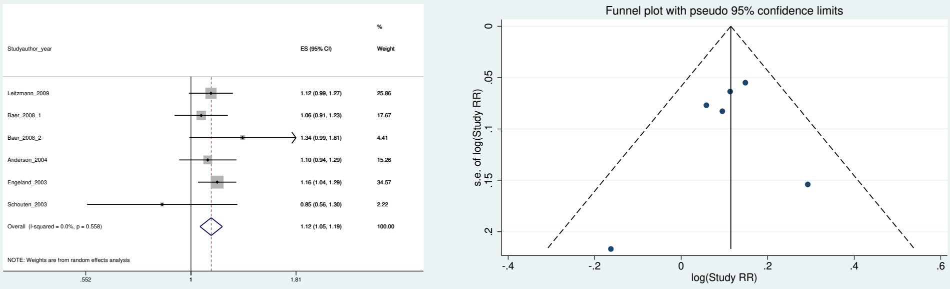


BMI per 5kg/m2: Ovarian cancer inc

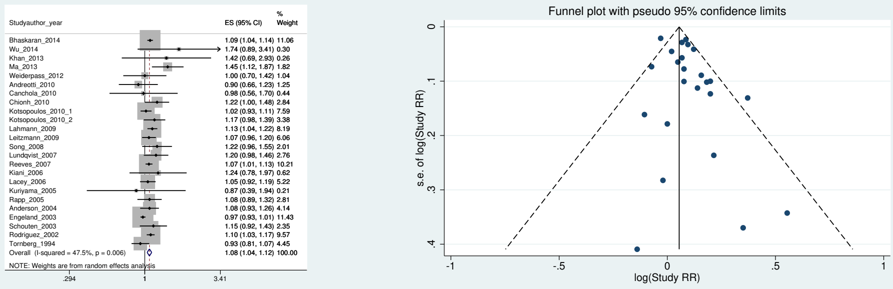


HC per 10cm: Ovarian cancer inc

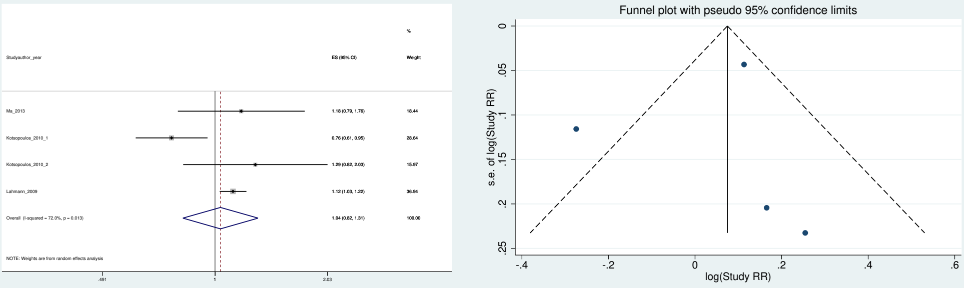


WC per 10 cm: Ovarian cancer inc

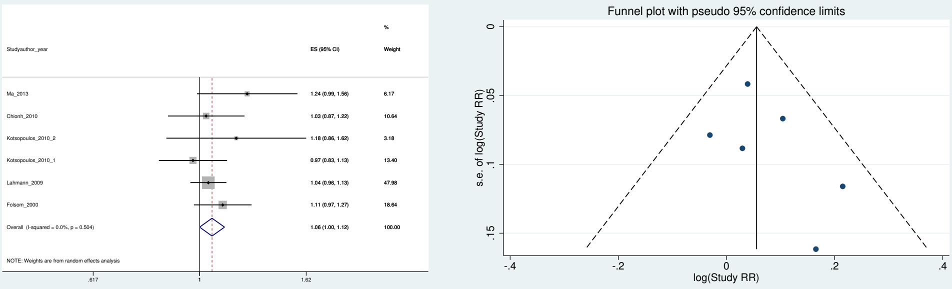


WG per 5kg: Ovarian cancer inc

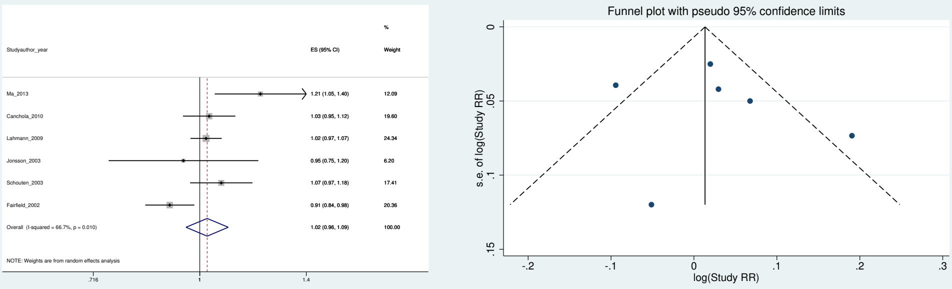


Weight per 5kg: Ovarian cancer inc

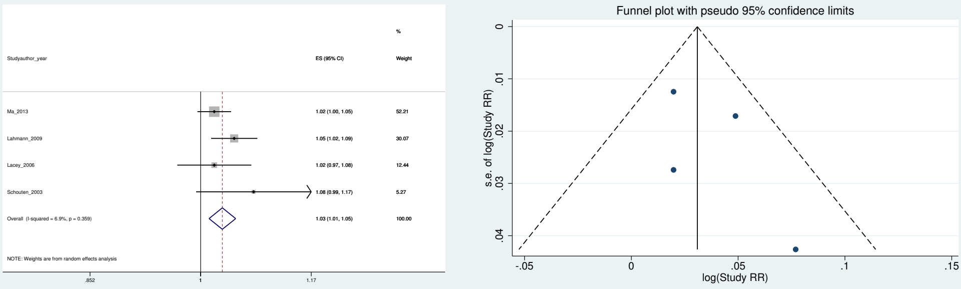


WHR per 0.1 units: Ovarian cancer inc

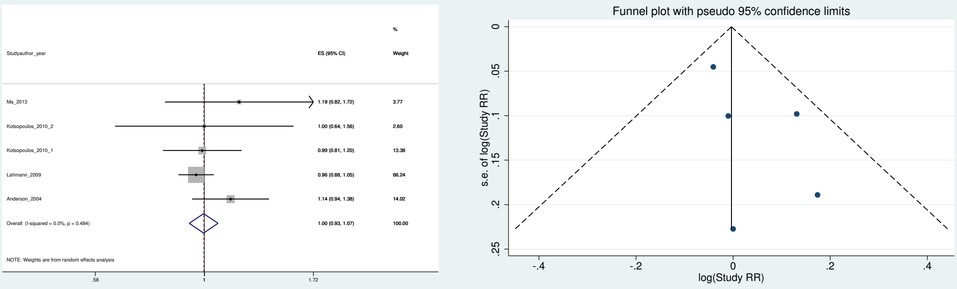


WG per 5kg: Ovarian cancer inc, postmenopausal, HRT-

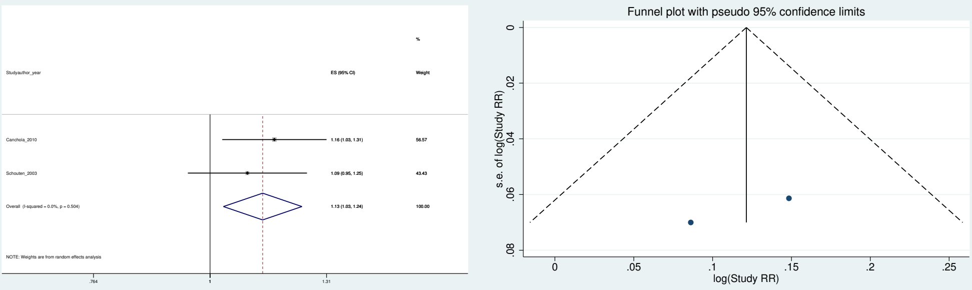

BMI per 5kg/m2: Prostate cancer inc, overall

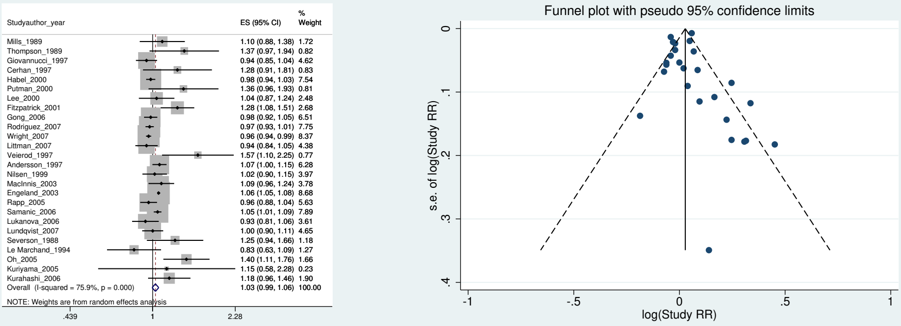


WC per 10 cm: Prostate cancer inc, overall

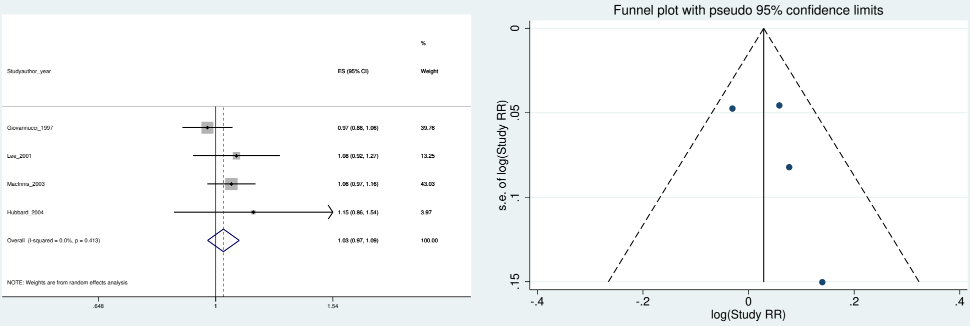


WG per 5kg: Prostate cancer inc, overall

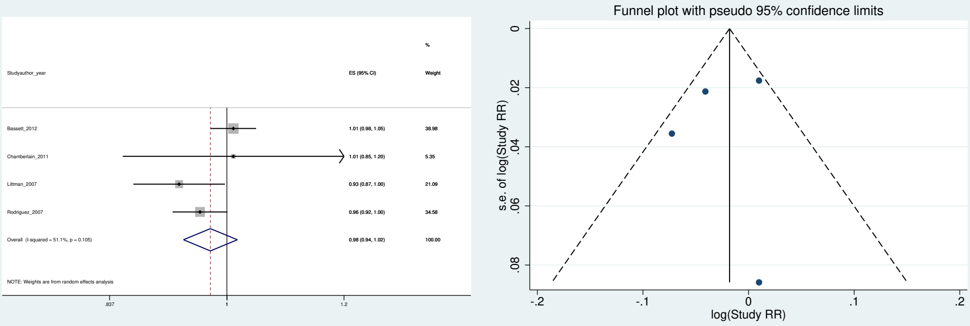


BMI per 5kg/m2: Prostate cancer mortality

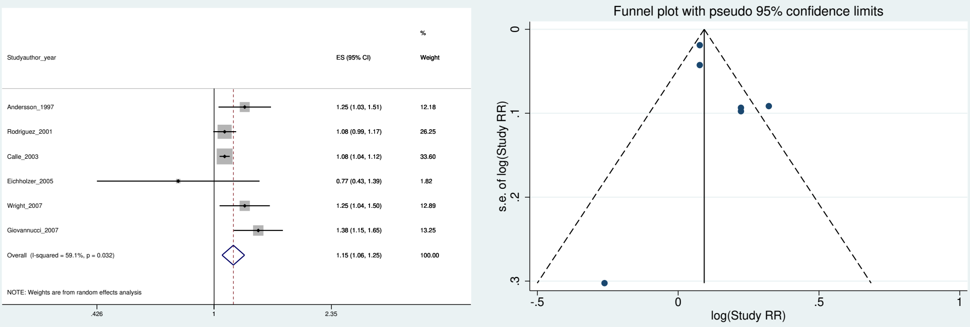


WG per 5kg: Prostate cancer inc, high screening rate

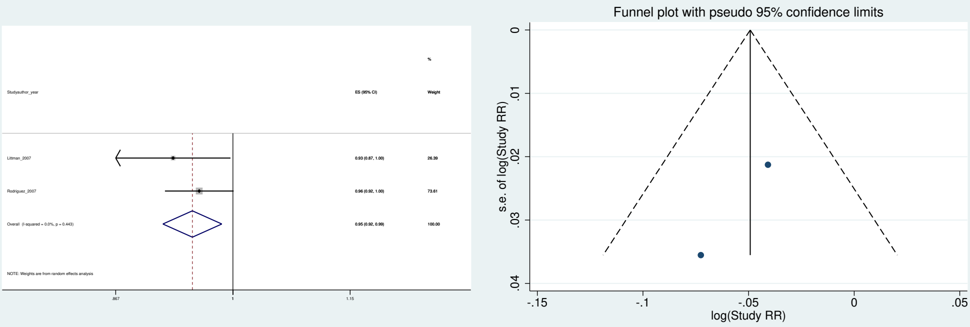


WG per 5kg: Prostate cancer inc, low screening rate

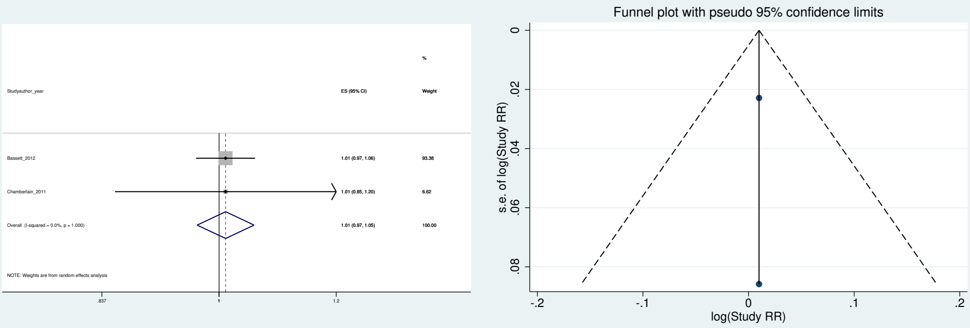

BMI per 5kg/m2: Prostate cancer inc, advanced

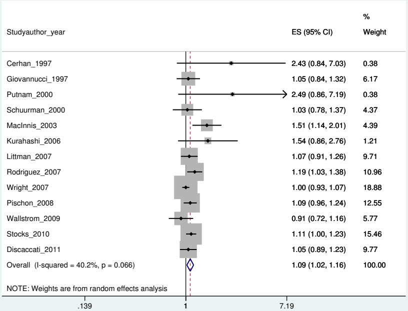


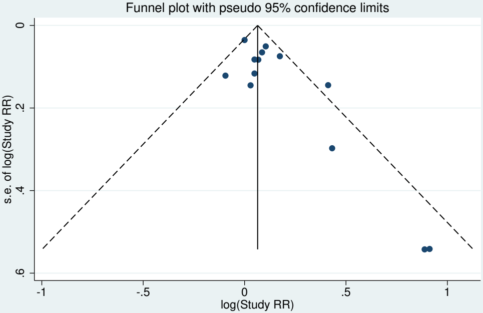


WG per 5kg: Prostate cancer inc, advanced

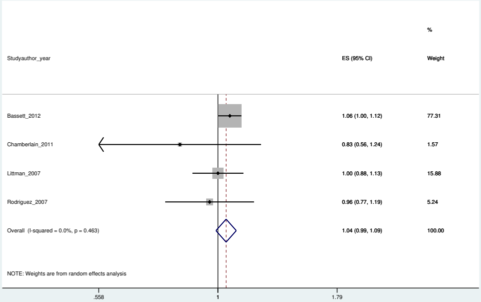


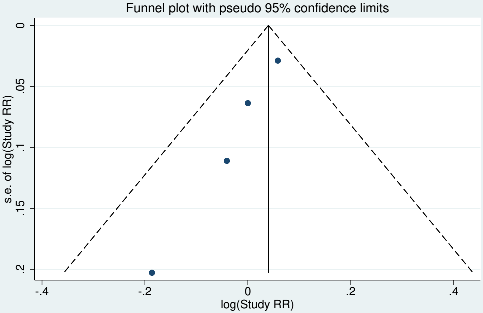

BMI per 5kg/m2: Prostate cancer inc, localized

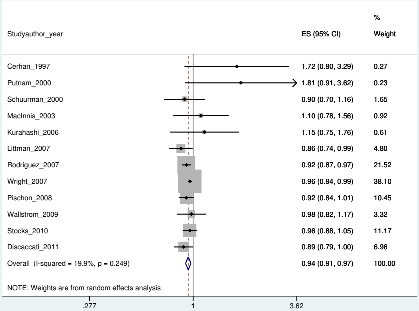


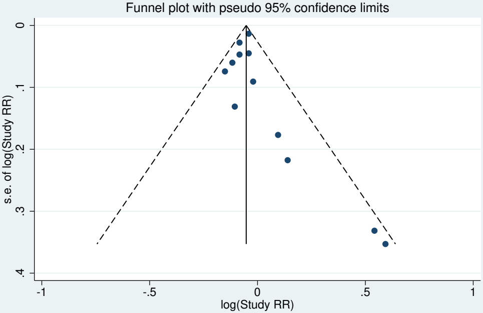


WG per 5kg: Prostate cancer inc, localized

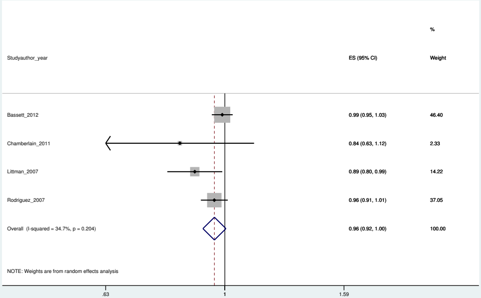


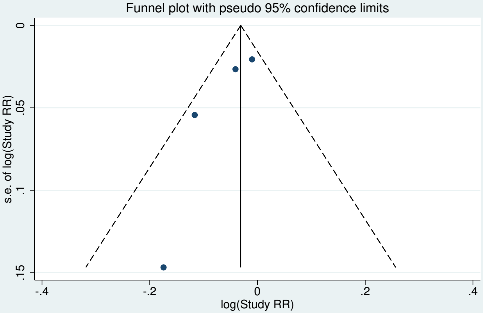

BMI per 5kg/m2: Kidney / renal cancer inc, men

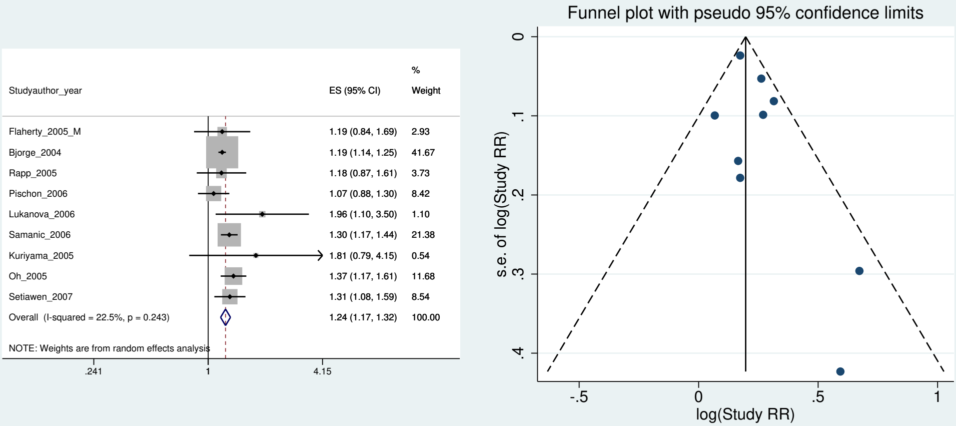


BMI per 5kg/m2: Kidney / renal cancer inc, women

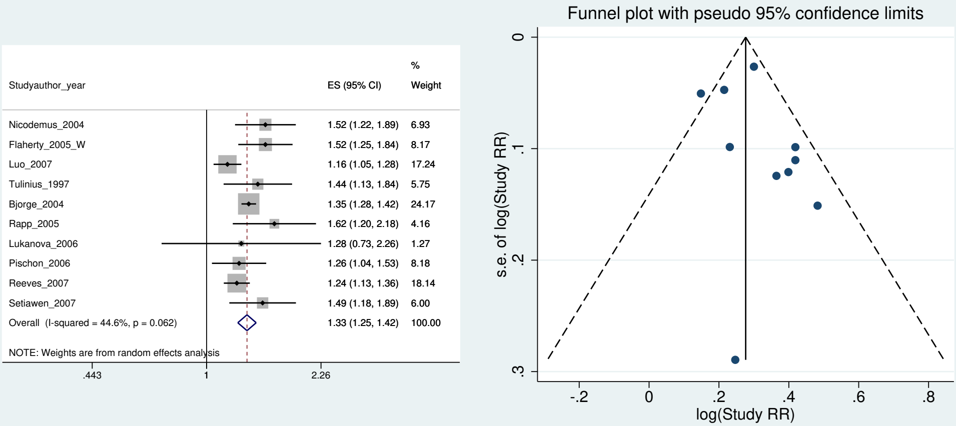


BMI per 5kg/m2: Kidney / renal cancer inc, overall

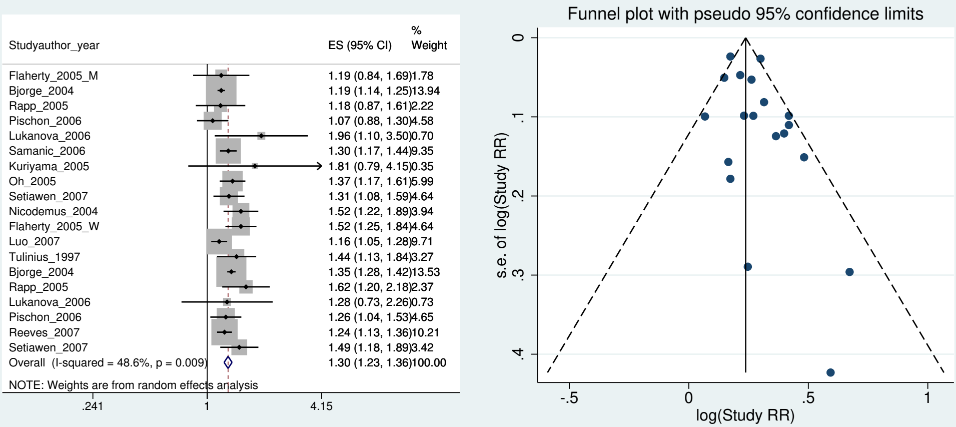

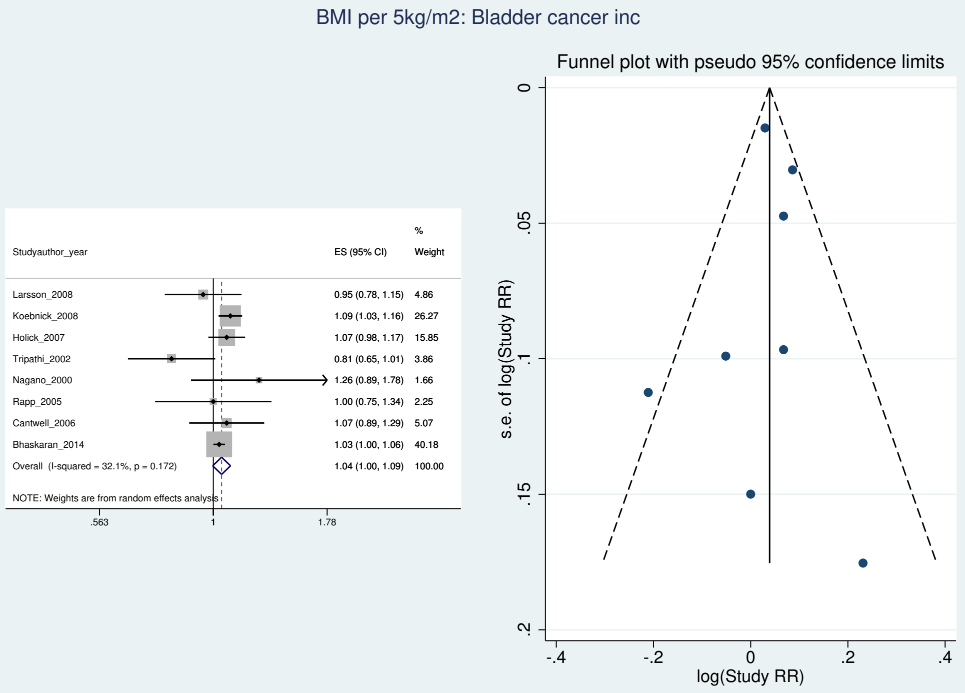
BMI per 5kg/m2: Bladder cancer inc
Studyauthor_year
ES (95% CI)
% Weight
Larsson_2008 0.95 (0.78, 1.15) 4.86
Koebnick_2008 1.09 (1.03, 1.16) 26.27
Holick_2007 1.07 (0.98, 1.17) 15.85
Tripathi_2002 0.81 (0.65, 1.01) 3.86
Nagano_2000 1.26 (0.89, 1.78) 1.66
Rapp_2005 1.00 (0.75, 1.34) 2.25
Cantwell_2006 1.07 (0.89, 1.29) 5.07
Bhaskaran_2014 1.03 (1.00, 1.06) 40.18
Overall (I-squared = 32.1%, p = 0.172) 1.04 (1.00, 1.09) 100.00
NOTE: Weights are from random effects analysis
.563
1
1.78
Funnel plot with pseudo 95% confidence limits
s.e. of log(Study RR)
0
.05
.1
.15
.2
-.4
-.2
0
.2
.4
log(Study RR)

BMI per 5kg/m2: Thyroid cancer inc, overall

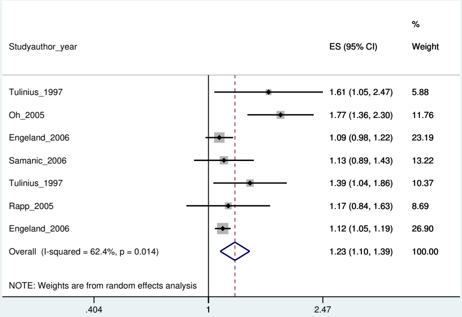


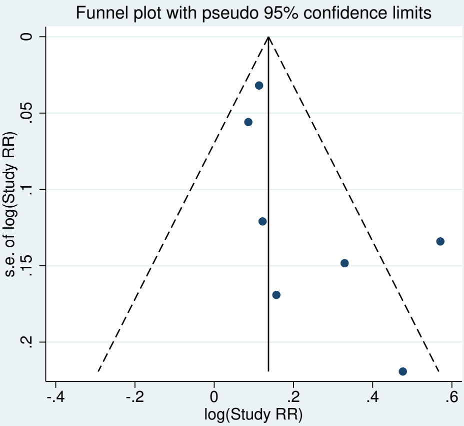


BMI per 5kg/m2: Thyroid cancer inc, men

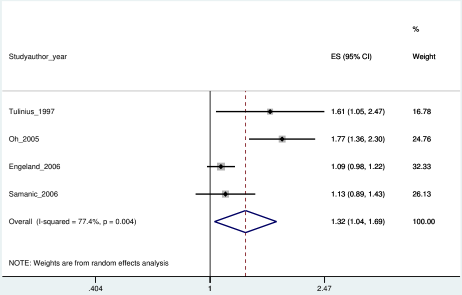


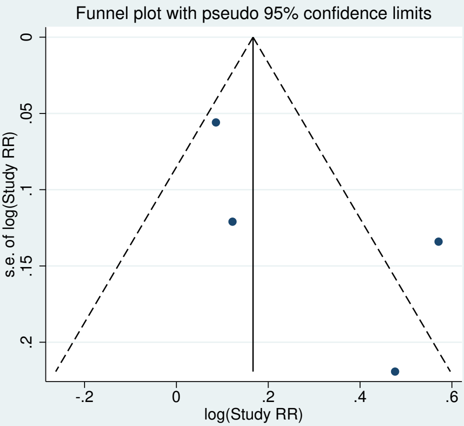


BMI per 5kg/m2: Thyroid cancer inc, women

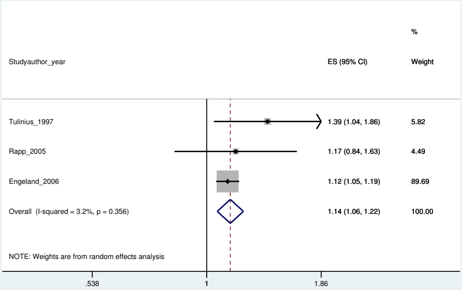


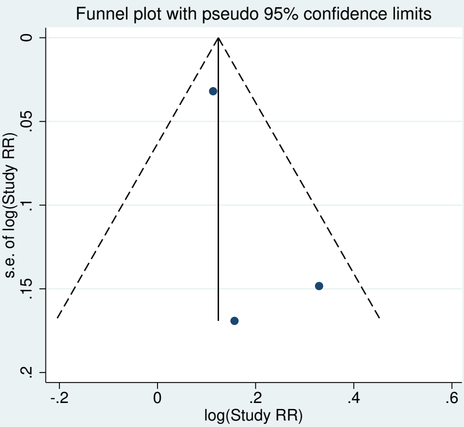

BMI per 5kg/m2: Non-Hodgkin lymphoma inc

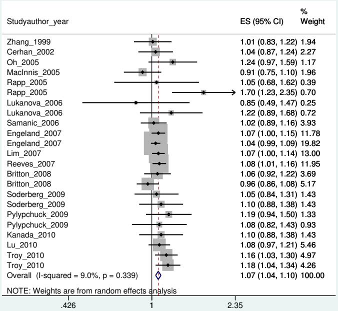


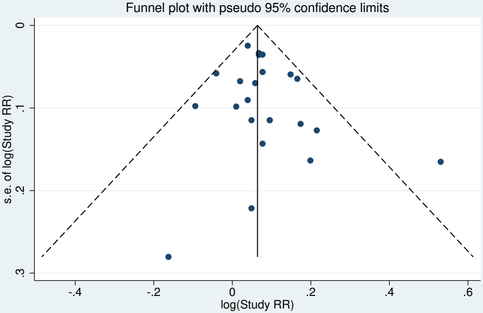


BMI per 5kg/m2: Non-Hodgkin lymphoma mortality

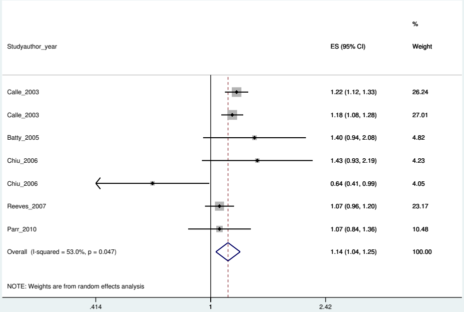


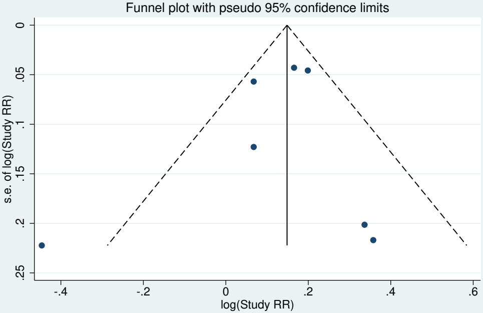

BMI per 5kg/m2: Multiple myeloma inc, overall

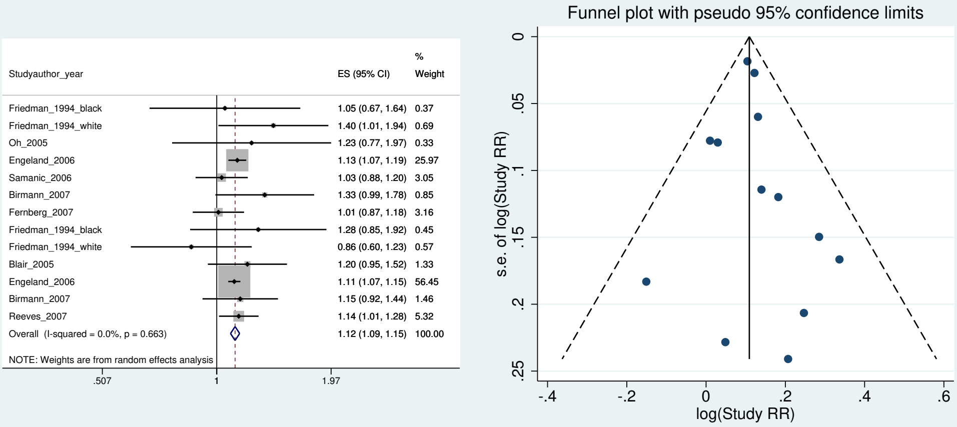


BMI per 5kg/m2: Multiple myeloma inc, men

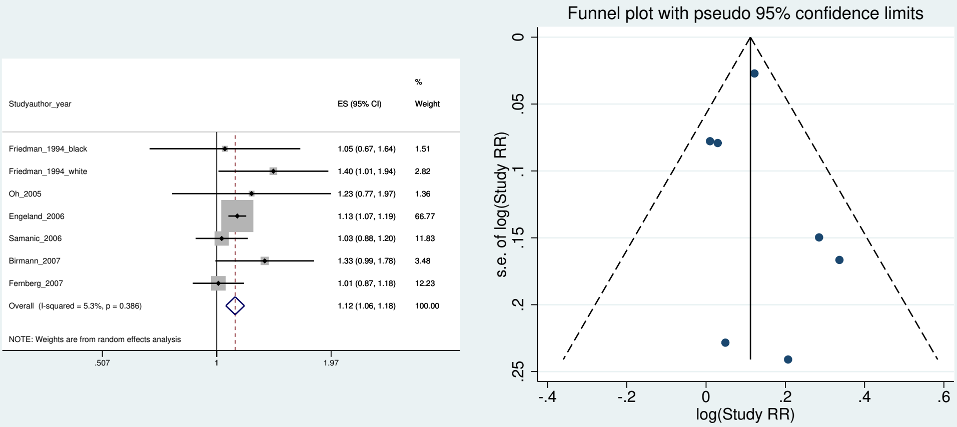


BMI per 5kg/m2: Multiple myeloma inc, women

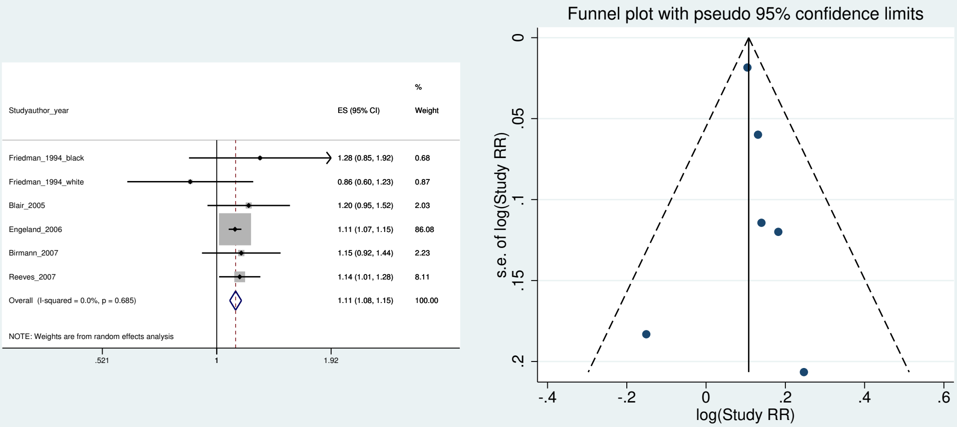

BMI per 5kg/m2: Leukemia inc, men

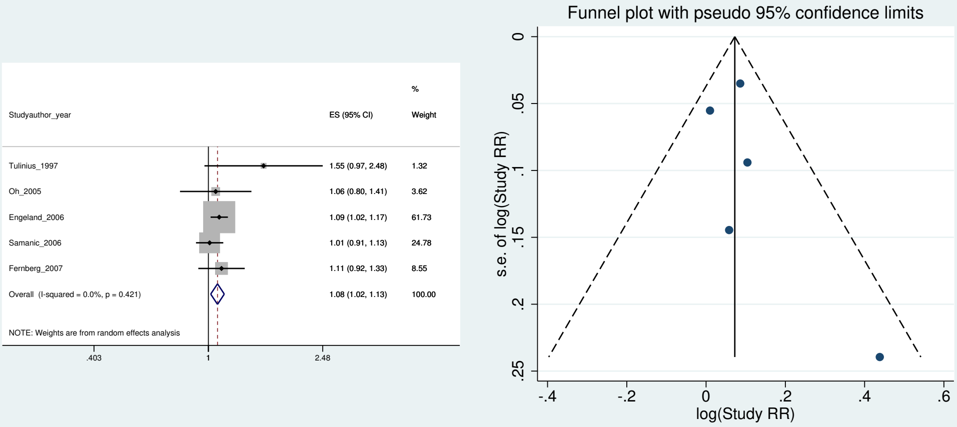


BMI per 5kg/m2: Leukemia inc, women

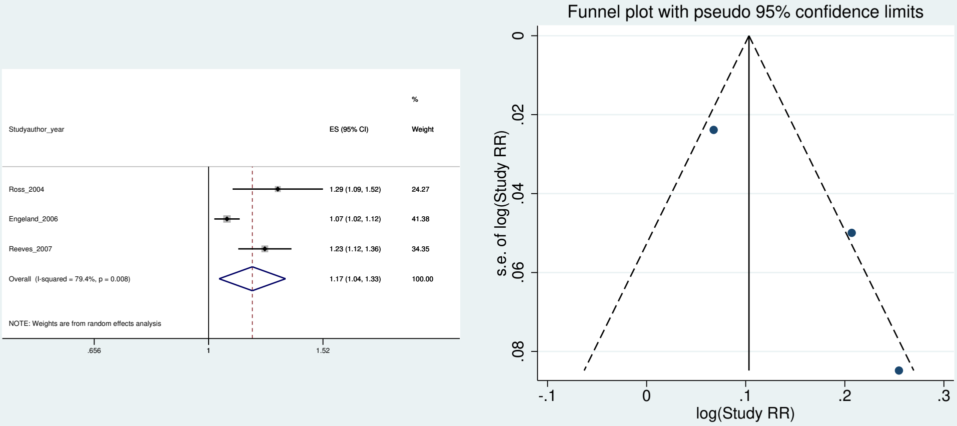


BMI per 5kg/m2: Leukemia inc, overall

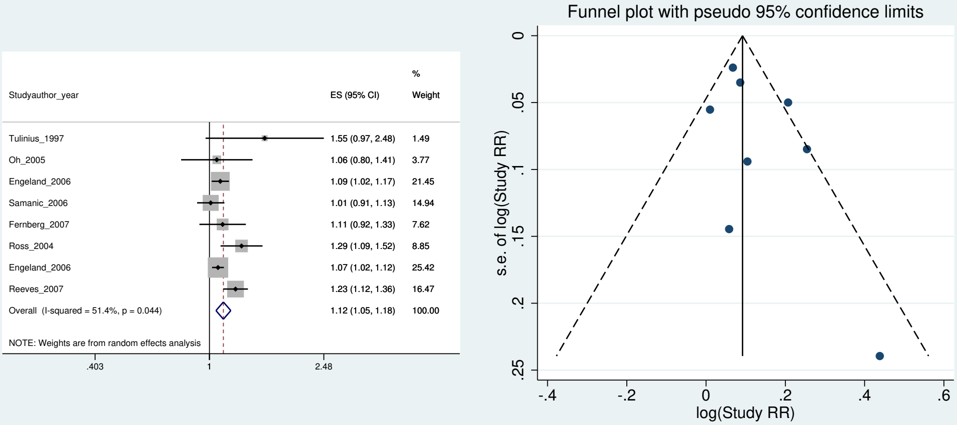

Supplement: Supplementary file 1 — Supplementary figures [file kyrm035374.ww1.pdf]
