# Supplementary material for: Adiposity and cancer at major anatomical sites: umbrella review of the literature
Source: BMJ. 2017 Feb 28;356:j477. doi: 10.1136/bmj.j477 (PMC5421437; doi:10.1136/bmj.j477)
Supplement: Supplementary file 2 — Supplementary tables and methods [file kyrm035374.ww2.pdf]

Supplementary table 1: Description of the 194* meta-analyses of obesity and cancer incidence or mortality — only cohort studies included.

Author, year	Exposure	Association of obesity or weight change with	ICD-10	N	Sample size cases / cohort	Summary relative risk (95% CI) Fixed Effects[‡]	Random effects[§]	Largest Study[†]	Fixed P-value[¶]	Random P-value[||]	95% Prediction interval[#]
Casagrande 2014	BS, inc density rate after surgery	Overall CA inc	C00-C97	9	725 / 31,479	2.23 (2.08-2.40)	1.10 (0.67-1.80)	2.51 (2.24-2.81)	<1E-100	0.70	0.21-5.91
Casagrande 2014	BS; surgery vs. no surgery	Overall CA inc	C00-C97	4	1,745 / 31,807	0.61 (0.54-0.69)	0.40 (0.22-0.71)	0.75 (0.64-0.88)	7.1E-15	1.8E-3	0.03-6.14
Renehan 2008	BMI per $5kg/m^2$ increase	Oesophageal AdCa inc	C15	8	1,918 / 6,430,994	1.53 (1.41-1.67)	1.53 (1.41-1.67)	1.67 (1.44-1.92)	1.37E-22	1.37E-22	1.38-1.71
Singh 2013	Central adiposity	Oesophageal AdCa inc	C15	2	341 / 565,067	1.78 (1.26-2.52)	1.78 (1.26-2.52)	1.81 (1.24-2.64)	0.001	0.001	NA
Renehan 2008	BMI per $5kg/m^2$ increase	Oesophageal AdCa inc, men	C15	5	996 / 3,196,747	1.55 (1.39-1.73)	1.52 (1.33-1.74)	1.67 (1.44-1.92)	7.95E-16	1.26E-9	1.09-2.11
Renehan 2008	BMI per $5kg/m^2$ increase	Oesophageal AdCa inc, women	C15	3	922 / 3,234,247	1.51 (1.30-1.75)	1.51 (1.30-1.75)	1.54 (1.26-1.89)	2.76E-8	2.76E-8	0.59-3.86
Renehan 2008	BMI per $5kg/m^2$ increase	Oesophageal SqCa inc.	C15	5	3,545 / 6,369,699	0.67 (0.63-0.72)	0.63 (0.53-0.75)	0.77 (0.70-0.85)	8.53E-31	1.71E-7	0.34-1.15
Renehan 2008	BMI per $5kg/m^2$ increase	Oesophageal SqCa inc., men	C15	3	1,787 / 3,145,452	0.75 (0.68-0.81)	0.71 (0.61-0.84)	0.77 (0.70-0.85)	4.27E-11	6.1E-5	0.14-3.69
Renehan 2008	BMI per $5kg/m^2$ increase	Oesophageal SqCa inc., women	C15	2	1,758 / 3,224,247	0.58 (0.52-0.64)	0.57 (0.48-0.67)	0.61 (0.54-0.70)	2.76E-24	1.16E-10	NA
Turati 2013	BMI 30+ vs. < 25	Gastrc cardia and oesophageal AdCa	C15-C16.0	9	2,391 / 5,887,984	2.13 (1.88-2.42)	2.19 (1.84-2.58)	1.93 (1.56-2.39)	7.3E-32	9.4E-20	1.46-3.25
Turati 2013	BMI 25-29.99 vs. < 25	Gastrc cardia and oesophageal AdCa	C15-C16.0	10	2,832 / 6,265,992	1.48 (1.35-1.61)	1.48 (1.35-1.61)	1.35 (1.11-1.64)	3.3E-17	3.3E-17	1.33-1.64
Lin 2014	BMI 25-29.99 vs. < 25	Gastric CA inc	C16	12	24,759 / 4,853,037	0.99 (0.94-1.04)	1.00 (0.93-1.08)	0.97 (0.87-1.09)	0.66	0.92	0.85-1.19
Lin 2014	BMI 30+ vs. < 25	Gastric CA inc	C16	11	25,581 / 5,144,097	1.10 (1.01-1.21)	1.10 (1.00-1.22)	1.23 (1.02-1.49)	0.04	0.05	0.95-1.29
Renehan 2008	BMI per $5kg/m^2$ increase	Gastric CA inc	C16	13	8,293 / 2,935,218	0.97 (0.93-1.01)	0.99 (0.92-1.06)	0.95 (0.90-1.00)	0.09	0.75	0.83-1.17
Renehan 2008	BMI per $5kg/m^2$ increase	Gastric CA inc, men	C16	8	6,693 / 1,460,332	0.96 (0.91-1.00)	0.97 (0.88-1.06)	0.95 (0.90-1.00)	0.05	0.48	0.77-1.21
Renehan 2008	BMI per $5kg/m^2$ increase	Gastric CA inc, women	C16	5	1,600 / 1,474,886	1.00 (0.92-1.09)	1.04 (0.91-1.21)	0.95 (0.86-1.05)	1.00	0.55	0.71-1.55
Larsson 2007	BMI per $5kg/m^2$ increase	Colon CA inc	C18	44	82,766 / 9,434,650	1.13 (1.11-1.14)	1.22 (1.17-1.28)	1.03 (1.01-1.05)	<1E-100	3.9E-16	0.95-1.57
Moghaddam 2007	BMI 30+ vs. < 25	Colon CA inc	C18	28	37,136 / 3,946,598	1.22 (1.18-1.26)	1.44 (1.28-1.62)	1.07 (1.02-1.12)	5.9E-28	1.8E-9	0.91-2.28
Larsson 2007	WC per 10 cm increase	Colon CA inc	C18	8	3,371 / 869,185	1.21 (1.15-1.28)	1.25 (1.15-1.35)	1.14 (1.05-1.25)	5.64E-14	2.98E-08	1.01-1.54
Keum 2015	WG per 5kg increase	Colon CA inc	C18	7	2.909 / 298,174	1.07 (1.03-1.10)	1.07 (1.03-1.10)	1.10 (1.03-1.17)	8.3E-5	8.3E-5	1.02-1.11

Larsson 2007	WHR per 0.1 unit increase	Colon CA inc	C18	8	3,132 / 941,955	1.26 (1.18-1.35)	1.29 (1.17-1.43)	1.24 (1.10-1.39)	3.22E-12	1.11E-06	0.97-1.72
Larsson 2007	BMI per 5kg/m^2 increase	Colon CA inc, men	C18	24	42,821 / 5,213,139	1.29 (1.26-1.32)	1.30 (1.25-1.35)	1.27 (1.23-1.31)	<1E-100	<1E-100	1.19-1.42
Moghaddam 2007	BMI 30+ vs. < 25	Colon CA inc, men	C18	14	17,387 / 2,192,572	1.53 (1.44-1.63)	1.53 (1.44-1.63)	1.49 (1.39-1.60)	<1E-100	<1E-100	1.43-1.64
Larsson 2007	WC per 10 cm increase	Colon CA inc, men	C18	5	1,869 / 469,320	1.31 (1.21-1.42)	1.33 (1.18-1.50)	1.15 (1.01-1.31)	6.68E-11	1.63E-06	0.95-1.86
Keum 2015	WG per 5kg increase	Colon CA inc, men	C18	4	1,718 / 142,085	1.09 (1.05-1.14)	1.09 (1.05-1.14)	1.10 (1.03-1.17)	4.1E-5	4.1E-5	1.00-1.19
Larsson 2007	WHR per 0.1 unit increase	Colon CA inc, men	C18	4	1,563 / 461,754	1.38 (1.23-1.54)	1.43 (1.19-1.71)	1.24 (1.05-1.46)	1.08E-8	1.0E-5	0.71-2.87
Larsson 2007	BMI per 5kgm2 increase	Colon CA inc, women	C18	20	39,945 / 4,221,511	1.05 (1.03-1.07)	1.12 (1.06-1.17)	1.03 (1.01-1.05)	2.3E-9	6.9E-6	0.96-1.30
Moghaddam 2007	BMI 30+ vs. < 25	Colon CA inc, women	C18	14	19,749 / 1,754,026	1.09 (1.05-1.14)	1.22 (1.07-1.40)	1.07 (1.02-1.12)	4.3E-5	3.4E-3	0.85-1.75
Larsson 2007	WC per 10 cm increase	Colon CA inc, women	C18	3	1,502 / 399,865	1.16 (1.08-1.23)	1.16 (1.08-1.23)	1.14 (1.05-1.25)	1.1E-5	1.1E-5	0.76-1.76
Keum 2015	WG per 5kg increase	Colon CA inc, women	C18	3	1,191 / 156,089	1.03 (0.98-1.09)	1.03 (0.98-1.09)	1.00 (0.93-1.07)	0.24	0.24	0.74-1.44
Larsson 2007	WHR per 0.1 unit increase	Colon CA inc, women	C18	4	1,569 / 480,201	1.20 (1.11-1.30)	1.20 (1.08-1.33)	1.24 (1.10-1.39)	9.2E-6	5.0E-5	0.86-1.69
Robsham 2013	BMI, Obese vs. normal**	Colon CA inc; distal colon	C18	20	4,359 / 1,320,435	1.52 (1.36-1.70)	1.59 (1.34-1.89)	0.96 (0.75-1.23)	1.2E-13	1.2E-7	0.94-2.69
Robsham 2013	BMI, Obese vs. normal**	Colon CA inc; distal colon, men	C18	7	1,685 / 643,569	1.67 (1.39-1.99)	1.70 (1.36-2.11)	1.77 (1.33-2.35)	1.9E-8	2.3E-6	1.11-2.60
Robsham 2013	BMI, Obese vs. normal**	Colon CA inc; distal colon, women	C18	12	2,565 / 669,300	1.44 (1.25-1.66)	1.53 (1.19-1.97)	0.96 (0.75-1.23)	5.8E-7	8.3E-4	0.74-3.19
Robsham 2013	BMI, Obese vs. normal**	Colon CA inc; proximal colon	C18	20	4,680 / 1,320,435	1.24 (1.10-1.39)	1.33 (1.12-1.57)	1.02 (0.84-1.24)	2.7E-4	9.6E-4	0.84-2.09
Robsham 2013	BMI, Obese vs. normal**	Colon CA inc; proximal colon, men	C18	7	1,720 / 643.569	1.44 (1.18-1.77)	1.44 (1.18-1.77)	1.18 (0.86-1.62)	4.4E-4	4.4E-4	1.10-1.89
Robsham 2013	BMI, Obese vs. normal**	Colon CA inc; proximal colon, women	C18	12	2,772 / 669,300	1.12 (0.97-1.29)	1.18 (0.96-1.44)	1.02 (0.84-1.24)	0.11	0.11	0.76-1.83
Yang 2015	BS; surgery vs. no surgery	Colorectal CA inc	C18-C20	5	1,013 / 182,298	0.77 (0.62-0.96)	0.77 (0.62-0.96)	0.79 (0.57-1.10)	0.02	0.02	0.54-1.10
Ma 2013	BMI, Obese vs. normal**	Colorectal CA inc	C18-C20	72	166,030 / 15,623,976	1.19 (1.17-1.22)	1.33 (1.25-1.42)	1.06 (1.02-1.10)	<1E-100	1.7E-19	0.94-1.50
Ma 2013	WC, highest vs. lowest category	Colorectal CA inc	C18-C20	19	12,566 / 2,041,675	1.44 (1.32-1.57)	1.45 (1.33-1.59)	1.32 (1.11-1.56)	1.8E-17	9.0E-16	1.24-1.70
Schlesinger 2015	WG per 1kg increase / year	Colorectal CA inc	C18-C20	17	27,638 / 1,974,918	1.22 (1.15-1.29)	1.20 (1.10-1.31)	1.31 (1.18-1.45)	1.1E-10	3.9E-5	0.95-1.52
Schlesinger 2015	WG: high gain vs. stable weight	Colorectal CA inc	C18-C20	19	28,530 / 2,110,272	1.18 (1.12-1.25)	1.18 (1.12-1.25)	1.26 (1.15-1.38)	3.7E-9	3.7E-9	1.11-1.26
Moghaddam 2007	BMI 30+ vs. < 25	Rectal CA inc	C20	21	39,384 / 6,768,071	1.14 (1.09-1.20)	1.21 (1.10-1.34)	1.04 (0.97-1.11)	1.4E-07	1.8E-4	0.97-1.52
Harriss 2009	BMI per 5kg/m^2 increase	Rectal CA inc	C20	31	43,196 / 7,495,868	1.05 (1.03-1.07)	1.07 (1.03-1.10)	1.01 (0.98-1.04)	3.2E-8	5.9E-5	0.98-1.16

Harriss 2009	BMI per 5kg/m^2 increase	Rectal CA inc, men	C20	17	23,167 / 4,293,489	1.09 (1.06-1.12)	1.09 (1.06-1.13)	1.08 (1.05-1.12)	1.4E-10	1.1E-7	1.04-1.15
Moghaddam 2007	BMI: > 30 vs. < 25	Rectal CA inc, men	C20	11	12,109 / 2,178,376	1.28 (1.19-1.39)	1.28 (1.19-1.39)	1.27 (1.16-1.38)	4.8E-10	4.8E-10	1.17-1.40
Harriss 2009	BMI per 5kg/m^2 increase	Rectal CA inc, women	C20	14	20,029 / 3,202,379	1.02 (0.99-1.05)	1.02 (0.99-1.05)	1.01 (0.98-1.04)	0.20	0.20	0.99-1.05
Moghaddam 2007	BMI 30+ vs. < 25	Rectal CA inc, women	C20	10	8,648 / 1,698,601	1.06 (0.99-1.13)	1.09 (0.98-1.22)	1.04 (0.97-1.11)	0.08	0.12	0.90-1.32
Chen 2012	BMI 25-29.99 vs. < 25	Liver CA inc	C22	14	16,382 / 3,976,906	1.10 (1.05-1.15)	1.18 (1.06-1.31)	1.07 (1.00-1.15)	5.0E-5	1.7E-3	0.88-1.58
Chen 2012	BMI 30+ vs. < 25	Liver CA inc	C22	19	22,378 / 8,574,903	1.64 (1.54-1.75)	1.83 (1.59-2.11)	1.33 (1.19-1.48)	<1E-100	6.5E-17	1.10-3.04
Wang 2012	BMI per 5kg/m^2 increase	Liver CA inc	C22	19	17,281 / 4,977,849	1.22 (1.17-1.26)	1.37 (1.23-1.53)	1.12 (1.05-1.19)	2.3E-24	2.4E-8	0.90-2.07
Li 2014	BMI 25-29.99 vs. < 25	Cholangiocarcinoma inc	C22.1, C24	7	1,753 / 2,332,590	1.29 (1.11-1.51)	1.29 (1.11-1.51)	1.40 (1.11-1.72)	1.3E-3	1.3E-3	1.05-1.58
Li 2014	BMI 30+ vs. < 25	Cholangiocarcinoma inc	C22.1, C24	6	1,478 / 10,201,493	1.73 (1.32-2.28)	1.77 (1.32-2.38)	1.36 (0.92-2.02)	9.4E-5	1.5E-4	1.07-2.93
Larsson 2007	BMI 30+ vs. < 25	Gallbladder CA inc	C23	13	5,516 / 12,517,024	1.72 (1.55-1.92)	1.69 (1.48-1.92)	1.88 (1.60-2.21)	1.2E-23	3.6E-15	1.32-2.15
Park 2014	BMI per 5kg/m^2 increase	Biliary tract system CA inc	C23-C24	10	6,961 / 6,008,270	1.64 (1.46-1.83)	1.56 (1.34-1.81)	1.88 (1.60-2.21)	1.3E-17	1.3E-8	1.13-2.15
Yang 2015	BS; surgery vs. no surgery	Pancreatic CA inc	C25	2	37 / 22,819	1.17 (0.50-2.77)	0.86 (0.17-4.35)	1.61 (0.62-4.18)	0.72	0.86	NA
Alsamarrai 2014	BMI, 25+ vs. <25	Pancreatic CA inc	C25	9	8,133 / 1,986,283	1.37 (1.31-1.43)	1.30 (0.90-1.86)	1.24 (1.18-1.30)	<1E-100	0.16	0.35-4.87
Aune 2012	BMI per 5kg/m^2 increase	Pancreatic CA inc	C25	22	8,987 / 4,978,230	1.10 (1.07-1.13)	1.10 (1.06-1.14)	1.09 (1.03-1.16)	4.5E-11	2.2E-7	1.01-1.21
Aune 2012	WC per 10cm increase	Pancreatic CA inc	C25	5	1,365 / 1,154,996	1.11 (1.05-1.18)	1.11 (1.05-1.18)	1.08 (0.98-1.18)	2.0E-4	2.0E-4	1.02-1.22
Keum 2015	WG per 5kg increase	Pancreatic CA inc	C25	2	324 / 162,040	1.02 (0.91-1.16)	1.05 (0.87-1.26)	0.97 (0.84-1.12)	0.69	0.63	NA
Keum 2015	WG: highest vs. lowest category	Pancreatic CA inc	C25	4	744 / 327,446	1.04 (0.85-1.26)	1.04 (0.85-1.26)	1.07 (0.77-1.48)	0.71	0.71	0.68-1.59
Aune 2012	WHR per 0.1 units	Pancreatic CA inc	C25	4	1,438 / 1,109,945	1.20 (1.10-1.30)	1.20 (1.09-1.31)	1.32 (1.12-1.56)	5.0E-5	9.5E-5	0.96-1.50
Aune 2012	BMI per 5kg/m^2 increase	Pancreatic CA mort	C25	7	8,921 / 2,568,390	1.08 (1.03-1.13)	1.16 (0.99-1.36)	1.06 (1.01-1.11)	0.001	0.07	0.76-1.77
Yang 2013	BMI, 25+ vs. <25	Lung CA inc	C34	20	13,116 / 7,854,110	0.86 (0.84-0.89)	0.79 (0.73-0.85)	0.97 (0.93-1.01)	1.4E-28	9.9E-10	0.59-1.04
Renehan 2008	BMI per 5kg/m^2 increase	Lung CA inc	C34	15	12,591 / 2,833,306	0.82 (0.80-0.85)	0.77 (0.72-0.83)	0.86 (0.82-0.90)	<1E-100	8.0E-13	0.61-0.97
Renehan 2008	BMI per 5kg/m^2 increase	Lung CA inc, men	C34	10	7,569 / 1,387,620	0.82 (0.79-0.85)	0.79 (0.73-0.85)	0.84 (0.79-0.88)	3.3E-24	1.7E-10	0.65-0.95
Renehan 2008	BMI per 5kg/m^2 increase	Lung CA inc, women	C34	5	5,022 / 1,445,686	0.83 (0.80-0.87)	0.76 (0.61-0.94)	0.86 (0.82-0.90)	2.2E-17	0.01	0.36-1.63
Renehan 2008	BMI per 5kg/m^2 increase	Lung CA inc, smokers	C34	4	6,185 / 475,940	0.82 (0.79-0.86)	0.77 (0.69-0.86)	0.82 (0.77-0.87)	4.0E-20	7.8E-6	0.48-1.24

Renehan 2008	BMI per 5kg/m^2 increase	Lung CA inc, non-smokers	C34	4	500 / 1,157,223	0.90 (0.79-1.02)	0.89 (0.72-1.09)	0.91 (0.77-1.07)	0.09	0.26	0.40-1.97
Yang 2015	BS; surgery vs. no surgery	Melanoma inc	C43	2	75 / 22,819	0.75 (0.43-1.31)	0.75 (0.43-1.31)	0.84 (0.46-1.53)	0.32	0.32	NA
Sergentanis 2013	BMI 25-29.99 vs. < 25	Melanoma inc	C43	13	6,061 / 5,304,374	1.07 (1.00-1.14)	1.08 (0.94-1.24)	1.05 (0.95-1.16)	0.04	0.30	0.73-1.59
Sergentanis 2013	BMI 30+ vs. < 25	Melanoma inc	C43	13	6,361 / 3,231,952	1.09 (1.01-1.18)	1.07 (0.89-1.30)	0.94 (0.83-1.07)	0.02	0.46	0.61-1.88
Sergentanis 2013	BMI, 25+ vs. <25, ASE	Melanoma inc	C43	7	1,629 / 940,536	0.85 (0.69-1.05)	0.86 (0.67-1.10)	0.99 (0.65-1.50)	0.13	0.22	0.51-1.46
Renehan 2008	BMI per 5kg/m^2 increase	Melanoma Inc	C43	11	13,795 / 5,694,985	1.02 (0.99-1.05)	1.07 (0.97-1.17)	0.97 (0.91-1.00)	0.22	0.17	0.81-1.41
Sergentanis 2013	BMI 25-29.99 vs. < 25	Melanoma inc, men	C43	6	1,736 / 2,208,423	1.29 (1.15-1.45)	1.41 (1.09-1.83)	1.27 (1.12-1.45)	2.1E-5	9.6E-3	0.75-2.64
Sergentanis 2013	BMI 30+ vs. < 25	Melanoma inc, men	C43	7	38,39 / 1,399,090	1.30 (1.17-1.45)	1.31 (1.10-1.57)	1.29 (1.14-1.48)	1.8E-6	3.1E-3	0.89-1.93
Sergentanis 2013	BMI, 25+ vs. <25, ASE	Melanoma inc, men	C43	2	414 / 137,176	1.74 (0.85-3.56)	1.74 (0.85-3.56)	2.10 (0.79-5.56)	0.13	0.13	NA
Renehan 2008	BMI per 5kg/m^2 increase	Melanoma inc, men	C43	6	6,647 / 2808095	1.17 (1.11-1.24)	1.17 (1.05-1.30)	1.16 (1.08-1.26)	2.9E-08	0.005	0.89-1.52
Sergentanis 2013	BMI 25-29.99 vs. < 25	Melanoma inc, women	C43	7	4,325 / 3,095,951	0.99 (0.92-1.07)	0.97 (0.87-1.08)	1.05 (0.95-1.16)	0.76	0.54	0.77-1.21
Sergentanis 2013	BMI 30+ vs. < 25	Melanoma inc, women	C43	6	2,522 / 1,832,862	0.91 (0.81-1.02)	0.87 (0.70-1.08)	0.94 (0.83-1.07)	0.09	0.21	0.50-1.50
Sergentanis 2013	BMI, 25+ vs. <25, ASE	Melanoma inc, women	C43	5	1,215 / 803,360	0.80 (0.65-0.99)	0.80 (0.65-0.99)	0.99 (0.65-1.50)	0.04	0.04	0.56-1.13
Renehan 2008	BMI per 5kg/m^2 increase	Melanoma inc, women	C43	5	7,148 / 2,886,890	0.96 (0.93-1.00)	0.96 (0.93-1.00)	0.97 (0.91-1.00)	0.03	0.03	0.90-1.02
Yang 2015	BS; surgery vs. no surgery	Breast CA inc	C50	2	554 / 22,819	0.66 (0.51-0.87)	0.42 (0.07-2.31)	0.98 (0.72-1.32)	0.003	0.32	NA
Suzuki 2009	Obesity±±, high vs. low**	Breast CA inc, ER- PR-, PoMP	C50	4	3,220 / 142,655	0.91 (0.66-1.26)	0.89 (0.53-1.51)	0.75 (0.43-1.31)	0.56	0.68	0.11-7.57
Suzuki 2009	Obesity±±, high vs. low**	Breast CA inc, ER- PR+, PoMP	C50	2	2,127 / 88,952	2.03 (1.05-3.94)	2.03 (1.04-3.95)	1.46 (0.58-3.68)	0.04	0.04	NA
Suzuki 2009	Obesity±±, high vs. low**	Breast CA inc, ER+ PR-, PoMP	C50	2	2,127 / 88,952	0.65 (0.42-0.95)	0.64 (0.42-0.97)	0.76 (0.49-1.17)	0.02	0.04	NA
Suzuki 2009	Obesity±±, high vs. low**	Breast CA inc, ER+ PR+, PoMP	C50	4	3,220 / 142,655	1.68 (1.47-1.91)	1.74 (1.34-2.25)	1.38 (1.12-1.71)	1.3E-14	3.1E-5	0.60-5.06
Renehan 2008	BMI per 5kg/m^2 increase	Breast CA inc, PoMP	C50	29	27,700 / 2,318,992	1.14 (1.12-1.16)	1.13 (1.09-1.17)	1.18 (1.15-1.22)	<1E-100	4.1E-10	0.97-1.31
Cheraghi 2012	BMI 25-29.99 vs. < 25	Breast CA inc, PoMP	C50	8	13,878 / 1,008,448	1.11 (1.07-1.15)	1.12 (1.06-1.18)	1.06 (0.99-1.13)	8.9E-10	5.6E-5	0.96-1.30
Cheraghi 2012	BMI 30+ vs. < 25	Breast CA inc, PoMP	C50	8	11,882 / 873,142	1.14 (1.10-1.19)	1.16 (1.08-1.25)	1.19 (1.10-1.27)	6.1E-12	8.1E-5	0.93-1.44
Harvie 2003	WC: lowest vs. highest category	Breast CA inc, PoMP	C50	4	948 / 38,402	0.69 (0.60-0.79)	0.75 (0.55-1.04)	0.59 (0.49-0.70)	6.3E-8	0.08	0.20-2.84
Keum 2015	WG per 5kg increase	Breast CA inc, PoMP, HRT -	C50	7	10,283 / 342,249	1.11 (1.09-1.13)	1.11 (1.09-1.13)	1.14 (1.11-1.18)	1.5E-31	5.7E-23	1.07-1.15

Keum 2015	WG per 5kg increase	Breast CA inc, PoMP, HRT +	C50	4	4,720 / 100,039	1.01 (0.99-1.02)	1.01 (0.99-1.02)	1.01 (0.98-1.03)	0.52	0.52	0.97-1.05
Keum 2015	WG: highest vs. lowest category	Breast CA inc, PoMP HRT -	C50	7	10,283 / 342,249	1.75 (1.53-2.00)	1.75 (1.53-2.00)	1.98 (1.55-2.53)	7.8E-17	7.8E-17	1.47-2.08
Connolly 2002	WHR: highest vs lowest category	Breast CA inc, PoMP	C50	4	2,716 / 89,369	1.22 (0.99-1.50)	1.24 (0.92-1.67)	1.22 (0.96-1.55)	0.06	0.16	0.48-3.18
Renehan 2008	BMI per 5kg/m^2 increase	Breast CA inc, PrMP	C50	20	20,820 / 2,200,787	0.91 (0.89-0.94)	0.92 (0.88-0.96)	0.91 (0.86-0.97)	2.8E-9	4.6E-4	0.81-1.05
Cheraghi 2012	BMI 25-29.99 vs. < 25	Breast CA inc, PrMP	C50	4	1,316 / 312,368	1.03 (0.91-1.17)	1.01 (0.77-1.31)	0.94 (0.80-1.11)	0.60	0.96	0.33-3.08
Cheraghi 2012	BMI 30+ vs. < 25	Breast CA inc, PrMP	C50	4	1,146 / 278,307	0.91 (0.78-1.07)	0.91 (0.70-1.18)	0.90 (0.75-1.08)	0.26	0.48	0.37-2.25
Harvie 2003	WC: lowest vs. highest category	Breast CA inc, PrMP	C50	2	168 / 7,903	0.77 (0.56-1.05)	0.78 (0.43-1.43)	0.58 (0.38-0.88)	0.09	0.42	NA
Keum 2015	WG per 5kg increase	Breast CA inc, PrMP	C50	3	3,872 / 167,510	0.98 (0.95-1.01)	0.99 (0.95-1.03)	0.96 (0.92-1.00)	0.28	0.51	0.67-1.46
Keum 2015	WG: highest vs. lowest category	Breast CA inc, PrMP	C50	4	4,756 / 171,927	1.00 (0.84-1.20)	0.99 (0.81-1.21)	1.17 (0.90-1.52)	0.97	0.94	0.56-1.75
Amadou 2013	WHR per 0.1 unit increase	Breast CA inc, PrMP	C50	3	1,245 / 136,148	1.02 (0.97-1.07)	1.02 (0.97-1.07)	1.03 (0.97-1.10)	0.48	0.48	0.74-1.39
Connolly 2002	WHR: highest vs lowest category	Breast CA inc, PrMP	C50	3	453 / 56,455	1.29 (0.96-1.75)	1.32 (0.91-1.92	0.96 (0.60-1.54)	0.09	0.15	0.04-41.43
Poorolajal 2015	BMI 25-29.99 vs. < 25	Cervical CA inc	C53	2	1,453 / 5,318,484	1.10 (1.03-1.17)	1.10 (1.03-1.17)	1.10 (1.03-1.17)	4.3E-3	4.3E-3	NA
Poorolajal 2015	BMI 30+ vs. < 25	Cervical CA inc	C53	2	1,453 / 5,318,484	1.20 (1.05-1.36)	1.09 (0.70-1.68)	1.21 (1.06-1.37)	5.8E-3	0.71	NA
Upala 2015	BS; surgery vs. no surgery	Endometrial CA inc	C54.1	3	12,288 / 958,988	0.46 (0.41-0.52)	0.39 (0.19-0.79)	0.48 (0.43-0.55)	1.45E-36	0.009	0.0-1314.2
Zhang 2014	BMI 25-29.99 vs. < 25	Endometrial CA inc	C54.1	7	4,548 / 1,489,424	1.70 (1.59-1.81)	1.60 (1.10-2.33)	1.79 (1.65-1.95)	<1E-100	0.01	0.42-6.09
Zhang 2014	BMI 30+ vs. < 25	Endometrial CA inc	C54.1	6	4,327 / 1,485,506	2.89 (2.69-3.11)	3.10 (2.63-3.65)	2.73 (2.48-2.99)	<1E-100	<1E-100	1.92-5.00
Aune 2015	BMI iya per 5kg/m^2 increase	Endometrial CA inc	C54.1	9	4,345 / 631,915	1.41 (1.33-1.49)	1.45 (1.28-1.64)	1.23 (1.11-1.35)	1.0E-33	1.9E-9	0.98-2.15
Aune 2015	BMI per 5kg/m^2 increase	Endometrial CA inc	C54.1	28	22,320 / 6,445,255	1.57 (1.55-1.60)	1.54 (1.47-1.61)	1.65 (1.60-1.71)	<1E-100	<1E-100	1.26-1.89
Aune 2015	HC per 10cm increase	Endometrial CA inc	C54.1	2	831 / 255,650	1.29 (1.19-1.41)	1.29 (1.19-1.41)	1.32 (1.20-1.45)	2.9E-09	2.9E-09	NA
Aune 2015	WC per 10 cm increase	Endometrial CA inc	C54.1	4	1,524 / 315,770	1.29 (1.23-1.35)	1.27 (1.17-1.39)	1.28 (1.19-1.37)	1.8E-27	7.3E-08	0.88-1.85
Aune 2015	WG per 5kg increase	Endometrial CA inc	C54.1	7	2,806 / 460,901	1.16 (1.13-1.19)	1.16 (1.12-1.20)	1.17 (1.12-1.22)	8.8E-37	3.7E-18	1.06-1.27
Aune 2015	Weight per 5kg increase	Endometrial CA inc	C54.1	7	1,778 / 342,382	1.15 (1.13-1.18)	1.17 (1.13-1.22)	1.11 (1.08-1.15)	<1E-100	7.7E-15	1.04-1.31
Aune 2015	WHR per 0.1 unit increase	Endometrial CA inc	C54.1	5	2,447 / 394,340	1.21 (1.13-1.29)	1.21 (1.13-1.29)	1.33 (1.18-1.51)	1.0E-08	1.0E-08	1.09-1.34
Crosbie 2010	BMI per 5kg/m^2 increase	Endometrial CA inc: PoMP	C54.1	6	10,075 / 2,558,935	1.59 (1.54-1.65)	1.60 (1.40-1.83)	1.51 (1.45-1.58)	<1E-100	1.4E-11	1.01-2.53

Crosbie 2010	BMI per 5kg/m^2 increase	Endometrial CA inc: PoMP HRT +	C54.1	6	791 / 679,271	1.18 (1.07-1.31)	1.18 (1.07-1.31)	1.25 (1.05-1.47)	1.7E-3	1.7E-3	1.02-1.37
Crosbie 2010	BMI per 5kg/m^2 increase	Endometrial CA inc: PoMP HRT -	C54.1	3	699 / 360,326	1.84 (1.68-2.02)	1.90 (1.56-2.30)	1.61 (1.41-1.85)	<1E-100	9.2E-11	0.19-18.56
Keum 2015	WG per 5kg increase	Endometrial CA inc: PoMP, HRT +	C54.1	2	334 / 35,333	1.09 (1.02-1.17)	1.09 (1.02-1.17)	1.09 (1.00-1.18)	0.01	0.01	NA
Keum 2015	WG per 5kg increase	Endometrial CA inc: PoMP, HRT -	C54.1	2	285 / 33,340	1.38 (1.28-1.49)	1.38 (1.28-1.49)	1.36 (1.25-1.46)	3.6E-17	3.6E-17	NA
Crosbie 2010	BMI per 5kg/m^2 increase	Endometrial CA inc: PrMP	C54.1	6	5,981 / 2,558,935	1.52 (1.48-1.57)	1.49 (1.39-1.61)	1.53 (1.48-1.58)	<1E-100	3.1E-27	1.27-1.76
Crosbie 2010	BMI per 5kg/m^2 increase	Endometrial CA inc: Type I	C54.1	3	7,125 / 1,102,927	1.60 (1.55-1.64)	1.75 (1.51-2.03)	1.58 (1.53-1.62)	<1E-100	1.8E-13	0.30-10.24
Crosbie 2010	BMI per 5kg/m^2 increase	Endometrial CA inc: Type II	C54.1	3	1,059 / 1,102,927	1.44 (1.35-1.54)	1.59 (1.29-1.78)	1.35 (1.25-1.46)	2.2E-29	5.7E-7	0.24-9.67
Aune 2015	BMI per 5kg/m^2 increase	Endometrial CA mort	C54.1	3	962 / 1,781,648	1.45 (1.34-1.59)	1.46 (1.29-1.65)	1.42 (1.33-1.63)	1.3E-17	1.7E-09	0.48-4.48
Aune 2015	BMI iya per 5kg/m^2 increase	Ovarian CA inc	C56	6	9,452 / 11,085,425	1.12 (1.05-1.19)	1.12 (1.05-1.19)	1.16 (1.04-1.29)	3.8E-4	3.8E-4	1.03-1.23
Aune 2015	BMI per 5kg/m^2 increase	Ovarian CA inc	C56	24	17,734 / 16,343,135	1.06 (1.03-1.08)	1.08 (1.04-1.12)	0.97 (0.93-1.01)	2.1E-7	8.6E-5	0.96-1.21
Aune 2015	HC per 10cm	Ovarian CA inc	C56	4	1,625 / 479,844	1.08 (1.00-1.16)	1.04 (0.82-1.31)	1.12 (1.03-1.22)	0.06	0.78	0.39-2.78
Aune 2015	WC per 10 cm increase	Ovarian CA inc	C56	6	1,879 / 530,246	1.06 (1.00-1.12)	1.06 (1.00-1.12)	1.04 (0.96-1.13)	0.05	0.05	0.98-1.15
Aune 2015	WG per 5kg increase	Ovarian CA inc	C56	6	1,443 / 428,458	1.01 (0.98-1.05)	1.02 (0.96-1.09)	1.02 (0.97-1.07)	0.43	0.50	0.84-1.25
Aune 2015	Weight per 5kg increase	Ovarian CA inc	C56	4	1,281 / 405,655	1.03 (1.01-1.05)	1.03 (1.01-1.05)	1.02 (1.00-1.05)	8.0E-4	0.001	0.98-1.08
Aune 2015	WHR per 0.1 unit increase	Ovarian CA inc	C56	5	1,329 / 417,558	1.00 (0.93-1.07)	1.00 (0.93-1.07)	0.96 (0.88-1.05)	0.91	0.92	0.89-1.12
Keum 2015	WG per 5kg increase	Ovarian CA inc: PoMP, HRT-	C54.1	2	217 / 23,984	1.13 (1.03-1.24)	1.13 (1.03-1.24)	1.16 (1.03-1.31)	8.5E-3	8.5E-3	NA
Poorolajal 2014	BMI 25-29.99 vs. < 25	Ovarian CA inc	C56	13	8,713 / 25,513,140 pyrs	1.15 (1.10-1.20)	1.08 (0.97-1.19)	1.19 (1.13-1.25)	2.9E-10	0.17	0.81-1.42
Poorolajal 2014	BMI 30+ vs. < 25	Ovarian CA inc	C56	13	6,947 / 20,560,388 pyrs	1.27 (1.19-1.34)	1.27 (1.17-1.38)	1.27 (1.19-1.36)	2.8E-16	2.6E-8	1.09-1.47
Poorolajal 2014	BMI 25-29.99 vs. < 25	Ovarian CA inc, PoMP	C56	5	1,318 / 2,210,807 pyrs	1.02 (0.91-1.14)	1.03 (0.87-1.21)	0.88 (0.73-1.06)	0.74	0.74	0.62-1.70
Poorolajal 2014	BMI 30+ vs. < 25	Ovarian CA inc, PoMP	C56	5	1,116 / 1,858,520 pyrs	1.21 (1.06-1.37)	1.23 (1.03-1.47)	1.02 (0.82-1.26)	3.6E-3	0.03	0.72-2.09
Poorolajal 2014	BMI 25-29.99 vs. < 25	Ovarian CA inc, PrMP	C56	3	317 / 1,940,794 pyrs	1.21 (0.96-1.53)	1.21 (0.96-1.53)	1.34 (1.00-1.80)	0.11	0.11	0.27-5.49
Poorolajal 2014	BMI 30+ vs. < 25	Ovarian CA inc, PrMP	C56	3	284 / 1,758,718 pyrs	1.57 (1.20-2.06)	1.57 (1.20-2.06)	1.56 (1.14-2.16)	9.7E-4	9.7E-4	0.27-9.02
Zhang 2015	BMI obese vs. normal weight**	Prostate CA inc	C61	14	73,851 / 2,342,066	1.02 (0.99-1.05)	1.00 (0.95-1.06)	1.09 (1.04-1.15)	0.23	0.92	0.85-1.18
Renehan 2008	BMI per 5kg/m^2 increase	Prostate CA inc	C61	26	69,740 / 3,027,773	1.03 (1.02-1.04)	1.03 (0.99-1.06)	1.06 (1.04-1.07)	1.2E-7	0.10	0.91-1.16

MacInnis 2006	WC per 10 cm increase	Prostate CA inc	C61	4	1,936 / 56,699	1.03 (0.97-1.09)	1.03 (0.97-1.09)	1.06 (0.97-1.16)	0.34	0.34	0.90-1.17
Keum 2015	WG per 5kg increase	Prostate CA inc	C61	4	6,882 / 102,109	0.98 (0.96-1.01)	0.98 (0.94-1.02)	1.01 (0.98-1.05)	0.15	0.23	0.84-1.14
Keum 2015	WG: highest vs. lowest category	Prostate CA inc	C61	8	19,377 / 426,104	0.99 (0.93-1.04)	0.98 (0.91-1.06)	1.03 (0.94-1.12)	0.61	0.59	0.83-1.16
Discaccati 2012	BMI per 5kg/m^2 increase	Prostate CA inc, advanced	C61	13	7,067 / 1,080,790	1.07 (1.02-1.11)	1.09 (1.02-1.16)	1.00 (0.94-1.08)	0.003	0.01	0.92-1.29
Discaccati 2012	BMI per 5kg/m^2 increase	Prostate CA inc, localized	C61	12	19,130 / 1,033,009	0.95 (0.93-0.97)	0.94 (0.91-0.97)	0.96 (0.93-0.98)	1.3E-06	4.6E-4	0.88-1.01
Keum 2015	WG per 5kg increase	Prostate CA inc, advanced	C61	4	1,094 / 101,692	1.04 (0.99-1.09)	1.04 (0.99-1.09)	1.06 (1.00-1.12)	0.12	0.12	0.93-1.16
Keum 2015	WG per 5kg increase	Prostate CA inc, high screening rate	C61	2	4,894 / 67,335	0.95 (0.92-0.99)	0.95 (0.92-0.99)	0.96 (0.92-1.00)	7.0E-3	7.0E-3	NA
Keum 2015	WG per 5kg increase	Prostate CA inc, localised	C61	4	5,404 / 101,742	0.97 (0.94-1.00)	0.96 (0.92-1.00)	0.99 (0.95-1.03)	0.04	0.08	0.83-1.12
Keum 2015	WG per 5kg increase	Prostate CA inc, low screening rate	C61	2	1,898 / 34,774	1.01 (0.97-1.05)	1.01 (0.97-1.05)	1.01 (0.96-1.05)	0.65	0.65	NA
Zhang 2015	BMI obese vs. normal weight**	Prostate CA mort	C61	10	14,179 / 2,339,669	1.24 (1.15-1.33)	1.24 (1.15-1.33)	1.20 (1.06-1.36)	1.8E-08	1.8E-08	1.13-1.35
Cao 2011	BMI per 5kg/m^2 increase	Prostate CA mort	C61	6	6,817 / 1,263,483	1.10 (1.06-1.13)	1.15 (1.06-1.25)	1.08 (1.04-1.12)	1.8E-08	8.3E-4	0.92-1.44
Yang 2015	BS; surgery vs. no surgery	Kidney CA inc	C64	2	30 / 22,819	1.13 (0.52-2.45)	1.13 (0.52-2.45)	1.21 (0.54-2.71)	0.76	0.76	NA
Wang 2014	BMI 25-29.99 vs. < 25	Kidney CA inc	C64	43	30,501 / 18,988,116	1.28 (1.24-1.33)	1.35 (1.27-1.43)	1.18 (1.11-1.26)	<1E-100	5.0E-22	1.09-1.66
Wang 2014	BMI 30+ vs. < 25	Kidney CA inc	C64	31	29,979 / 15,477,631	1.77 (1.68-1.87)	1.79 (1.64-1.95)	1.85 (1.66-2.06)	<1E-100	3.4E-38	1.31-2.43
Renehan 2008	BMI per 5kg/m^2 increase	Kidney CA inc	C64	19	17,778 / 8,198,668	1.27 (1.24-1.30)	1.30 (1.23-1.36)	1.19 (1.13-1.24)	<1E-100	9.8E-25	1.12-1.50
Keum 2015	WG: highest vs. lowest category	Kidney CA inc	C64	3	1,780 / 781,293	1.42 (1.13-1.78)	1.42 (1.11-1.81)	1.20 (0.90-1.70)	0.002	0.005	0.23-8.71
Renehan 2008	BMI per 5kg/m^2 increase	Kidney CA inc, men	C64	9	8,983 / 3,945,950	1.22 (1.17-1.27)	1.24 (1.17-1.32)	1.19 (1.13-1.24)	1.4E-23	5.9E-12	1.10-1.41
Renehan 2008	BMI per 5kg/m^2 increase	Kidney CA inc, women	C64	10	8,795 / 4,252,718	1.32 (1.27-1.37)	1.33 (1.25-1.42)	1.35 (1.28-1.42)	<1E-100	7.2E-18	1.13-1.57
Sun 2015	BMI 25-29.99 vs. < 25	Bladder CA inc	C67	26	28,745 / 12,562,510	1.05 (1.02-1.09)	1.07 (1.01-1.14)	1.05 (1.00-1.10)	0.001	0.03	0.90-1.27
Sun 2015	BMI 30+ vs. < 25	Bladder CA inc	C67	26	73,491 / 26,538,464	1.10 (1.06-1.14)	1.09 (1.04-1.15)	1.13 (1.06-1.20)	6.2E-07	9.0E-4	0.98-1.21
Sun 2015	BMI per 5kg/m^2 increase	Bladder CA inc	C67	8	11,560 / 6,097,258	1.04 (1.01-1.07)	1.04 (1.00-1.09)	1.03 (1.00-1.06)	0.002	0.07	0.94-1.15
Shao 2014	BMI 25-29.99 vs. < 25	Meningeoma inc	C70	4	799 / 1,995,708	1.11 (0.94-1.30)	1.11 (0.94-1.30)	1.01 (0.79-1.29)	0.22	0.22	0.77-1.58
Shao 2014	BMI 30+ vs. < 25	Meningeoma inc	C70	4	799 / 1,995,708	1.55 (1.28-1.86)	1.55 (1.28-1.86)	1.40 (1.08-1.87)	4.4E-6	4.4E-6	1.03-2.33
Ma 2015	BMI 30+ vs. < 25	Thyroid CA inc	C73	24	16,117 / 22,270,022	1.22 (1.18-1.27)	1.29 (1.20-1.37)	1.17 (1.11-1.23)	8.8E-23	5.6E-14	1.10-1.50

Renehan 2008	BMI per 5kg/m^2 increase	Thyroid CA inc	C73	7	6,716 / 5,316,380	1.15 (1.09-1.21)	1.23 (1.10-1.39)	1.12 (1.05-1.19)	8.50E-08	4.3E-4	0.89-1.70
Keum 2015	WG: highest vs. lowest category	Thyroid CA inc	C73	4	769 / 744,703	1.06 (0.82-1.38)	1.06 (0.82-1.38)	0.88 (0.57-1.38)	0.64	0.64	0.60-1.88
Renehan 2008	BMI per 5kg/m^2 increase	Thyroid CA inc, men	C73	4	3,526 / 3,157,142	1.18 (1.08-1.29)	1.32 (1.04-1.69)	1.09 (0.98-1.22)	3.2E-4	0.02	0.46-3.84
Renehan 2008	BMI per 5kg/m^2 increase	Thyroid CA inc, women	C73	3	3,190 / 2,159,238	1.13 (1.07-1.20)	1.14 (1.06-1.22)	1.12 (1.05-1.19)	5.3E-5	4.0E-4	0.67-1.93
Larsson 2011	BMI 25-29.99 vs. < 25	Hodgkin's lymphoma inc	C81	7	2,815 / 5,749,783	0.96 (0.86-1.07)	0.97 (0.85-1.12)	0.89 (0.76-1.04)	0.45	0.72	0.74-1.28
Larsson 2011	BMI 30+ vs. < 25	Hodgkin's lymphoma inc	C81	4	2,716 / 4,828,366	1.43 (1.19-1.71)	1.41 (1.14-1.75)	1.66 (1.28-2.14)	1.1E-4	1.9E-3	0.73-2.72
Yang 2015	BS; surgery vs. no surgery	NHL inc	C82-C85	2	36 / 22,819	0.57 (0.25-1.29)	0.57 (0.25-1.29)	0.59 (0.24-1.42)	0.18	0.18	NA
Larsson 2007	BMI 25-29.99 vs. < 25	NHL inc	C82-C85	15	24,421 / 7,611,421	1.04 (1.00-1.08)	1.06 (0.99-1.12)	1.03 (0.97-1.10)	0.04	0.08	0.93-1.20
Larsson 2007	BMI 30+ vs. < 25	NHL inc	C82-C85	15	24,421 / 7,611,421	1.17 (1.11-1.24)	1.19 (1.04-1.37)	1.09 (1.00-1.19)	8.8E-8	0.01	0.78-1.80
Larsson 2011	BMI per 5kg/m^2 increase	NHL inc	C82-C85	23	29,064 / 9,056,494	1.07 (1.04-1.09)	1.07 (1.04-1.10)	1.04 (0.99-1.09)	2.1E-7	1.9E-6	1.02-1.12
Larsson 2011	BMI per 5kg/m^2 increase	NHL mort	C82-C85	7	5,920 / 3,511,893	1.16 (1.10-1.22)	1.14 (1.04-1.25)	1.18 (1.09-1.29)	1.4E-8	5.3E-3	0.90-1.45
Castillo 2014	BMI 25-29.99 vs. < 25	Diff .large B-cell lymphoma inc	C83.3	10	2,445 / 2,348,783	1.13 (1.02-1.25)	1.13 (1.02-1.25)	1.29 (1.04-1.61)	0.02	0.02	1.00-1.27
Castillo 2014	BMI 30+ vs. < 25	Diff. large B-cell lymphoma inc	C83.3	12	3,093 / 2,981,742	1.27 (1.13-1.43)	1.25 (1.08-1.44)	1.64 (1.25-2.16)	7.9E-5	2.6E-3	0.89-1.74
Yang 2015	BS; surgery vs. no surgery	Multiple myeloma inc	C90.0	2	13 / 22,819	0.61 (0.14-2.63)	0.61 (0.14-2.63)	0.72 (0.13-3.91)	0.50	0.50	NA
Wallin 2012	BMI 25-29.99 vs. < 25	Multiple myelomai nc	C90.0	20	15,990 / 8,510,803	1.12 (1.07-1.18)	1.12 (1.07-1.18)	1.14 (1.06-1.22)	3.4E-7	3.4E-7	1.07-1.18
Wallin 2012	BMI 30+ vs. < 25	Multiple myeloma inc	C90.0	19	15,887 / 7,729,520	1.24 (1.15-1.33)	1.21 (1.08-1.35)	1.31 (1.16-1.48)	4.5E-9	1.0E-3	0.90-1.62
Renehan 2008	BMI per 5kg/m^2 increase	Multiple myeloma inc	C90.0	13	14,634 / 7,429,478	1.12 (1.09-1.15)	1.12 (1.09-1.15)	1.11 (1.07-1.15)	2.8E-15	2.8E-15	1.08-1.15
Renehan 2008	BMI per 5kg/m^2 increase	Multiple myeloma inc, men	C90.0	7	7,451 / 3,821,528	1.12 (1.07-1.17)	1.12 (1.06-1.18)	1.13 (1.07-1.19)	2.0E-06	1.1E-4	1.02-1.22
Renehan 2008	BMI per 5kg/m^2 increase	Multiple myeloma inc, women	C90.0	6	7,183 / 3,607,950	1.11 (1.08-1.15)	1.11 (1.08-1.15)	1.11 (1.07-1.15)	2.8E-10	2.8E-10	1.06-1.17
Wallin 2012	BMI 25-29.99 vs. < 25	Multiple myeloma mort	C90.0	8	3,337 / 3,714,187	1.15 (1.04-1.26)	1.15 (1.04-1.26)	1.18 (1.01-1.39)	5.9E-3	5.9E-3	1.02-1.30
Wallin 2012	BMI 30+ vs. < 25	Multiple myeloma mort	C90.0	7	3,239 / 3,604,489	1.54 (1.35-1.76)	1.54 (1.35-1.76)	1.46 (1.16-1.84)	2.1E-10	2.1E-10	1.29-1.83
Castillo 2012	BMI 30+ vs. < 25	Leukemia inc	C91-C92	25	51,914 / 21,228,740	1.30 (1.24-1.37)	1.26 (1.16-1.37)	1.42 (1.31-1.54)	6.3E-27	1.6E-08	0.98-1.62
Castillo 2012	BMI 30+ vs. < 25	Leukemia mort	C91-C92	8	6,527 / 4,205,316	1.27 (1.14-1.42)	1.28 (1.11-1.49)	1.37 (1.13-1.67)	1.3E-5	7.5E-4	0.93-1.77
Renehan 2008	BMI per 5kgm2 increase	Leukemia inc	C91-C95	8	15,117 / 6,758,260	1.10 (1.06-1.13)	1.12 (1.05-1.18)	1.07 (1.02-1.12)	3.1E-08	2.3E-4	0.96-1.30

Renehan 2008	BMI per 5kg/m^2 increase	Leukemia inc, men	C91-C95	5	7,827 / 3,497,392	1.08 (1.02-1.13)	1.08 (1.02-1.13)	1.09 (1.02-1.17)	0.008	0.008	0.99-1.17
Renehan 2008	BMI per 5kg/m^2 increase	Leukemia inc, women	C91-C95	3	7,290 / 3,260,868	1.11 (1.06-1.16)	1.17 (1.04-1.33)	1.07 (1.02-1.12)	7.4E-07	0.01	0.28-4.93
Larsson 2008	BMI 30+ vs. < 25	Leukaemia inc, ALL	C91	5	588 / 13,337,737	1.65 (1.19-1.29)	1.65 (1.16-2.35)	1.33 (0.82-2.15)	2.9E-3	5.1E-3	0.85-3.23
Larsson 2008	BMI 30+ vs. < 25	Leukaemia inc, CLL	C91	5	6,547 / 13,038,983	1.25 (1.14-1.36)	1.25 (1.11-1.41)	1.30 (1.13-1.49)	1.8E-6	2.3E-4	0.92-1.70
Larsson 2008	BMI 30+ vs. < 25	Leukaemia inc, AML	C92	6	4,804 / 13,375,364	1.37 (1.24-1.51)	1.52 (1.19-1.95)	1.17 (1.00-1.36)	2.1E-10	8.9E-4	0.69-3.38
Larsson 2008	BMI 30+ vs. < 25	Leukaemia inc, CML	C92	5	2,530 / 13,337,737	1.26 (1.09-1.46)	1.26 (1.09-1.46)	1.15 (0.92-1.45)	1.7E-3	1.7E-3	1.00-1.60

Abbreviations: AdCa, adenocarcinoma; ALL, chronic lymphatic leukemia; AML, chronic myeloic leukemia; ASE, adjusted for sunlight exposure; biliary tract system cancer, includes cancers of gallbladder, extrahepatic bile duct and ampulla of Vateri; BMI, Body Mass Index; BMI iya, Body Mass Index in young adulthood; BS, bariatric surgery; CA, cancer; CI, confidence interval; CLL, chronic lymphatic leukemia; CML, chronic myeloic leukemia; ER = estrogen receptor status ; HC, hip circumference; HRT, hormone replacement therapy; inc, incidence; mort, mortality; NA: Not available, due to <3 included studies; NHL, Non-Hodgkin lymphoma; NP: Not Pertinent, because the estimated is larger than the observed, and there is no evidence of excess statistical significance based on the assumption made for the plausible effect size; PoMP, postmenopausal; PR = progesterone receptor status; PrMP, premenopausal; pyrs, person-years; RR, relative risk; SqCa, squamous cell carcinoma; WC, waist circumference; WG, weight gain; WHR, waist-to-hip ratio;

* 10 out of 202 original meta-analyses included less than 2 cohort studies.
‡ Fixed effects refers to summary relative risk (95% CI) using the meta-analysis fixed-effects model.
§ Random effects refers to summary relative risk (95% CI) using the meta-analysis random-effects model.
† Relative risk and 95% confidence interval of largest study (smallest SE) in each meta-analysis.
¶ P value of summary fixed effects estimate.
|| P value of summary random effects estimate.
Prediction intervals are reported only for meta-analyses including at least 3 studies.
** cut-offs in categories varied between included studies
±± BMI, weight gain or weight change, varies between included studies
All statistical tests were two-sided.

Supplementary Table 2: Evaluation of heterogeneity, small study effects and excess significance bias in the 194 meta-analyses investigating the associations between obesity and the risk of cancer development or mort (only cohort studies included).

Author, year	Exposure	Association of obesity or weight change with	ICD-10	Egger's P¶	I^2 (95% CI) P‡	Studies	Observed	Expected§, P-value‖					
								Fixed effects		Random effects		Largest study	
Casagrande 2014	BS inc dens. rate after surg	Overall CA inc	C00-C97	0.20	96 (95-97) <0.01	9	6	4.6	0.51	0.7	<0.01	5.0	0.74
Casagrande 2014	BS; surgery vs. no surgery	Overall CA inc	C00-C97	0.03	94 (88-96) <0.01	4	4	4.0	1.00	4.0	1.00	3.3	1.00
Renehan 2008	BMI per 5kg/m^2 increase	Oesophageal AdCa inc	C15	0.19	0 (0-56) 0.60	8	6	6.0	1.00	6.0	1.00	6.5	NP
Singh 2013	Central adiposity	Oesophageal AdCa inc	C15	NA	0	2	1	1.9	NP	1.9	NP	1.9	NP
Renehan 2008	BMI per 5kg/m^2 increase	Oesophageal AdCa inc, men	C15	0.37	24 (0-72) 0.26	5	4	3.3	0.67	3.2	0.66	3.6	1.00
Renehan 2008	BMI per 5kg/m^2 increase	Oesophageal AdCa inc, wom	C15	0.44	0 (0-73) 0.96	3	2	2.7	NP	2.7	NP	2.7	NP
Renehan 2008	BMI per 5kg/m^2 increase	Oesophageal SqCa inc	C15	0.26	80 (35-90) <0.01	5	5	4.4	1.00	4.6	1.00	3.5	0.33
Renehan 2008	BMI per 5kg/m^2 increase	Oesophageal SqCa inc, men	C15	0.03	44 (0-83) 0.17	3	3	2.0	0.56	2.2	0.57	1.8	0.28
Renehan 2008	BMI per 5kg/m^2 increase	Oesophageal SqCa inc, wom	C15	NA	58	2	2	2.0	1.00	2.0	1.00	2.0	1.00
Turati 2013	BMI 25-29.99 vs. < 25	Gastrc cardia and oesophageal AdCa	C15-C16.0	0.50	0 (0-53) 0.47	10	6	7.9	NP	7.9	NP	6.7	NP
Turati 2013	BMI 30+ vs. < 25	Gastrc cardia and oesophageal AdCa	C15-C16.0	0.29	34 (0-69) 0.15	9	8	8.6	NP	8.6	NP	8.4	NP

Lin 2014	BMI 25-29.99 vs. < 25	Gastric CA inc	C16	0.18	30 (0-64) 0.15	12	2	0.7	0.16	0.6	0.12	1.4	0.64
Lin 2014	BMI 30+ vs. < 25	Gastric CA inc	C16	0.99	7 (0-55) 0.38	11	2	4.0	NP	4.0	NP	8.3	NP
Renehan 2008	BMI per 5kg/m^2 increase	Gastric CA inc	C16	0.28	37 (0-66) 0.08	13	1	1.0	1.00	0.7	0.51	1.4	NP
Renehan 2008	BMI per 5kg/m^2 increase	Gastric CA inc, men	C16	0.64	39 (0-72) 0.12	8	1	0.9	1.00	0.7	0.51	1.0	NP
Renehan 2008	BMI per 5kg/m^2 increase	Gastric CA inc, wom	C16	0.44	41 (0-77) 0.15	5	0	0.3	NP	0.36	NP	0.4	NP
Larsson 2007	BMI per 5kg/m^2 increase	Colon CA inc	C18	<0.01	84 (79-87) <0.01	44	27	13.4	<0.01	23.9	0.37	4.4	<0.01
Larsson 2007	WC per 10cm increase	Colon CA inc	C18	0.06	50 (0-76) 0.05	8	7	4.0	0.07	4.6	0.15	2.4	<0.01
Larsson 2007	WHR per 0.1 unit increase	Colon CA inc	C18	0.16	53 (0-77) 0.04	8	6	4.5	0.48	4.9	0.71	4.2	0.29
Keum 2015	WG per 5kg increase	Colon CA inc, overall	C18	0.88	0 (0-58) 0.46	7	2	0.8	0.14	0.8	0.14	1.3	0.50
Moghaddam 2007	BMI 30+ vs. < 25	Colon CA inc, overall	C18	0.01	76 (65-82) <0.001	28	15	11.9	0.23	21	NP	4.4	<0.01
Larsson 2007	BMI per 5kgm2 increase	Colon CA inc, men	C18	0.30	24 (0-53) 0.15	24	18	16.1	0.52	16.7	0.67	15.4	0.39
Keum 2015	WG per 5kg increase	Colon CA inc, men	C18	0.56	0 (0-68) 0.72	4	2	0.7	0.07	0.7	0.07	0.8	0.12
Moghaddam 2007	BMI 30+ vs. < 25	Colon CA inc, men	C18	0.02	0 (0-47) 0.66	14	9	11.5	NP	11.5	NP	11.1	NP
Larsson 2007	WC per 10cm increase	Colon CA inc, men	C18	0.51	45 (0-78) 0.12	5	4	3.3	0.66	3.5	1.00	1.5	0.03
Larsson 2007	WHR per 0.1 unit increase	Colon CA inc, men	C18	0.32	56 (0-83) 0.08	4	4	3.0	0.57	3.2	0.59	2.1	0.13
Larsson 2007	BMI per 5kg/m^2 increase	Colon CA inc, wom	C18	<0.01	59 (26-74) <0.01	20	9	2.8	<0.01	5.5	0.13	2.1	<0.01
Larsson 2007	WC per 10cm increase	Colon CA inc, wom	C18	0.25	0 (0-73) 0.52	3	3	1.2	0.06	1.2	0.06	1.0	0.04
Larsson 2007	WHR per 0.1 unit increase	Colon CA inc, wom	C18	0.90	30 (0-77) 0.23	4	2	1.8	1.00	1.8	1.00	2.1	NP
Keum 2015	WG per 5kg increase	Colon CA inc, women	C18	0.66	0 (0-73) 0.45	3	0	0.2	NP	0.2	NP	0.2	NP
Moghaddam 2007	BMI 30+ vs. < 25	Colon CA inc, women	C18	0.04	51 (0-72) 0.01	14	6	2.6	0.02	5.9	0.94	2.2	<0.01
Robsham 2013	BMI, Obese vs. normal**	Colon CA inc; distal colon	C18	0.49	44 (0-66) 0.02	20	7	15.5	NP	16.6	NP	1.3	<0.01
Robsham 2013	BMI, Obese vs. normal**	Colon CA inc; distal colon, men	C18	0.64	17 (0-65) 0.30	7	3	6.2	NP	6.3	NP	6.5	NP
Robsham 2013	BMI, Obese vs. normal**	Colon CA inc; distal colon, women	C18	0.51	56 (0-76) 0.009	12	4	8.3	NP	9.4	NP	0.7	<0.01
Robsham 2013	BMI, Obese vs. normal**	Colon CA inc; proximal colon	C18	0.12	33 (0-60) 0.07	20	3	7.9	NP	11.0	NP	1.1	0.053

Robsham 2013	BMI, Obese vs. normal**	Colon CA inc; prox. colon, men	C18	0.44	0 (0-58) 0.46	7	1	4.9	NP	4.9	NP	2.0	NP
Robsham 2013	BMI, Obese vs. normal**	Colon CA inc; prox. colon, women	C18	0.41	25 (0-62) 0.20	12	1	1.9	NP	3.2	NP	0.6	0.64
Yang 2015	BS; surgery vs. no surgery	Colorectal CA inc	C18-C20	0.06	0 (0-64) 0.56	5	0	2.2	NP	2.2	NP	2.0	NP
Ma 2013	BMI, Obese vs. normal**	Colorectal CA inc	C18-C20	0.001	69 (60-75) <0.01	72	31	47.0	NP	62.4	NP	14.2	<0.0 1
Ma 2013	WC, highest vs. lowest category	Colorectal CA inc	C18-C20	0.015	9 (0-48) 0.35	19	13	16.8	NP	16.9	NP	14.5	NP
Schlesinger 2015	WG per 1kg increase / year	Colorectal CA inc	C18-C20	0.72	37 (0-63) 0.07	17	4	12.9	NP	12.4	NP	15.3	NP
Schlesinger 2015	WG: high gain vs. stable weight	Colorectal CA inc	C18-C20	0.83	0 (0-43), 0.50	19	2	12.7	NP	12.7	NP	15.8	NP
Moghaddam 2007	BMI 30+ vs. < 25	Rectal CA inc	C20	0.08	31 (0-58) 0.09	21	3	6.6	NP	9.7	NP	2.9	1.00
Harriss 2009	BMI per 5kg/m^2 increase	Rectal CA inc	C20	0.35	27 (0-52) 0.09	31	4	4.3	NP	5.0	NP	1.7	0.09
Harriss 2009	BMI per 5kg/m^2 increase	Rectal CA inc, men	C20	0.66	6 (0-48) 0.66	17	3	3.5	NP	3.6	NP	3.2	NP
Moghaddam 2007	BMI 30+ vs. < 25	Rectal CA inc, men	C20	0.44	0 (0-51) 0.88	11	2	5.5	NP	5.5	NP	5.3	NP
Harriss 2009	BMI per 5kg/m^2 increase	Rectal CA inc, wom	C20	0.47	0 (0-47) 0.56	14	1	0.9	0.62	0.9	0.62	0.8	0.55
Moghaddam 2007	BMI 30+ vs. < 25	Rectal CA inc, women	C20	0.13	7 (0-56) 0.37	10	1	1.3	NP	1.8	NP	1.0	1.00
Chen 2012	BMI 25-29.99 vs. < 25	Liver CA inc	C22	0.08	61 (19-77) <0.01	14	4	3.4	0.76	5.5	NP	2.6	0.31
Chen 2012	BMI 30+ vs. < 25	Liver CA inc	C22	0.03	68 (44-79) <0.01	19	15	15.2	NP	16.6	NP	10.9	0.07
Wang 2012	BMI per 5kg/m^2 increase	Liver CA inc	C22	0.05	81 (71-87) <0.01	19	10	7.6	0.35	11.3	NP	4.7	0.01
Li 2014	BMI 25-29.99 vs. < 25	Cholangiocarcinoma inc	C22.1, C24	0.22	0 (0-58) 0.87	7	1	4.0	NP	4.0	NP	5.4	NP
Li 2014	BMI 30+ vs. < 25	Cholangiocarcinoma inc	C22.1, C24	0.09	7 (0-64) 0.37	6	2	3.8	NP	3.8	NP	3.2	NP
Larsson 2007	BMI 30+ vs. < 25	Gallbladder CA inc	C23	0.11	14 (0-56) 0.30	13	7	9.8	NP	9.6	NP	10.4	NP
Park 2014	BMI per 5kg/m^2 increase	Biliary tract system CA inc	C23-C24	0.24	25 (0-64) 0.21	10	4	8.5	NP	8.2	NP	9.0	NP
Yang 2015	BS; surgery vs. no surgery	Pancreatic CA inc	C25	NA	57	2	0	0.1	NP	0.1	NP	0.5	NP
Alsamarrai 2014	BMI, 25+ vs. <25	Pancreatic CA inc	C25	0.88	97 (95-97) <0.01	9	3	5.7	NP	4.8	NP	4.1	NP
Aune 2012	BMI per 5kg/m^2 increase	Pancreatic CA inc	C25	0.34	25 (0-55) 0.14	22	4	3.8	0.78	4.0	1.00	3.4	0.77
Aune 2012	WC per 10cm	Pancreatic CA inc	C25	0.11	0 (0-64) 0.74	5	2	0.8	0.19	0.8	0.19	0.5	0.09

Keum 2015	WG per 5kg increase	Pancreatic CA inc	C25	NA	50	2	0	0.1	NP	0.1	NP	0.1	NP
Keum 2015	WG: highest vs. lowest category	Pancreatic CA inc	C25	0.42	0 (0-68) 0.48	4	0	0.2	NP	0.2	NP	0.3	NP
Aune 2012	WHR per 0.1 unit increase	Pancreatic CA inc	C25	0.54	7 (0-70) 0.36	4	2	1.8	1.00	1.8	1.00	3.0	NP
Aune 2012	BMI per 5kg/m^2 increase	Pancreatic CA mort	C25	0.44	54 (0-79) 0.04	7	3	1.7	0.37	2.9	1.00	1.4	0.14
Yang 2013	BMI, 25+ vs. <25	Lung CA inc	C34	0.02	80 (69-86) <0.01	20	10	8.4	0.50	13.6	NP	1.4	<0.0 1
Renehan 2008	BMI per 5kg/m^2 increase	Lung CA inc	C34	0.05	71 (47-82) <0.01	15	10	8.9	0.61	11.3	NP	7.1	0.19
Renehan 2008	BMI per 5kg/m^2 increase	Lung CA inc, men	C34	0.07	53 (0-75) 0.02	10	7	5.6	0.53	6.6	1.00	5.0	0.23
Renehan 2008	BMI per 5kg/m^2 increase	Lung CA inc, wom	C34	0.46	86 (66-92) <0.01	5	3	3.3	NP	4.4	NP	3.0	1.00
Renehan 2008	BMI per 5kg/m^2 increase	Lung CA inc, smokers	C34	0.12	78 (1-90) 0.003	4	4	2.9	0.58	3.2	1.00	2.9	0.58
Renehan 2008	BMI per 5kg/m^2 increase	Lung CA inc, non-smokers	C34	0.83	54 (0-83) 0.09	4	1	0.4	0.35	0.5	0.39	0.4	0.31
Yang 2015	BS; surgery vs. no surgery	Melanoma inc	C43	NA	0	2	0	0.3	NP	0.3	NP	0.2	NP
Sergentanis 2013	BMI 25-29.99 vs. < 25	Melanoma inc	C43	0.48	59 (9-76) <0.01	13	3	1.7	0.22	1.9	0.42	1.2	0.11
Sergentanis 2013	BMI 30+ vs. < 25	Melanoma inc	C43	1.00	67 (32-80) <0.01	13	3	2.5	0.72	1.8	0.41	1.5	0.19
Sergentanis 2013	BMI, 25+ vs. <25, ASE	Melanoma inc	C43	0.29	25 (0-68) 0.24	7	1	1.9	NP	1.7	NP	0.4	0.31
Renehan 2008	BMI per 5kg/m^2 increase	Melanoma Inc	C43	0.26	79 (59-87) <0.01	11	4	0.7	<0.01	2.5	0.28	1.0	0.01
Sergentanis 2013	BMI 25-29.99 vs. < 25	Melanoma inc, men	C43	0.16	35 (0-73) 0.17	6	2	2.8	NP	3.6	NP	2.6	NP
Sergentanis 2013	BMI 30+ vs. < 25	Melanoma inc, men	C43	0.89	26 (0-69) 0.23	7	3	4.6	NP	4.7	NP	4.5	NP
Sergentanis 2013	BMI, 25+ vs. <25, ASE	Melanoma inc, men	C43	NA	0	2	0	2.0	NP	2.0	NP	1.9	NP
Renehan 2008	BMI per 5kg/m^2 increase	Melanoma inc, men	C43	0.89	46 (0-77) 0.10	6	3	2.6	1.00	2.5	0.70	2.5	0.69
Sergentanis 2013	BMI 25-29.99 vs. < 25	Melanoma inc, wom	C43	0.43	22 (0-67) 0.26	7	1	0.4	0.32	0.5	0.43	0.7	0.53
Sergentanis 2013	BMI 30+ vs. < 25	Melanoma inc, wom	C43	0.86	37 (0-74) 0.16	6	0	1.1	NP	1.8	NP	0.7	NP
Sergentanis 2013	BMI, 25+ vs. <25, ASE	Melanoma inc, wom	C43	0.12	0 (0-64) 0.48	5	1	2.4	NP	2.4	NP	0.3	0.23
Renehan 2008	BMI per 5kg/m^2 increase	Melanoma inc, wom	C43	0.28	0 (0-64) 0.46	5	1	0.7	0.52	0.7	0.52	0.5	0.40
Yang 2015	BS; surgery vs. no surgery	Breast CA inc	C50	NA	97	2	1	1.9	NP	2.0	NP	0.1	0.10

Suzuki 2009	Obesity±±, high vs. low**	Breast CA inc, ER- PR-, PoMP	C50	0.80	61 (0-85) 0.05	4	0	1.3	NP	1.6	NP	3.9	NP
Suzuki 2009	Obesity±±, high vs. low**	Breast CA inc, ER- PR+, PoMP	C50	NA	61	2	1	2.0	NP	2.0	NP	2.0	NP
Suzuki 2009	Obesity±±, high vs. low**	Breast CA inc, ER+ PR-, PoMP	C50	NA	27	2	1	2.0	NP	2.0	NP	0.1	0.10
Suzuki 2009	Obesity±±, high vs. low**	Breast CA inc, ER+ PR+, PoMP	C50	0.70	69 (0-87) 0.02	4	3	4.0	NP	4.0	NP	4.0	NP
Renehan 2008	BMI per 5kg/m^2 increase	Breast CA inc, PoMP	C50	0.67	61 (38-73) <0.01	29	12	13.3	NP	11.9	1.00	16.6	NP
Harvie 2003	WC: lowest vs. highest category	Breast CA inc, PoMP	C50	0.60	73 (0-88) 0.01	4	1	2.6	NP	2.2	NP	3.1	NP
Keum 2015	WG per 5kg increase	Breast CA inc, PoMP HRT -	C50	0.66	17 (0-65) 0.30	7	5	3.6	0.45	3.5	0.45	4.5	1.00
Keum 2015	WG: highest vs. lowest category	Breast CA inc, PoMP HRT -	C50	0.43	0 (0-58) 0.55	7	5	6.8	NP	6.8	NP	6.9	NP
Connolly 2002	WHR: highest vs lowest category	Breast CA inc, PoMP	C50	0.65	23 (0-75) 0.27	4	1	2.3	NP	2.4	NP	2.3	NP
Keum 2015	WG per 5kg increase	Breast CA inc, PoMP HRT +	C50	0.47	0 (0-68) 0.48	4	0	0.2	NP	0.2	NP	0.2	NP
Cheraghi 2012	BMI 25-29.99 vs. < 25	Breast CA inc, PoMP	C50	0.16	57 (0-78) 0.02	8	4	4.6	NP	5.0	NP	2.1	0.12
Cheraghi 2012	BMI 30+ vs. < 25	Breast CA inc, PoMP	C50	0.37	65 (1-82) 0.005	8	4	5.5	NP	5.9	NP	6.4	NP
Renehan 2008	BMI per 5kg/m^2 increase	Breast CA inc, PrMP	C50	0.28	38 (0-63) 0.05	20	7	6.0	0.63	5.2	0.44	6.3	0.81
Harvie 2003	WC: highest vs. lowest category	Breast CA inc, PrMP	C50	NA	73	2	1	0.5	0.45	0.5	0.41	1.6	NP
Keum 2015	WG per 5kg increase	Breast CA inc, PrMP	C50	0.74	35 (0-81) 0.21	3	0	0.2	NP	0.2	NP	0.4	NP
Keum 2015	WG: highest vs. lowest category	Breast CA inc, PrMP	C50	0.42	17 (0-73) 0.31	4	0	0.2	NP	0.2	NP	3.2	NP
Amadou 2013	WHR per 0.1 unit increase	Breast CA inc, PrMP	C50	0.08	0 (0-73) 0.73	3	0	0.2	NP	0.2	NP	0.2	NP
Connolly 2002	WHR: highest vs lowest category	Breast CA inc, PrMP	C50	0.27	34 (0-81) 0.22	3	1	1.2	NP	1.4	NP	0.2	0.17
Cheraghi 2012	BMI 25-29.99 vs. < 25	Breast CA inc, PrMP	C50	0.79	72 (0-88) 0.01	4	1	0.2	0.12	0.2	0.07	0.4	0.29
Cheraghi 2012	BMI 30+ vs. < 25	Breast CA inc, PrMP	C50	0.56	37 (0-78) 0.19	4	0	0.5	NP	0.6	NP	0.7	NP
Poorolajal 2015	BMI 25-29.99 vs. < 25	Cervical CA inc	C53	NA	0	2	1	0.5	0.47	0.5	0.47	0.6	0.49
Poorolajal 2015	BMI 30+ vs. < 25	Cervical CA inc	C53	NA	36	2	1	1.1	NP	0.5	0.41	1.1	NP
Upala 2015	BS; surgery vs. no surgery	Endometrial CA inc	C54.1	0.74	80 (0-92) <0.01	3	2	2.5	NP	2.7	NP	2.5	NP
Zhang 2014	BMI 25-29.99 vs. < 25	Endometrial CA inc	C54.1	0.71	96 (93-97) <0.01	7	7	6.9	1.00	6.7	1.00	6.9	1.00

Zhang 2014	BMI 30+ vs. < 25	Endometrial CA inc	C54.1	0.24	66 (0-84) 0.01	6	6	6.0	1.00	6.0	1.00	6.0	1.00
Aune 2015	BMI iya per 5kg/m^2 increase	Endometrial CA inc	C54.1	0.41	75 (45-86) <0.01	9	8	7.5	1.00	7.8	1.00	5.2	0.09
Aune 2015	BMI per 5kg/m^2 increase	Endometrial CA inc	C54.1	0.35	81 (73-86) <0.01	28	26	24.4	0.57	24.0	0.42	25.2	1.00
Aune 2015	HC per 10cm increase	Endometrial CA inc	C54.1	NA	0	2	1	1.6	NP	1.6	NP	1.7	NP
Aune 2015	WC per 10cm increase	Endometrial CA inc	C54.1	0.59	70 (0-88) 0.02	4	3	3.0	1.00	2.9	1.00	2.9	1.00
Aune 2015	WG per 5kg increase	Endometrial CA inc	C54.1	0.95	47 (0-76) 0.08	7	6	2.5	0.01	2.5	0.01	2.8	0.02
Aune 2015	Weight per 5kg increase	Endometrial CA inc	C54.1	0.29	62 (0-81) 0.01	7	6	1.7	<0.01	1.9	<0.01	1.0	<0.0 1
Aune 2015	WHR per 0.1 unit increase	Endometrial CA inc	C54.1	0.54	0 (0-64) 0.48	5	3	3.0	1.00	3.0	1.00	4.4	NP
Crosbie 2010	BMI per 5kg/m^2 increase	Endometrial CA inc: PoMP	C54.1	0.88	89 (79-83) <0.001	6	6	5.3	1.00	5.3	1.00	5.0	0.60
Crosbie 2010	BMI per 5kg/m^2 increase	Endometrial CA inc: PoMP HRT +	C54.1	0.77	0 (0-61) 0.87	6	1	1.1	NP	1.1	NP	1.7	NP
Crosbie 2010	BMI per 5kg/m^2 increase	Endometrial CA inc: PoMP HRT-	C54.1	0.005	77 (0-91) 0.01	3	3	3.0	1.00	3.0	1.00	2.9	1.00
Keum 2015	WG per 5kg increase	Endometrial CA inc: PoMP, HRT+	C54.1	NA	0 (.-.) 1.00	2	1	0.2	0.18	0.2	0.18	0.2	0.18
Keum 2015	WG per 5kg increase	Endometrial CA inc: PoMP, HRT-	C54.1	NA	0 (.-.) 0.42	2	2	1.1	0.51	1.1	0.51	1.0	0.50
Crosbie 2010	BMI per 5kg/m^2 increase	Endometrial CA inc: PrMP	C54.1	0.56	20 (0-68) 0.28	6	5	4.1	0.67	4.0	0.67	4.2	0.67
Crosbie 2010	BMI per 5kg/m^2 increase	Endometrial CA inc: Type I	C54.1	0.26	82 (0-92) 0.004	3	3	2.9	1.00	3.0	1.00	2.9	1.00
Crosbie 2010	BMI per 5kg/m^2 increase	Endometrial CA inc: Type II	C54.1	0.52	76 (0-91) 0.02	3	3	1.7	0.27	1.9	0.30	1.5	0.25
Aune 2015	BMI per 5kgm2 increase	Endometrial CA mort	C54.1	0.64	29 (0-80) 0.25	3	2	2.1	NP	2.1	NP	2.0	1.00
Aune 2015	BMI iya per 5kg/m^2 increase	Ovarian CA inc	C56	0.60	0 (0-61) 0.56	6	1	2.0	NP	2.0	NP	2.5	NP
Aune 2015	BMI per 5kg/m^2 increase	Ovarian CA inc	C56	0.07	48 (5-66) <0.01	24	6	3.0	0.11	4.0	0.27	1.8	<0.0 1
Poorolajal 2014	BMI 25-29.99 vs. < 25	Ovarian CA inc	C56	0.16	49 (0-71) 0.02	13	2	3.2	NP	1.9	1.00	4.0	NP
Poorolajal 2014	BMI 30+ vs. < 25	Ovarian CA inc	C56	0.88	12 (0-54) 0.33	13	3	5.2	NP	5.2	NP	5.3	NP
Aune 2015	HC per 10cm increase	Ovarian CA inc	C56	0.77	72 (0-88) 0.01	4	2	0.5	0.09	0.3	0.02	1.0	0.25
Aune 2015	WC per 10cm increase	Ovarian CA inc	C56	0.33	0 (0-61) 0.50	6	0	0.5	NP	0.5	NP	0.4	NP
Aune 2015	WG per 5kg increase	Ovarian CA inc	C56	0.68	67 (0-84) 0.01	6	2	0.3	0.03	0.3	0.04	0.3	0.04

Aune 2015	Weight per 5kg increase	Ovarian CA inc	C56	0.42	7 (0-70) 0.36	4	1	0.2	0.22	0.2	0.22	0.2	0.20
Aune 2015	WHR per 0.1 unit increase	Ovarian CA inc	C56	0.22	0 (0-64) 0.48	5	0	0.3	NP	0.3	NP	0.3	NP
Poorolajal 2014	BMI 25-29.99 vs. < 25	Ovarian CA inc, PoMP	C56	0.70	52 (0-80) 0.08	5	1	0.3	0.14	1.3	0.17	1.0	0.96
Poorolajal 2014	BMI 30+ vs. < 25	Ovarian CA inc, PoMP	C56	0.52	46 (0-79) 0.12	5	1	1.7	NP	1.9	NP	0.3	0.14
Keum 2015	WG per 5kg increase	Ovarian CA inc, PoMP, HRT-	C54.1	NA	0 (.-.) 0.50	2	1	0.2	0.20	0.2	0.20	0.27	0.25
Poorolajal 2014	BMI 25-29.99 vs. < 25	Ovarian CA inc, PrMP	C56	0.50	0 (0-73) 0.51	3	0	0.6	NP	0.6	NP	1.1	NP
Poorolajal 2014	BMI 30+ vs. < 25	Ovarian CA inc, PrMP	C56	0.61	0 (0-73) 0.86	3	1	1.7	NP	1.7	NP	1.7	NP
Zhang 2015	BMI obese vs. normal weight**	Prostate CA inc	C61	0.64	40 (0-64) 0.04	14	2	1.6	0.66	0.7	0.16	7.8	NP
Renehan 2008	BMI per 5kg/m^2 increase	Prostate CA inc	C61	0.72	76 (64-83) <0.01	26	6	3.0	0.11	3.0	0.11	6.1	NP
MacInnis 2006	WC per 10cm increase	Prostate CA inc	C61	0.44	0 (0-68) 0.41	4	0	0.3	NP	0.3	NP	0.4	NP
Keum 2015	WG per 5kg increase	Prostate CA inc	C61	0.67	51 (0-82) 0.11	4	1	0.3	0.25	0.3	0.30	0.2	0.20
Keum 2015	WG: highest vs. lowest category	Prostate CA inc	C61	0.67	31 (0-69) 0.18	8	0	0.5	NP	0.7	NP	0.9	NP
Discaccati 2012	BMI per 5kg/m^2 increase	Prostate CA inc, adv	C61	0.02	40 (0-68) 0.07	13	3	1.7	0.24	2.5	0.72	0.6	0.02
Discaccati 2012	BMI per 5kg/m^2 increase	Prostate CA inc, loc	C61	0.46	20 (0-59) 0.25	12	3	2.3	0.71	2.7	0.74	1.7	0.40
Keum 2015	WG per 5kg increase	Prostate CA inc, advanced	C61	<0.01	0 (0-68) 0.46	4	1	0.3	0.13	0.3	0.13	0.3	0.22
Keum 2015	WG per 5kg increase	Prostate CA inc, high screening rate	C61	NA	0 (.-.) 0.44	2	1	0.5	0.41	0.5	0.41	0.4	0.25
Keum 2015	WG per 5kg increase	Prostate CA inc, localised	C61	0.12	35 (0-78) 0.20	4	1	0.4	0.28	0.5	0.45	0.22	0.08
Keum 2015	WG per 5kg increase	Prostate CA inc, low screening rate	C61	NA	0 (.-.) 1.00	2	0	0.1	NP	0.1	NP	0.1	NP
Zhang 2015	BMI obese vs. normal weight**	Prostate CA mort	C61	0.03	0 (0-53) 0.85	10	4	6.2	NP	6.2	NP	5.7	NP
Cao 2011	BMI per 5kg/m^2 increase	Prostate CA mort	C61	0.30	59 (0-81) 0.03	6	4	2.0	0.10	2.9	0.44	1.6	0.04<br>9
Yang 2015	BS; surgery vs. no surgery	Kidney CA inc	C64	NA	0	2	0	0.1	NP	0.1	NP	0.1	NP
Wang 2014	BMI 25-29.99 vs. < 25	Kidney CA inc	C64	0.003	36 (0-55) 0.01	43	19	28.4	NP	32.7	NP	19.0	1.00
Wang 2014	BMI 30+ vs. < 25	Kidney CA inc	C64	0.53	44 (6-63) <0.01	31	22	30.0	NP	30.0	NP	30.2	NP
Renehan 2008	BMI per 5kg/m^2 increase	Kidney CA inc	C64	0.11	49 (0-69) <0.01	19	14	10.9	0.17	11.8	0.35	7.9	<0.0<br>1

Keum 2015	WG: highest vs. lowest category	Kidney CA inc	C64	0.89	10 (0-75) 0.33	3	1	3.0	NP	3.0	NP	2.0	NP
Renehan 2008	BMI per 5kg/m^2 increase	Kidney CA inc, men	C64	0.16	22 (0-64) 0.24	9	5	4.5	1.00	4.9	1.00	4.0	0.52
Renehan 2008	BMI per 5kg/m^2 increase	Kidney CA inc, wom	C64	0.39	45 (0-72) 0.06	10	9	6.4	0.11	6.6	0.18	6.9	0.19
Sun 2015	BMI 25-29.99 vs. < 25	Bladder CA inc	C67	0.29	38 (0-60) 0.03	26	5	4.2	0.59	5.6	NP	3.7	0.41
Sun 2015	BMI 30+ vs. < 25	Bladder CA inc	C67	0.71	15 (0-48) 0.24	26	4	10.8	NP	10.0	NP	13.4	NP
Sun 2015	BMI per 5kg/m^2 increase	Bladder CA inc	C67	0.86	32 (0-69) 0.17	8	2	1.0	0.28	1.1	0.31	0.8	0.18
Shao 2014	BMI 25-29.99 vs. < 25	Meningeoma inc	C70	0.87	0 (0-68) 0.43	4	0	0.5	NP	0.5	NP	0.2	NP
Shao 2014	BMI 30+ vs. < 25	Meningeoma inc	C70	0.54	0 (0-68) 0.45	4	2	3.3	NP	3.3	NP	2.6	NP
Ma 2015	BMI 30+ vs. < 25	Thyroid CA inc	C73	<0.01	21 (0-52) 0.18	24	9	14.8	NP	18.0	NP	11.3	NP
Renehan 2008	BMI per 5kg/m^2 increase	Thyroid CA inc	C73	0.09	62 (0-82) 0.01	7	4	2.7	0.43	3.2	0.71	2.4	0.24
Keum 2015	WG: highest vs. lowest category	Thyroid CA inc	C73	0.14	0 (0-68) 0.42	4	0	0.3	NP	0.3	NP	0.7	NP
Renehan 2008	BMI per 5kg/m^2 increase	Thyroid CA inc, men	C73	0.23	77 (0-90) <0.01	4	2	1.6	1.00	2.5	NP	1.0	0.27
Renehan 2008	BMI per 5kg/m^2 increase	Thyroid CA inc, wom	C73	0.41	3 (0-74) 0.36	3	2	1.1	0.56	1.2	0.56	1.1	0.30
Larsson 2011	BMI 25-29.99 vs. < 25	Hodgkin's lymphoma inc	C81	0.10	17 (0-65) 0.30	7	1	0.5	0.43	0.4	0.35	1.7	NP
Larsson 2011	BMI 30+ vs. < 25	Hodgkin's lymphoma inc	C81	0.74	22 (0-74) 0.28	4	1	3.1	NP	3.1	NP	3.5	NP
Yang 2015	BS; surgery vs. no surgery	NHL inc	C82-C85	NA	0	2	0	0.5	NP	0.5	NP	0.5	NP
Larsson 2007	BMI 25-29.99 vs. < 25	NHL inc	C82-C85	0.45	28 (0-61) 0.15	15	1	2.2	NP	3.0	NP	1.5	NP
Larsson 2007	BMI 30+ vs. < 25	NHL inc	C82-C85	0.91	66 (33-79) <0.01	15	5	6.8	NP	7.1	NP	4.5	0.78
Larsson 2011	BMI per 5kg/m^2 increase	NHL inc	C82-C85	0.26	9 (0-45) 0.34	23	5	4.7	0.80	4.8	1.00	2.7	0.18
Larsson 2011	BMI per 5kg/m^2 increase	NHL mort	C82-C85	0.57	53 (0-78) 0.05	7	3	3.1	NP	2.9	1.00	3.4	NP
Castillo 2014	BMI 25-29.99 vs. < 25	Diff. large B-cell lymphoma inc	C83.3	0.37	0 (0-53) 0.65	10	1	1.8	NP	1.8	NP	5.7	NP
Castillo 2014	BMI 30+ vs. < 25	Diff. large B-cell lymphoma inc	C83.3	0.05	27 (0-63) 0.18	12	3	6.6	NP	5.9	NP	11.6	NP
Yang 2015	BS; surgery vs. no surgery	Multiple myeloma inc	C90.0	NA	0	2	0	0.2	NP	0.2	NP	0.2	NP
Wallin 2012	BMI 25-29.99 vs. < 25	Multiple myeloma inc	C90.0	0.72	0 (0-42) 0.73	20	2	4.8	NP	4.8	NP	5.3	NP

Wallin 2012	BMI 30+ vs. < 25	Multiple myeloma inc	C90.0	0.56	34 (0-61) 0.07	19	5	8.7	NP	7.5	NP	11.2	NP
Renehan 2008	BMI per 5kg/m^2 increase	Multiple myeloma inc	C90.0	0.65	0 (0-49) 0.66	13	4	3.6	0.76	3.6	0.76	3.5	0.76
Renehan 2008	BMI per 5kg/m^2 increase	Multiple myeloma inc, men	C90.0	0.76	5 (0-61) 0.39	7	2	1.9	1.00	1.9	1.00	2.0	NP
Renehan 2008	BMI per 5kg/m^2 increase	Multiple myeloma inc, wom	C90.0	0.82	0 (0-61) 0.68	6	2	1.7	0.68	1.7	0.68	1.7	0.67
Wallin 2012	BMI 25-29.99 vs. < 25	Multiple myeloma mort	C90.0	0.46	0 (0-56) 0.58	8	1	2.3	NP	2.3	NP	2.8	NP
Wallin 2012	BMI 30+ vs. < 25	Mult myel mort	C90.0	0.32	0 (0-58) 0.60	7	4	5.1	NP	5.1	NP	4.7	NP
Castillo 2012	BMI 30+ vs. < 25	Leukemia inc	C91-C92	0.29	43 (0-63) 0.01	25	7	16.4	NP	15.2	NP	19.3	NP
Castillo 2012	BMI 30+ vs. < 25	Leukemia mort	C91-C92	0.99	30 (0-68) 0.19	8	4	5.9	NP	6.0	NP	6.6	NP
Renehan 2008	BMI per 5kg/m^2 increase	Leukemia inc	C91-C95	0.22	51 (0-76) 0.04	8	4	3.0	0.48	3.3	0.72	2.4	0.25
Renehan 2008	BMI per 5kg/m^2 increase	Leukemia inc, men	C91-C95	0.47	0 (0-64) 0.42	5	1	1.4	NP	1.4	NP	1.5	NP
Renehan 2008	BMI per 5kg/m^2 increase	Leukemia inc, wom	C91-C95	0.20	79 (0-92) <0.01	3	3	1.4	0.11	1.9	0.29	1.1	0.04<br>7
Larsson 2008	BMI 30+ vs. < 25	Leukaemia inc, ALL	C91	0.67	7 (0-66) 0.37	5	1	3.6	NP	3.6	NP	1.9	NP
Larsson 2008	BMI 30+ vs. < 25	Leukaemia inc, CLL	C91	0.78	31 (0-74) 0.21	5	2	4.0	NP	4.0	NP	4.2	NP
Larsson 2008	BMI 30+ vs. < 25	Leukaemia inc, AML	C92	0.27	79 (44-89) <0.001	6	4	4.9	NP	5.4	NP	3.4	0.61
Larsson 2008	BMI 30+ vs. < 25	Leukaemia inc, CML	C92	0.47	0 (0-64) 0.51	5	1	3.4	NP	3.4	NP	1.9	NP

Abbreviations: AdCa, adenocarcinoma; adv, advanced; ALL, acute lymphatic leukemia; AML, acutel myeloic leukemia; ASE, adjusted for sunlight exposure; biliary tract system cancer, includes cancers of gallbladder, extrahepatic bile duct and ampulla of Vateri; BMI, Body Mass Index; BMI iya, Body Mass Index in young adulthood; BS, bariatric surgery; CA, cancer; CI, confidence interval; CLL, chronic lymphatic leukemia; CML, chronic myeloic leukemia; ER = estrogen receptor status; HC, hip circumference; HRT, hormone replacement therapy; inc, incidence; loc, localised; mort, mortality; NA: Not available, due to <3 included studies; NHL, Non-Hodgkin lymphoma; NP: Not Pertinent, because the estimated is larger than the observed, and there is no evidence of excess statistical significance based on the assumption made for the plausible effect size; PoMP, postmenopausal; PR = progesterone receptor status; PrMP, premenopausal; RR, relative risk; SqCa, squamous cell carcinoma; WC, waist circumference; WG, weight gain; WHR, waist-to-hip ratio;

¶ P-value from te Egger's regression asymmetry test.

‡ I^2 metric of inconsistency (95% CI) and the P-value of the Q test.

§ Expected number of statistically significant studies using the point estimate of each meta-analysis (from fixed effect, random effect of largest study accordingly) as the plausible effect size.

|| P value of the excess statistical significance test.

** cut-offs in categories varied between included studies

±± BMI, weight gain or weight change, varies between included studies

All statistical tests were two-sided.

Supplementary Table 3: Excluded studies and studies selected in their place to be included in the analysis.

Author, year	Exposure	Association of obesity or weight change with	ICD-10	N studies	N cohort Studies	Summary relative risk & 95% CI|	Included
Renehan 2008 (1)	BMI per 5kg/m^2 increase	Oesophageal AdCa inc	C15	8	8	1.53 (1.41-1.67)	Yes
Turati 2013 (2)	BMI 25-29.99 vs. < 25	Oesophageal and gastric cardia AdCa inc	C15-C16	10	10	1.71 (1.50-1.96)	Yes
Turati 2013	BMI 30+ vs. < 25	Oesophageal and gastric cardia AdCa inc	C15-C16	9	9	2.34 (1.95-2.81)	Yes
Hampel 2005 (3)	BMI 25-29.99 vs. < 25	Oesophageal AdCa inc	C15	6	0	1.51 (1.15-2.01)	No
Hampel 2005	BMI 30+ vs. < 25	Oesophageal AdCa inc	C15	6	0	2.78 (1.85-4.16)	No
Lin 2014 (4)	BMI 25-29.99 vs. < 25	Gastric cancer inc	C16	15	12	1.04 (0.96-1.12)	Yes
Lin 2014	BMI 30+ vs. < 25	Gastric cancer inc	C16	15	11	1.13 (1.03-1.24)	Yes
Yang 2009 (5)	BMI > 25 vs. < 25	Gastric cancer inc	C16	10	10	1.22 (1.06-1.41)	No

Chen 2013 (6)	BMI 25-29.99 vs. < 25	Gastric cancer inc	C16	10	10	1.01 (0.96-1.07)	No
Chen 2013	BMI 30+ vs. < 25	Gastric cancer inc	C16	13	13	1.06 (0.99-1.12)	No
Yang 2015 (7)	bariatric surgery	Colorectal cancer inc.	C18-C20	5	5	0.77 (0.62-0.96)	Yes
Afshar 2014 (8)	bariatric surgery	Colorectal cancer inc.	C18-C20	4	4	0.73 (0.58-0.90)	No
Wang 2012 (9)	BMI per 5kg/m^2 increase	Liver cancer inc	C22	21	19	1.39 (1.25-1.55)	Yes
Chen 2012 (10)	BMI 25-29.99 vs. < 25	Liver cancer inc	C22	14	14	1.18 (1.06-1.31)	Yes
Chen 2012	BMI 30+ vs. < 25	Liver cancer inc	C22	19	19	1.83 (1.59-2.11)	Yes
Tanaka 2012 (11)	BMI per 1kg/m^2 increase	Liver cancer inc	C22	10	10	1.07 (1.03-1.10)	No
Larsson 2007 (12)	BMI 25-29.99 vs. < 25	Liver cancer inc	C22	11	11	1.17 (1.02-1.34)	No
Larsson 2007	BMI 30+ vs. < 25	Liver cancer inc	C22	11	11	1.89 (1.51-2.36)	No
Aune 2012 (13)	BMI per 5kg/m^2 increase	Pancreatic cancer inc.	C25	22	22	1.10 (1.06-1.14)	Yes
Berrington de Gonzales 2003 (14)	BMI per 1kg/m^2 increase	Pancreatic cancer inc.	C25	14	7	1.02 (1.01-1.03)	No
Larsson 2007 (15)	BMI per 1kg/m^2 increase	Pancreatic cancer inc.	C25	21	21	1.12 (1.06-1.17)	No
Suzuki 2009 (16)	Obesity±, high vs. low*	Breast cancer inc, ER- PR-, MxMP	C50	4	1	0.96 (0.79-1.17)	Yes
Suzuki 2009	Obesity±, high vs. low*	Breast cancer inc, ER- PR-, PoMP	C50	10	4	1.06 (0.84-1.33)	Yes
Suzuki 2009	Obesity±, high vs. low*	Breast cancer inc, ER- PR-, PrMP	C50	6	6	1.03 (0.87-1.22)	Yes
Suzuki 2009	Obesity±, high vs. low*	Breast cancer inc, ER- PR+, MxMP	C50	2	1	1.12 (0.79-1.58)	Yes
Suzuki 2009	Obesity±, high vs. low*	Breast cancer inc, ER- PR+, PoMP	C50	4	2	2.01 (1.22-3.31)	Yes
Suzuki 2009	Obesity±, high vs. low*	Breast cancer inc, ER- PR+, PrMP	C50	2	2	1.01 (0.73-1.39)	Yes
Suzuki 2009	Obesity±, high vs. low*	Breast cancer inc, ER+ PR-, MxMP	C50	2	1	0.82 (0.66-1.02)	Yes
Suzuki 2009	Obesity±, high vs. low*	Breast cancer inc, ER+ PR-, PoMP	C50	6	2	0.93 (0.72-1.21)	Yes
Suzuki 2009	Obesity±, high vs. low*	Breast cancer inc, ER+ PR-, PrMP	C50	3	3	1.06 (0.76-1.49)	Yes
Suzuki 2009	Obesity±, high vs. low*	Breast cancer inc, ER+ PR+, MxMP	C50	4	1	1.28 (0.91-1.82)	Yes
Suzuki 2009	Obesity±, high vs. low*	Breast cancer inc, ER+ PR+, PoMP	C50	10	4	1.82 (1.55-2.14)	Yes

Suzuki 2009	Obesity±, high vs. low*	Breast cancer inc, ER+ PR+, PrMP	C50	6	6	0.80 (0.70-0.92)	Yes
Renehan 2008 (1)	BMI per 5kg/m^2 increase	Breast cancer inc, PrMP	C50	20	20	0.92 (0.88-0.96)	Yes
Renehan 2008	BMI per 5kg/m^2 increase	Breast cancer inc, PoMP	C50	31	29	1.12 (1.08-1.16)	Yes
Keum 2015 (17)	WG per 5kg increase	Breast CA inc, PrMP	C50	3	3	0.99 (0.95-1.03)	Yes
Keum 2015	WG: highest vs. lowest category	Breast CA inc, PrMP	C50	4	4	0.99 (0.81-1.21)	Yes
Keum 2015	WG per 5kg increase	Breast CA inc, PoMP	C50	7	7	1.11 (1.09-1.13)	Yes
Keum 2015	WG: highest vs. lowest category	Breast CA inc, PoMP	C50	7	7	1.75 (1.53-2.00)	Yes
Pierobon 2013 (18)	BMI, obese vs. normal weight*	Breast cancer inc, triple negative, case-case	C50	11	0	1.20 (1.03-1.40)	Yes
Pierobon 2013	BMI, obese vs. normal weight*	Breast cancer inc, triple negative, case-control	C50	5	0	1.24 (1.06-1.46)	Yes
Cheraghi 2012 (19)	BMI 25-29.99 vs. < 25	Breast cancer inc, PrMP	C50	4	4	1.01 (0.77-1.31)	Yes
Cheraghi 2012	BMI 30+ vs. < 25	Breast cancer inc, PrMP	C50	4	4	0.91 (0.70-1.18)	Yes
Cheraghi 2012	BMI 25-29.99 vs. < 25	Breast cancer inc, PoMP	C50	8	8	1.12 (1.06-1.18)	Yes
Cheraghi 2012	BMI 30+ vs. < 25	Breast cancer inc, PoMP	C50	8	8	1.16 (1.08-1.25)	Yes
Jung 2009 (20)	BMI, obese vs. normal weight*	Breast cancer inc, PrMP	C50	11	1	1.39 (1.13-1.70)	No
Jung 2009	BMI, obese vs. normal weight*	Breast cancer inc, PoMP	C50	11	1	1.68 (1.15-2.46)	No
Vrieling 2010 (21)	WG, highest vs. lowest	Breast cancer inc, ER-, PR-, PoMP	C50	3	3	1.23 (0.57-1.90)	No
Vrieling 2010	WG, highest vs. lowest	Breast cancer inc, ER+, PR+, PoMP	C50	3	3	2.17 (1.48-2.85)	No
Discaccati 2012 (22)	BMI per 5kg/m^2 increase	Prostate cancer inc, loc	C61	12	12	0.94 (0.91-0.97)	Yes
Discaccati 2012	BMI per 5kg/m^2 increase	Prostate cancer inc, adv	C61	13	13	1.09 (1.02-1.16)	Yes
Hu 2014 (23)	BMI per 5kg/m^2 increase	Prostate cancer inc	C61	8	8	1.18 (1.00-1.39)	No
Hu 2014	BMI per 5kg/m^2 increase	Prostate cancer inc, HG only	C61	6	6	1.50 (1.31-1.70)	No
Aune 2015 (24)	BMI per 5kg/m^2 increase	Ovarian cancer inc	C56	25	24	1.07 (1.03-1.12)	Yes
Coll. Gr. (25)	BMI per 5kg/m^2 increase	Ovarian cancer inc	C56	17	17	1.03 (1.00-1.06)	No
Sun 2015 (26)	BMI 25-29.99 vs. < 25	Bladder CA inc	C67	26	26	1.07 (1.01-1.14)	Yes

Sun 2015	BMI 30+ vs. < 25	Bladder CA inc	C67	26	26	1.09 (1.04-1.15)	Yes
Qin 2013 (27)	BMI, obese vs. normal weight**	Bladder CA inc	C67	11	11	1.10 (1.06-1.16)	No
Wallin 2012 (28)	BMI 25-29.99 vs. < 25	Multiple myeloma inc	C90.0	20	20	1.12 (1.07-1.18)	Yes
Wallin 2012	BMI 30+ vs. < 25	Multiple myeloma inc	C90.0	19	19	1.21 (1.08-1.35)	Yes
Larsson 2007 (29)	BMI 25-29.99 vs. < 25	Multiple myeloma inc	C90.0	11	11	1.12 (1.07-1.18)	No
Larsson 2007	BMI 30+ vs. < 25	Multiple myeloma inc	C90.0	11	11	1.27 (1.15-1.41)	No

Abbreviations: AdCa, adenocarcinoma; adv, advanced; BMI, Body Mass Index; CA, cancer; CI, confidence interval; ER = estrogen receptor status; inc, incidence; loc, localised; mort, mortality; PoMP, postmenopausal; PR = progesterone receptor status; PrMP, premenopausal; RR, relative risk; WG, weight gain.

± BMI, weight gain or weight change, varies between included studies
* cut-offs for categories varied between studies
| Summary relative risk of random effects model including all study types.

References for supplementary table 3:

1. Renehan AG, Tyson M, Egger M, Heller RF, Zwahlen M. Body-mass index and incidence of cancer: a systematic review and meta-analysis of prospective observational studies. Lancet. 2008;371(9612):569-78.
2. Turati F, Tramacere I, La Vecchia C, Negri E. A meta-analysis of body mass index and esophageal and gastric cardia adenocarcinoma. Ann Oncol. 2013;24(3):609-17.
3. Hampel H, Abraham NS, El-Serag HB. Meta-analysis: obesity and the risk for gastroesophageal reflux disease and its complications. Ann Intern Med. 2005;143(3):199-211.
4. Lin XJ, Wang CP, Liu XD, Yan KK, Li S, Bao HH, et al. Body mass index and risk of gastric cancer: a meta-analysis. Jpn J Clin Oncol. 2014;44(9):783-91.
5. Yang P, Zhou Y, Chen B, Wan HW, Jia GQ, Bai HL, et al. Overweight, obesity and gastric cancer risk: results from a meta-analysis of cohort studies. Eur J Cancer. 2009;45(16):2867-73.

6. Chen Y, Liu L, Wang X, Wang J, Yan Z, Cheng J, et al. Body mass index and risk of gastric cancer: a meta-analysis of a population with more than ten million from 24 prospective studies. Cancer Epidemiol Biomarkers Prev. 2013;22(8):1395-408.
7. Yang XW, Li PZ, Zhu LY, Zhu S. Effects of bariatric surgery on incidence of obesity-related cancers: a meta-analysis. Med Sci Monit. 2015;21:1350-7.
8. Afshar S, Kelly SB, Seymour K, Lara J, Woodcock S, Mathers JC. The effects of bariatric surgery on colorectal cancer risk: systematic review and meta-analysis. Obes Surg. 2014;24(10):1793-9.
9. Wang Y, Wang B, Shen F, Fan J, Cao H. Body mass index and risk of primary liver cancer: a meta-analysis of prospective studies. Oncologist. 2012;17(11):1461-8.
10. Chen Y, Wang X, Wang J, Yan Z, Luo J. Excess body weight and the risk of primary liver cancer: an updated meta-analysis of prospective studies. Eur J Cancer. 2012;48(14):2137-45.
11. Tanaka K, Tsuji I, Tamakoshi A, Matsuo K, Ito H, Wakai K, et al. Obesity and liver cancer risk: an evaluation based on a systematic review of epidemiologic evidence among the Japanese population. Jpn J Clin Oncol. 2012;42(3):212-21.
12. Larsson SC, Wolk A. Overweight, obesity and risk of liver cancer: a meta-analysis of cohort studies. Br J Cancer. 2007;97(7):1005-8.
13. Aune D, Greenwood DC, Chan DS, Vieira R, Vieira AR, Navarro Rosenblatt DA, et al. Body mass index, abdominal fatness and pancreatic cancer risk: a systematic review and non-linear dose-response meta-analysis of prospective studies. Ann Oncol. 2012;23(4):843-52.
14. Berrington de Gonzalez A, Sweetland S, Spencer E. A meta-analysis of obesity and the risk of pancreatic cancer. Br J Cancer. 2003;89(3):519-23.
15. Larsson SC, Orsini N, Wolk A. Body mass index and pancreatic cancer risk: A meta-analysis of prospective studies. Int J Cancer. 2007;120(9):1993-8.
16. Suzuki R, Orsini N, Saji S, Key TJ, Wolk A. Body weight and incidence of breast cancer defined by estrogen and progesterone receptor status--a meta-analysis. Int J Cancer. 2009;124(3):698-712.
17. Keum N, Greenwood DC, Lee DH, Kim R, Aune D, Ju W, et al. Adult weight gain and adiposity-related cancers: a dose-response meta-analysis of prospective observational studies. J Natl Cancer Inst. 2015;107(2).
18. Pierobon M, Frankenfeld CL. Obesity as a risk factor for triple-negative breast cancers: a systematic review and meta-analysis. Breast Cancer Res Treat. 2013;137(1):307-14.
19. Cheraghi Z, Poorolajal J, Hashem T, Esmailnasab N, Doosti Irani A. Effect of body mass index on breast cancer during premenopausal and postmenopausal periods: a meta-analysis. PLoS One. 2012;7(12):e51446.

20. Jung D, Lee SM. BMI and Breast Cancer in Korean Women: A Meta-Analysis. Asian Nurs Res (Korean Soc Nurs Sci). 2009;3(1):31-40.
21. Vrieling A, Buck K, Kaaks R, Chang-Claude J. Adult weight gain in relation to breast cancer risk by estrogen and progesterone receptor status: a meta-analysis. Breast Cancer Res Treat. 2010;123(3):641-9.
22. Discacciati A, Orsini N, Wolk A. Body mass index and incidence of localized and advanced prostate cancer--a dose-response meta-analysis of prospective studies. Ann Oncol. 2012;23(7):1665-71.
23. Hu MB, Liu SH, Jiang HW, Bai PD, Ding Q. Obesity affects the biopsy-mediated detection of prostate cancer, particularly high-grade prostate cancer: a dose-response meta-analysis of 29,464 patients. PLoS One. 2014;9(9):e106677.
24. Aune D, Navarro Rosenblatt DA, Chan DS, Abar L, Vingeliene S, Vieira AR, et al. Anthropometric factors and ovarian cancer risk: a systematic review and nonlinear dose-response meta-analysis of prospective studies. Int J Cancer. 2015;136(8):1888-98.
25. Collaborative Group on Epidemiological Studies of Ovarian C. Ovarian cancer and body size: individual participant meta-analysis including 25,157 women with ovarian cancer from 47 epidemiological studies. PLoS Med. 2012;9(4):e1001200.
26. Sun JW, Zhao LG, Yang Y, Ma X, Wang YY, Xiang YB. Obesity and risk of bladder cancer: a dose-response meta-analysis of 15 cohort studies. PLoS One. 2015;10(3):e0119313.
27. Qin Q, Xu X, Wang X, Zheng XY. Obesity and risk of bladder cancer: a meta-analysis of cohort studies. Asian Pac J Cancer Prev. 2013;14(5):3117-21.
28. Wallin A, Larsson SC. Body mass index and risk of multiple myeloma: a meta-analysis of prospective studies. Eur J Cancer. 2011;47(11):1606-15.
29. Larsson SC, Wolk A. Body mass index and risk of multiple myeloma: a meta-analysis. Int J Cancer. 2007;121(11):2512-6.

Supplementary Table 4: Sensitivity analysis using credibility ceilings of 10% in the 194 meta-analyses investigating the associations between obesity and the risk of cancer development or mort (only cohort studies included).

Author, year	Exposure	Association of obesity or weight change with	ICD-10	Studies	Credibility Ceiling 0%			Credibility Ceiling 10%		
					RR (95% CI)	I^2 (%)	LR	RR (95% CI)	I^2 (%)	LR
Casagrande 2014	BS inc density rate after surgery	Overall CA inc	C00-C97	9	1.10 (0.67-1.80)	96	0.4	1.29 (0.87-1.90)	22	4.0
Casagrande 2014	BS; surgery vs. no surgery	Overall CA inc	C00-C97	4	0.40 (0.22-0.71)	94	531.9	0.69 (0.49-0.97)	0	28.6
Renehan 2008	BMI per 5kg/m^2 increase	Oesophageal AdCa inc	C15	8	1.53 (1.41-1.67)	0	> 1000.0	1.36 (1.12-1.65)	0	606.7
Singh 2013	Central adiposity	Oesophageal AdCa inc	C15	2	1.78 (1.26-2.52)	0	992.9	1.73 (0.93-1.21)	0	11.0
Renehan 2008	BMI per 5kg/m^2 increase	Oesophageal AdCa inc, men	C15	5	1.52 (1.33-1.74)	24	> 1000.0	1.31 (1.04-1.64)	0	47.6
Renehan 2008	BMI per 5kg/m^2 increase	Oesophageal AdCa inc, women	C15	3	1.51 (1.30-1.75)	0	> 1000.0	1.49 (1.05-2.12)	0	36.5
Renehan 2008	BMI per 5kg/m^2 increase	Oesophageal SqCa inc	C15	5	0.63 (0.53-0.75)	80	> 1000.0	0.68 (0.52-0.91)	0	126.4

Renehan 2008	BMI per 5kg/m^2 increase	Oesophageal SqCa inc, men	C15	3	0.71 (0.61-0.84)	44	> 1000.0	0.72 (0.52-0.98)	0	24.8
Renehan 2008	BMI per 5kg/m^2 increase	Oesophageal SqCa inc, women	C15	2	0.57 (0.48-0.67)	58	> 1000.0	0.57 (0.31-1.05)	0	12.7
Turati 2013	BMI 25-29.99 vs. < 25	Gastrc cardia and oesophageal AdCa	C15-C16.0	10	1.48 (1.35-1.61)	0	> 1000.0	1.41 (1.18-1.68)	0	> 1000.0
Turati 2013	BMI 30+ vs. < 25	Gastrc cardia and oesophageal AdCa	C15-C16.0	9	2.19 (1.84-2.58)	34	> 1000.0	1.93 (1.35-2.76)	0	> 1000.0
Lin 2014	BMI 25-29.99 vs. < 25	Gastric CA inc	C16	12	1.00 (0.93-1.08)	30	0.1	1.00 (0.94-1.06)	0	0.01
Lin 2014	BMI 30+ vs. < 25	Gastric CA inc	C16	11	1.10 (1.00-1.22)	7	17.9	1.07 (0.95-1.20)	0	2.8
Renehan 2008	BMI per 5kg/m^2 increase	Gastric CA inc	C16	13	0.99 (0.92-1.06)	37	0.4	0.98 (0.93-1.03)	0	1.0
Renehan 2008	BMI per 5kg/m^2 increase	Gastric CA inc, men	C16	8	0.97 (0.88-1.06)	39	1.1	0.98 (0.92-1.04)	0	1.1
Renehan 2008	BMI per 5kg/m^2 increase	Gastric CA inc, women	C16	5	1.04 (0.91-1.21)	41	0.8	1.03 (0.90-1.17)	30	0.4
Larsson 2007	BMI per 5kg/m^2 increase	Colon CA inc	C18	44	1.22 (1.17-1.28)	84	> 1000.0	1.08 (1.05-1.11)	0	> 1000.0
Larsson 2007	WC per 10cm increase	Colon CA inc	C18	8	1.25 (1.15-1.35)	50	> 1000.0	1.18 (1.07-1.31)	0	797.0
Larsson 2007	WHR per 0.1 unit increase	Colon CA inc	C18	8	1.29 (1.17-1.43)	53	> 1000.0	1.17 (1.05-1.31)	0	124.3
Keum 2015	WG per 5kg increase	Colon CA inc	C18	7	1.07 (1.03-1.10)	0	> 1000.0	1.04 (1.00-1.09)	0	21.7
Moghaddam 2007	BMI 30+ vs. < 25	Colon CA inc	C18	28	1.44 (1.28-1.62)	76	> 1000.0	1.15 (1.06-1.24)	0	> 1000.0
Larsson 2007	BMI per 5kg/m^2 increase	Colon CA inc, men	C18	24	1.30 (1.25-1.35)	24	> 1000.0	1.24 (1.14-1.34)	0	> 1000.0
Larsson 2007	WC per 10cm increase	Colon CA inc, men	C18	5	1.33 (1.18-1.50)	45	> 1000.0	1.23 (1.04-1.44)	0	73.4
Larsson 2007	WHR per 0.1 unit increase	Colon CA inc, men	C18	4	1.43 (1.19-1.71)	56	> 1000.0	1.33 (1.05-1.68)	0	56.3
Keum 2015	WG per 5kg increase	Colon CA inc, men	C18	4	1.09 (1.05-1.14)	0	> 1000.0	1.07 (1.00-1.14)	0	22.4
Moghaddam 2007	BMI 30+ vs. < 25	Colon CA inc, men	C18	14	1.53 (1.44-1.63)	0	> 1000.0	1.60 (1.28-1.98)	0	> 1000.0
Larsson 2007	BMI per 5kg/m^2 increase	Colon CA inc, women	C18	20	1.12 (1.06-1.17)	59	> 1000.0	1.05 (1.02-1.09)	0	334.0
Larsson 2007	WC per 10cm increase	Colon CA inc, women	C18	3	1.16 (1.08-1.23)	0	> 1000.0	1.16 (1.01-1.32)	0	30.5
Larsson 2007	WHR per 0.1 units increase	Colon CA inc, women	C18	4	1.20 (1.08-1.33)	30	> 1000.0	1.12 (0.98-1.28)	0	10.5
Keum 2015	WG per 5kg increase	Colon CA inc, women	C18	3	1.03 (0.98-1.09)	0	3.2	1.03 (0.97-1.08)	0	1.8
Moghaddam 2007	BMI 30+ vs. < 25	Colon CA inc, women	C18	14	1.22 (1.07-1.40)	51	294.7	1.09 (1.01-1.19)	0	30.5

Robsham 2013	BMI, Obese vs. normal**	Colon CA inc; distal colon	C18	20	1.59 (1.34-1.89)	44	> 1000.0	1.25 (1.07-1.46)	0	193.7
Robsham 2013	BMI, Obese vs. normal**	Colon CA inc; distal colon, men	C18	7	1.70 (1.36-2.11)	17	> 1000.0	1.53 (1.12-2.09)	0	122.4
Robsham 2013	BMI, Obese vs. normal**	Colon CA inc; distal colon, women	C18	12	1.53 (1.19-1.97)	56	> 1000.0	1.17 (0.97-1.40)	0	9.4
Robsham 2013	BMI, Obese vs. normal**	Colon CA inc; proximal colon	C18	20	1.33 (1.12-1.57)	33	> 1000.0	1.14 (1.00-1.29)	0	21.0
Robsham 2013	BMI, Obese vs. normal**	Colon CA inc; proximal colon, men	C18	7	1.44 (1.18-1.77)	0	> 1000.0	1.30 (1.03-1.64)	0	34.4
Robsham 2013	BMI, Obese vs. normal**	Colon CA inc; proximal colon, women	C18	12	1.18 (0.96-1.44)	25	7.8	1.07 (0.92-1.24)	0	1.6
Yang 2015	BS; surgery vs. no surgery	Colorectal CA inc	C18-C20	5	0.77 (0.62-0.96)	0	45.3	0.78 (0.61-0.99)	0	23.0
Ma 2013	BMI, Obese vs. normal weight*	Colorectal CA inc	C18-C20	72	1.33 (1.25-1.42)	69	> 1000.0	1.11 (1.06-1.16)	0	> 1000.0
Ma 2013	WC, highest vs. lowest cat	Colorectal CA inc	C18-C20	19	1.45 (1.33-1.59)	9	> 1000.0	1.35 (1.19-1.55)	0	> 1000.0
Schlesinger 2015	WG per 1kg increase / year	Colorectal CA inc	C18-C20	17	1.20 (1.10-1.31)	37	> 1000.0	1.11 (1.03-1.21)	0	103.3
Schlesinger 2015	WG: high gain vs. stable weight	Colorectal CA inc	C18-C20	19	1.18 (1.12-1.25)	0	> 1000.0	1.11 (1.03-1.20)	0	126.4
Moghaddam 2007	BMI 30+ vs. < 25	Rectal CA inc	C20	21	1.21 (1.10-1.34)	31	> 1000.0	1.07 (1.01-1.14)	0	49.6
Harriss 2009	BMI per 5kg/m^2 increase	Rectal CA inc	C20	31	1.07 (1.03-1.10)	27	> 1000.0	1.03 (1.00-1.05)	0	52.0
Harriss 2009	BMI per 5kg/m^2 increase	Rectal CA inc, men	C20	17	1.09 (1.06-1.13)	6	> 1000.0	1.08 (1.02-1.13)	0	264.9
Moghaddam 2007	BMI 30+ vs. < 25	Rectal CA inc, men	C20	11	1.28 (1.19-1.39)	0	> 1000.0	1.27 (1.06-1.51)	0	102.8
Harriss 2009	BMI per 5kg/m^2 increase	Rectal CA inc, women	C20	14	1.02 (0.99-1.05)	0	4.1	1.02 (0.99-1.04)	0	2.7
Moghaddam 2007	BMI 30+ vs. < 25	Rectal CA inc, women	C20	10	1.09 (0.98-1.22)	7	7.5	1.05 (0.99-1.12)	0	7.2
Chen 2012	BMI 25-29.99 vs. < 25	Liver CA inc	C22	14	1.18 (1.06-1.31)	61	586.5	1.07 (1.01-1.14)	0	38.6
Chen 2012	BMI 30+ vs. < 25	Liver CA inc	C22	19	1.83 (1.59-2.11)	68	> 1000.0	1.51 (1.26-1.81)	0	> 1000.0
Wang 2012	BMI per 5kg/m^2 increase	Liver CA inc	C22	19	1.37 (1.23-1.53)	81	> 1000.0	1.12 (1.04-1.20)	0	441.5
Li 2014	BMI 25-29.99 vs. < 25	Cholangiocarcinoma inc	C22.1, C24	7	1.29 (1.11-1.51)	0	792.8	1.22 (1.00-1.48)	0	20.5
Li 2014	BMI 30+ vs. < 25	Cholangiocarcinoma inc	C22.1, C24	6	1.77 (1.32-2.38)	7	> 1000.0	1.61 (1.15-2.25)	0	186.2
Larsson 2007	BMI 30+ vs. < 25	Gallbladder CA inc	C23	13	1.69 (1.48-1.92)	14	> 1000.0	1.43 (1.15-1.79)	0	816.7
Park 2014	BMI per 5kg/m^2 increase	Biliary tract system CA inc	C23-C24	10	1.56 (1.34-1.81)	25	> 1000.0	1.38 (1.13-1.68)	0	613.6

Yang 2015	BS; surgery vs. no surgery	Pancreatic CA inc	C25	2	0.86 (0.17-4.35)	57	0.2	0.86 (0.17-4.34)	57	0.2
Alsamarrai 2014	BMI, 25+ vs. <25	Pancreatic CA inc	C25	9	1.30 (0.90-1.86)	97	5.3	1.05 (0.93-1.20)	0	1.3
Aune 2012	BMI per 5kg/m^2 increase	Pancreatic CA inc	C25	22	1.10 (1.06-1.14)	25	> 1000.0	1.06 (1.02-1.10)	0	646.4
Aune 2012	WC per 10cm increase	Pancreatic CA inc	C25	5	1.11 (1.05-1.18)	0	> 1000.0	1.11 (1.02-1.20)	0	92.4
Keum 2015	WG per 5kg increase	Pancreatic CA inc	C25	2	1.05 (0.87-1.26)	50	0.6	1.04 (0.87-1.26)	43	0.5
Keum 2015	WG: highest vs. lowest cat.	Pancreatic CA inc	C25	4	1.04 (0.85-1.26)	0	0.4	1.03 (0.84-1.25)	0	0.3
Aune 2012	WHR per 0.1 unit increase	Pancreatic CA inc	C25	4	1.20 (1.09-1.31)	7	> 1000.0	1.14 (1.01-1.28)	0	24.8
Aune 2012	BMI per 5kg/m^2 increase	Pancreatic CA mort	C25	7	1.16 (0.99-1.36)	54	12.6	1.06 (0.98-1.15)	0	6.9
Yang 2013	BMI, 25+ vs. <25	Lung CA inc	C34	20	0.79 (0.73-0.85)	80	> 1000.0	0.94 (0.90-0.98)	0	249.7
Renehan 2008	BMI per 5kg/m^2 increase	Lung CA inc	C34	15	0.77 (0.72-0.83)	71	> 1000.0	0.84 (0.78-0.92)	0	> 1000.0
Renehan 2008	BMI per 5kg/m^2 increase	Lung CA inc, men	C34	10	0.79 (0.73-0.85)	53	> 1000.0	0.83 (0.75-0.92)	0	> 1000.0
Renehan 2008	BMI per 5kg/m^2 increase	Lung CA inc, women	C34	5	0.76 (0.61-0.94)	86	78.6	0.88 (0.75-1.03)	0	8.8
Renehan 2008	BMI per 5kg/m^2 increase	Lung CA in, smokers	C34	4	0.77 (0.69-0.86)	78	> 1000.0	0.81 (0.68-0.97)	0	53.3
Renehan 2008	BMI per 5kg/m^2 increase	Lung CA inc, non-smokers	C34	4	0.89 (0.72-1.09)	54	2.9	0.94 (0.83-1.08)	0	1.6
Yang 2015	BS; surgery vs. no surgery	Melanoma inc	C43	2	0.75 (0.43-1.31)	0	2.2	0.75 (0.43-1.31)	0	2.2
Sergentanis 2013	BMI 25-29.99 vs. < 25	Melanoma inc	C43	13	1.08 (0.94-1.24)	59	2.3	1.02 (0.93-1.11)	6	0.5
Sergentanis 2013	BMI 30+ vs. < 25	Melanoma inc	C43	13	1.07 (0.89-1.30)	67	1.2	1.00 (0.85-1.18)	20	0.01
Sergentanis 2013	BMI, 25+ vs. <25, ASE	Melanoma inc	C43	7	0.86 (0.67-1.10)	25	3.5	0.89 (0.72-1.12)	0	2.1
Renehan 2008	BMI per 5kg/m^2 increase	Melanoma Inc	C43	11	1.07 (0.97-1.17)	79	4.8	0.99 (0.94-1.05)	10	0.3
Sergentanis 2013	BMI 25-29.99 vs. < 25	Melanoma inc, men	C43	6	1.41 (1.09-1.83)	35	103.7	1.26 (0.99-1.61)	0	16.7
Sergentanis 2013	BMI 30+ vs. < 25	Melanoma inc, men	C43	7	1.31 (1.10-1.57)	26	319.8	1.24 (0.98-1.57)	0	13.8
Sergentanis 2013	BMI, 25+ vs. <25, ASE	Melanoma inc, men	C43	2	1.74 (0.85-3.56)	0	6.8	1.69 (0.78-3.65)	0	4.5
Renehan 2008	BMI per 5kg/m^2 increase	Melanoma inc, men	C43	6	1.17 (1.05-1.30)	46	200.5	1.11 (0.96-1.27)	2	5.4
Sergentanis 2013	BMI 25-29.99 vs. < 25	Melanoma inc, women	C43	7	0.97 (0.87-1.08)	22	0.9	1.00 (0.92-1.08)	0	0.08

Sergentanis 2013	BMI 30+ vs. < 25	Melanoma inc, women	C43	6	0.87 (0.70-1.08)	37	3.8	0.91 (0.81-1.03)	0	6.8
Sergentanis 2013	BMI, 25+ vs. <25, ASE	Melanoma inc, women	C43	5	0.80 (0.65-0.99)	0	23.4	0.84 (0.67-1.07)	0	5.5
Renehan 2008	BMI per 5kg/m^2 increase	Melanoma inc, women	C43	5	0.96 (0.93-1.00)	0	31.2	0.97 (0.93-1.01)	0	6.5
Yang 2015	BS; surgery vs. no surgery	Breast CA inc	C50	2	0.42 (0.07-2.31)	97	2.2	0.70 (0.18-2.71)	37	0.7
Suzuki 2009	Obesity±, high vs. low*	Breast CA inc, ER- PR-, PoMP	C50	4	0.89 (0.53-1.51)	61	0.5	0.87 (0.56-1.35)	21	0.8
Suzuki 2009	Obesity±, high vs. low*	Breast CA inc, ER- PR+, PoMP	C50	2	2.03 (1.04-3.95)	61	25.9	1.73 (0.77-3.85)	0	4.5
Suzuki 2009	Obesity±, high vs. low*	Breast CA inc, ER+ PR-, PoMP	C50	2	0.64 (0.42-0.97)	27	25.9	0.72 (0.48-1.07)	0	8.6
Suzuki 2009	Obesity±, high vs. low*	Breast CA inc, ER+ PR+, PoMP	C50	4	1.74 (1.34-2.25)	69	> 1000.0	1.55 (1.09-2.21)	0	64.1
Renehan 2008	BMI per 5kg/m^2 increase	Breast CA inc, PoMP	C50	29	1.13 (1.09-1.17)	61	> 1000.0	1.06 (1.03-1.10)	0	> 1000.0
Harvie 2003	WC: lowest vs. highest cat.	Breast CA inc, PoMP	C50	4	0.75 (0.55-1.04)	73	11.2	0.84 (0.60-1.19)	33	2.1
Keum 2015	WG per 5kg increase	Breast CA inc, PoMP HRT -	C50	7	1.11 (1.09-1.13)	17	> 1000.0	1.08 (1.03-1.14)	0	313.8
Keum 2015	WG: highest vs. lowest	Breast CA inc, PoMP	C50	7	1.75 (1.53-2.00)	0	> 1000.0	1.57 (1.19-2.06)	0	711.1
Connolly 2002	WHR: highest vs lowest cat.	Breast CA inc, PoMP	C50	4	1.24 (0.92-1.67)	23	5.3	1.17 (0.91-1.51)	0	3.8
Keum 2015	WG per 5kg increase	Breast CA inc, PoMP HRT +	C50	4	1.01 (0.99-1.02)	0	0.9	1.01 (0.99-1.02)	0	0.9
Cheraghi 2012	BMI 25-29.99 vs. < 25	Breast CA inc, PoMP	C50	8	1.12 (1.06-1.18)	57	> 1000.0	1.08 (1.02-1.13)	0	201.9
Cheraghi 2012	BMI 30+ vs. < 25	Breast CA inc, PoMP	C50	8	1.16 (1.08-1.25)	65	> 1000.0	1.09 (1.02-1.16)	0	103.4
Renehan 2008	BMI per 5kg/m^2 increase	Breast CA inc, PrMP	C50	20	0.92 (0.88-0.96)	38	> 1000.0	0.95 (0.90-0.99)	0	35.7
Harvie 2003	WC: lowest vs. highest cat.	Breast CA inc, PrMP	C50	2	0.78 (0.43-1.43)	73	1.4	0.87 (0.50-1.54)	37	0.6
Keum 2015	WG per 5kg increase	Breast CA inc, PrMP	C50	3	0.99 (0.95-1.03)	35	0.9	0.99 (0.96-1.03)	4	0.4
Keum 2015	WG: highest vs. lowest cat.	Breast CA inc, PrMP	C50	4	0.99 (0.81-1.21)	17	0.1	1.01 (0.83-1.22)	10	0.05
Amadou 2013	WHR per 0.1 unit increase	Breast CA inc, PrMP	C50	3	1.02 (0.97-1.07)	0	1.1	1.02 (0.97-1.07)	0	1.1
Connolly 2002	WHR: highest vs lowest cat.	Breast CA inc, PrMP	C50	3	1.32 (0.91-1.92	34	5.8	1.21 (0.86-1.71)	0	2.7
Cheraghi 2012	BMI 25-29.99 vs. < 25	Breast CA inc, PrMP	C50	4	1.01 (0.77-1.31)	72	0.1	0.94 (0.82-1.09)	1	1.5
Cheraghi 2012	BMI 30+ vs. < 25	Breast CA inc, PrMP	C50	4	0.91 (0.70-1.18)	37	1.1	0.92 (0.79-1.08)	0	2.2

Poorolajal 2015	BMI 25-29.99 vs. < 25	Cervical CA inc	C53	2	1.10 (1.03-1.17)	0	233.2	1.08 (0.94-1.25)	0	2.8
Poorolajal 2015	BMI 30+ vs. < 25	Cervical CA inc	C53	2	1.09 (0.70-1.68)	36	0.4	1.07 (0.68-1.69)	30	0.3
Upala 2015	BS; surgery vs. no surgery	Endometrial CA inc	C54.1	3	0.39 (0.19-0.79)	80	115.2	0.55 (0.25-1.20)	0	6.4
Zhang 2014	BMI 25-29.99 vs. < 25	Endometrial CA inc	C54.1	7	1.60 (1.10-2.33)	96	75.1	1.67 (1.13-2.45)	0	101.1
Zhang 2014	BMI 30+ vs. < 25	Endometrial CA inc	C54.1	6	3.10 (2.63-3.65)	66	> 1000.0	2.99 (1.49-6.02)	0	472.8
Aune 2015	BMI iya per 5kg/m^2 increase	Endometrial CA inc	C54.1	9	1.45 (1.28-1.64)	75	> 1000.0	1.33 (1.14-1.55)	0	> 1000.0
Aune 2015	BMI per 5kg/m^2 increase	Endometrial CA inc	C54.1	28	1.54 (1.47-1.61)	81	> 1000.0	1.41 (1.26-1.57)	0	> 1000.0
Aune 2015	HC per 10cm increase	Endometrial CA inc	C54.1	2	1.29 (1.19-1.41)	0	> 1000.0	1.23 (0.98-1.54)	0	11.9
Aune 2015	WC per 10cm increase	Endometrial CA inc	C54.1	4	1.27 (1.17-1.39)	70	> 1000.0	1.19 (1.03-1.38)	0	61.2
Aune 2015	WG per 5kg increase	Endometrial CA inc	C54.1	7	1.16 (1.12-1.20)	47	> 1000.0	1.13 (1.05-1.22)	0	895.1
Aune 2015	Weight per 5kg increase	Endometrial CA inc	C54.1	7	1.17 (1.13-1.22)	62	> 1000.0	1.15 (1.06-1.25)	0	942.2
Aune 2015	WHR per 0.1 unit increase	Endometrial CA inc	C54.1	5	1.21 (1.13-1.29)	0	> 1000.0	1.16 (1.04-1.32)	0	95.1
Crosbie 2010	BMI per 5kg/m^2 increase	Endometrial CA inc: PoMP	C54.1	6	1.60 (1.40-1.83)	89	> 1000.0	1.52 (1.16-1.98)	0	462.3
Crosbie 2010	BMI per 5kg/m^2 increase	Endometrial CA inc: PoMP HRT +	C54.1	6	1.18 (1.07-1.31)	0	601.3	1.16 (1.02-1.31)	0	38.1
Crosbie 2010	BMI per 5kg/m^2 increase	Endometrial CA inc: PoMP HRT -	C54.1	3	1.90 (1.56-2.30)	77	> 1000.0	1.80 (1.06-3.07)	0	32.0
Keum 2015	WG per 5kg increase	Endometrial CA inc: PoMP, HRT+	C54.1	2	1.09 (1.02-1.17)	0	83.5	1.09 (0.99-1.20)	0	13.3
Keum 2015	WG per 5kg increase	Endometrial CA inc: PoMP, HRT -	C54.1	2	1.38 (1.28-1.49)	0	> 1000.0	1.40 (0.97-2.02)	0	13.0
Crosbie 2010	BMI per 5kg/m^2 increase	Endometrial CA inc: PrMP	C54.1	6	1.49 (1.39-1.61)	20	> 1000.0	1.36 (1.11-1.67)	0	285.2
Crosbie 2010	BMI per 5kg/m^2 increase	Endometrial CA inc: Type I	C54.1	3	1.75 (1.51-2.03)	82	> 1000.0	1.71 (1.06-2.77)	0	33.8
Crosbie 2010	BMI per 5kg/m^2 increase	Endometrial CA inc: Type II	C54.1	3	1.59 (1.29-1.78)	76	> 1000.0	1.47 (1.03-2.08)	0	30.6
Aune 2015	BMI per 5kg/m^2 increase	Endometrial CA mort	C54.1	3	1.46 (1.29-1.65)	29	> 1000.0	1.35 (0.90-2.05)	0	6.0
Aune 2015	BMI iya per 5kg/m^2 increase	Ovarian CA inc	C56	6	1.12 (1.05-1.19)	0	> 1000.0	1.10 (1.01-1.19)	0	33.2
Aune 2015	BMI per 5kg/m^2 increase	Ovarian CA inc	C56	24	1.08 (1.04-1.12)	48	> 1000.0	1.03 (1.00-1.06)	0	17.5
Poorolajal 2014	BMI 25-29.99 vs. < 25	Ovarian CA inc	C56	13	1.08 (0.97-1.19)	49	4.8	1.02 (0.93-1.11)	0	0.4

Poorolajal 2014	BMI 30+ vs. < 25	Ovarian CA inc	C56	13	1.27 (1.17-1.38)	12	> 1000.0	1.16 (1.02-1.30)	0	51.9
Aune 2015	HC per 10cm increase	Ovarian CA inc	C56	4	1.04 (0.82-1.31)	72	0.3	1.09 (0.91-1.29)	17	1.8
Aune 2015	WC per 10cm increase	Ovarian CA inc	C56	6	1.06 (1.00-1.12)	0	18.1	1.05 (0.99-1.11)	0	7.3
Aune 2015	WG per 5kg increase	Ovarian CA inc	C56	6	1.02 (0.96-1.09)	67	1.0	1.02 (0.98-1.06)	0	2.9
Aune 2015	Weight per 5kg increase	Ovarian CA inc	C56	4	1.03 (1.01-1.05)	7	715.3	1.03 (1.00-1.05)	0	23.2
Aune 2015	WHR per 0.1 unit increase	Ovarian CA inc	C56	5	1.00 (0.93-1.07)	0	0.1	0.99 (0.93-1.07)	0	0.1
Poorolajal 2014	BMI 25-29.99 vs. < 25	Ovarian CA inc, PoMP	C56	5	1.03 (0.87-1.21)	52	0.4	0.99 (0.86-1.15)	23	0.1
Poorolajal 2014	BMI 30+ vs. < 25	Ovarian CA inc, PoMP	C56	5	1.23 (1.03-1.47)	46	37.6	1.12 (0.97-1.29)	0	6.7
Keum 2015	WG per 5kg increase	Ovarian CA inc, PoMP, HRT -	C54.1	2	1.13 (1.03-1.24)	0	116.4	1.11 (0.99-1.25)	0	10.6
Poorolajal 2014	BMI 25-29.99 vs. < 25	Ovarian CA inc, PrMP	C56	3	1.21 (0.96-1.53)	0 0	7.9	1.14 (0.85-1.53)	0	1.6
Poorolajal 2014	BMI 30+ vs. < 25	Ovarian CA inc, PrMP	C56	3	1.57 (1.20-2.06)	0	> 1000.0	1.54 (0.95-2.51)	0	11.4
Zhang 2015	BMI obese vs. normal weight*	Prostate CA inc	C61	14	1.00 (0.95-1.06)	40	0.1	0.99 (0.95-1.03)	1	0.6
Renehan 2008	BMI per 5kg/m^2 increase	Prostate CA inc	C61	26	1.03 (0.99-1.06)	76	8.5	1.00 (0.97-.03)	19	0.02
MacInnis 2006	WC per 10cm increase	Prostate CA inc	C61	4	1.03 (0.97-1.09)	0	2.0	1.03 (0.97-1.09)	0	2.0
Keum 2015	WG per 5kg increase	Prostate CA inc	C61	4	0.98 (0.94-1.02)	51	3.3	0.99 (0.96-1.02)	13	0.8
Keum 2015	WG: highest vs. lowest cat.	Prostate CA inc	C61	8	0.98 (0.91-1.06)	31	0.7	1.01 (0.95-1.07)	0	0.3
Discaccati 2012	BMI per 5kg/m^2 increase	Prostate CA inc, advanced	C61	13	1.09 (1.02-1.16)	40	98.0	1.04 (0.99-1.09)	0	10.6
Discaccati 2012	BMI per 5kg/m^2 increase	Prostate CA inc, localized	C61	12	0.94 (0.91-0.97)	20	> 1000.0	0.95 (0.91-0.99)	0	76.7
Keum 2015	WG per 5kg increase	Prostate CA inc, advanced	C61	4	1.04 (0.99-1.09)	0	7.6	1.02 (0.96-1.10)	0	1.1
Keum 2015	WG per 5kg increase	Prostate CA inc, high screening rate	C61	2	0.95 (0.92-0.99)	0	141.4	0.95 (0.90-1.01)	0	11.4
Keum 2015	WG per 5kg increase	Prostate CA inc, localised	C61	4	0.96 (0.92-1.00)	35	12.2	0.98 (0.94-1.01)	0	5.8
Keum 2015	WG per 5kg increase	Prostate CA inc, low screening rate	C61	2	1.01 (0.97-1.05)	0	0.5	1.01 (0.97-1.05)	0	0.5
Zhang 2015	BMI obese vs. normal weight*	Prostate CA mort	C61	10	1.24 (1.15-1.33)	0	> 1000.0	1.22 (1.09-1.37)	0	> 1000.0
Cao 2011	BMI per 5kg/m^2 increase	Prostate CA mort	C61	6	1.15 (1.06-1.25)	59	> 1000.0	1.10 (1.02-1.18)	0	51.4

Yang 2015	BS; surgery vs. no surgery	Kidney CA inc	C64	2	1.13 (0.52-2.45)	0	0.3	1.13 (0.52-2.45)	0	0.3
Wang 2014	BMI 25-29.99 vs. < 25	Kidney CA inc	C64	43	1.35 (1.27-1.43)	36	> 1000.0	1.25 (1.16-1.35)	0	> 1000.0
Wang 2014	BMI 30+ vs. < 25	Kidney CA inc	C64	31	1.79 (1.64-1.95)	44	> 1000.0	1.52 (1.32-1.75)	0	> 1000.0
Renehan 2008	BMI per 5kg/m^2 increase	Kidney CA inc	C64	19	1.30 (1.23-1.36)	49	> 1000.0	1.23 (1.12-1.34)	0	> 1000.0
Keum 2015	WG: highest vs. lowest cat.	Kidney CA inc	C64	3	1.42 (1.11-1.81)	10	210.1	1.28 (0.97-1.69)	0	10.5
Renehan 2008	BMI per 5kg/m^2 increase	Kidney CA inc, men	C64	9	1.24 (1.17-1.32)	22	> 1000.0	1.19 (1.06-1.33)	0	329.3
Renehan 2008	BMI per 5kg/m^2 increase	Kidney CA inc, women	C64	10	1.33 (1.25-1.42)	45	> 1000.0	1.28 (1.12-1.46)	0	> 1000.0
Sun 2015	BMI 25-29.99 vs. < 25	Bladder CA inc	C67	26	1.07 (1.01-1.14)	38	38.0	1.04 (0.99-1.08)	43	6.8
Sun 2015	BMI 30+ vs. < 25	Bladder CA inc	C67	26	1.09 (1.04-1.15)	15	> 1000.0	1.06 (1.00-1.12)	0	28.0
Sun 2015	BMI per 5kg/m^2 increase	Bladder CA inc	C67	8	1.04 (1.00-1.09)	32	13.6	1.04 (1.00-1.08)	0	16.4
Shao 2014	BMI 25-29.99 vs. < 25	Meningeoma inc	C70	4	1.11 (0.94-1.30)	0	3.5	1.08 (0.91-1.28)	0	1.6
Shao 2014	BMI 30+ vs. < 25	Meningeoma inc	C70	4	1.55 (1.28-1.86)	0	> 1000.0	1.49 (1.09-2.04)	0	75.6
Ma 2015	BMI 30+ vs. < 25	Thyroid CA inc	C73	24	1.29 (1.20-1.37)	21	> 1000.0	1.22 (1.12-1.33)	0	> 1000.0
Renehan 2008	BMI per 5kg/m^2 increase	Thyroid CA inc	C73	7	1.23 (1.10-1.39)	62	> 1000.0	1.13 (1.03-1.24)	0	134.9
Keum 2015	WG: highest vs. lowest cat.	Thyroid CA inc	C73	4	1.06 (0.82-1.38)	0	0.6	1.03 (0.79-1.35)	0	0.2
Renehan 2008	BMI per 5kg/m^2 increase	Thyroid CA inc, men	C73	4	1.32 (1.04-1.69)	77	39.3	1.12 (1.00-1.25)	0	12.2
Renehan 2008	BMI per 5kg/m^2 increase	Thyroid CA inc, women	C73	3	1.14 (1.06-1.22)	3	> 1000.0	1.15 (0.99-1.33)	0	15.3
Larsson 2011	BMI 25-29.99 vs. < 25	Hodgkin's lymphoma inc	C81	7	0.97 (0.85-1.12)	17	0.4	0.96 (0.85-1.08)	0	1.1
Larsson 2011	BMI 30+ vs. < 25	Hodgkin's lymphoma inc	C81	4	1.41 (1.14-1.75)	22	534.2	1.24 (0.96-1.59)	0	9.4
Yang 2015	BS; surgery vs. no surgery	NHL inc	C82-C85	2	0.57 (0.25-1.29)	0	4.5	0.57 (0.25-1.29)	0	4.5
Larsson 2007	BMI 25-29.99 vs. < 25	NHL inc	C82-C85	15	1.06 (0.99-1.12)	28	12.3	1.03 (0.99-1.07)	0	5.7
Larsson 2007	BMI 30+ vs. < 25	NHL inc	C82-C85	15	1.19 (1.04-1.37)	66	67.7	1.09 (0.99-1.19)	0	13.1
Larsson 2011	BMI per 5kg/m^2 increase	NHL inc	C82-C85	23	1.07 (1.04-1.10)	9	> 1000.0	1.05 (1.02-1.09)	0	772.7
Larsson 2011	BMI per 5kg/m^2 increase	NHL mort	C82-C85	7	1.14 (1.04-1.25)	53	188.6	1.10 (1.01-1.20)	0	33.2

Castillo 2014	BMI 25-29.99 vs. < 25	Diff. large B-cell lymphoma inc	C83.3	10	1.13 (1.02-1.25)	0	52.5	1.10 (0.98-1.23)	0	9.3
Castillo 2014	BMI 30+ vs. < 25	Diff. large B-cell lymphoma inc	C83.3	12	1.25 (1.08-1.44)	27	390.6	1.15 (1.00-1.32)	0	18.4
Yang 2015	BS; surgery vs. no surgery	Multiple myeloma inc	C90.0	2	0.61 (0.14-2.63)	0	1.0	0.61 (0.14-2.63)	0	1.0
Wallin 2012	BMI 25-29.99 vs. < 25	Multiple myeloma inc	C90.0	20	1.12 (1.07-1.18)	0	> 1000.0	1.10 (1.02-1.18)	0	85.2
Wallin 2012	BMI 30+ vs. < 25	Multiple myeloma inc	C90.0	19	1.21 (1.08-1.35)	34	978.8	1.14 (1.01-1.28)	0	32.5
Renehan 2008	BMI per 5kg/m^2 increase	Multiple myeloma inc	C90.0	13	1.12 (1.09-1.15)	0	> 1000.0	1.09 (1.03-1.17)	0	171.3
Renehan 2008	BMI per 5kg/m^2 increase	Multiple myeloma inc, men	C90.0	7	1.12 (1.06-1.18)	5	> 1000.0	1.07 (0.98-1.17)	0	7.3
Renehan 2008	BMI per 5kg/m^2 increase	Multiple myeloma inc, women	C90.0	6	1.11 (1.08-1.15)	0	> 1000.0	1.12 (1.02-1.23)	0	58.0
Wallin 2012	BMI 25-29.99 vs. < 25	Multiple myeloma mort	C90.0	8	1.15 (1.04-1.26)	0	168.5	1.12 (0.99-1.27)	0	13.8
Wallin 2012	BMI 30+ vs. < 25	Multiple myeloma mort	C90.0	7	1.54 (1.35-1.76)	0	> 1000.0	1.50 (1.12-2.02)	0	147.1
Castillo 2012	BMI 30+ vs. < 25	Leukemia inc	C91-C92	25	1.26 (1.16-1.37)	43	> 1000.0	1.17 (1.07-1.27)	0	> 1000.0
Castillo 2012	BMI 30+ vs. < 25	Leukemia mort	C91-C92	8	1.28 (1.11-1.49)	30	> 1000.0	1.18 (1.02-1.37)	0	32.3
Renehan 2008	BMI per 5kg/m^2 increase	Leukemia inc	C91-C95	8	1.12 (1.05-1.18)	51	> 1000.0	1.07 (1.01-1.14)	0	52.0
Renehan 2008	BMI per 5kg/m^2 increase	Leukemia inc, men	C91-C95	5	1.08 (1.02-1.13)	0	118.0	1.06 (0.98-1.14)	0	6.7
Renehan 2008	BMI per 5kg/m^2 increase	Leukemia inc, women	C91-C95	3	1.17 (1.04-1.33)	79	100.0	1.10 (1.00-1.21)	0	15.7
Larsson 2008	BMI 30+ vs. < 25	Leukaemia inc, ALL	C91	5	1.65 (1.16-2.35)	7	195.6	1.40 (0.96-2.04)	0	11.3
Larsson 2008	BMI 30+ vs. < 25	Leukaemia inc, CLL	C91	5	1.25 (1.11-1.41)	31	> 1000.0	1.17 (1.02-1.34)	0	45.8
Larsson 2008	BMI 30+ vs. < 25	Leukaemia inc, AML	C92	6	1.52 (1.19-1.95)	79	> 1000.0	1.20 (1.03-1.40)	0	45.2
Larsson 2008	BMI 30+ vs. < 25	Leukaemia inc, CML	C92	5	1.26 (1.09-1.46)	0	592.7	1.20 (1.03-1.41)	0	45.3

Abbreviations: AdCa, adenocarcinoma; adv, advanced; ALL, acute lymphatic leukemia; AML, acute myeloic leukemia; ASE, adjusted for sunlight exposure; biliary tract system cancer, includes cancers of gallbladder, extrahepatic bile duct and ampulla of Vateri; BMI, Body Mass Index; BMI iya, Body Mass Index in young adulthood; BS, bariatric surgery; CA, cancer; CI, confidence interval; CLL, chronic lymphatic leukemia; CML, chronic myeloic leukemia; ER = estrogen receptor status; HC, hip circumference; HRT, hormone replacement therapy; inc, incidence; loc, localised; ; LR: Likelihood Ratio for the corresponding credibility ceiling; mort, mortality; NHL, Non-Hodgkin lymphoma; PoMP, postmenopausal; PR = progesterone receptor status; PrMP, premenopausal; RR, relative risk; SqCa, squamous cell carcinoma; WC, waist circumference; WG, weight gain; WHR, waist-to-hip ratio;

*cut-offs in categories varied between included studies

± BMI, weight gain or weight change, varies between included studies

All statistical tests were two-sided

Supplementary Table 5: Details of evidence grading for meta-analyses associating continuous contrasts of obesity and risk of cancer incidence or mortality — only cohort studies included.

Obesity indices	Exposure contrast	Outcome	N *	Sample size Cases/Cohort	Largest study#	Random effects Summary RR (95% CI)†	Random P-value¶	95% Prediction interval	10% credibility RR (95% CI)	Egger's P‡	I^2 (%)	Excess significance§	
												O/E**	P-value ||
**Associations supported by strong evidence**													
BMI	per 5kg/m^2 increase	Oesophageal AdCa inc	8	1,918 / 6,430,994	1.67 (1.44-1.92)	1.53 (1.41-1.67)	1.4E-22	1.38-1.71	1.36 (1.12-1.65)	0.19	0	6/6.5	NP
BMI	per 5kg/m^2 increase	Colon CA inc, men	24	42,821 / 5,213,139	1.27 (1.23-1.31)	1.30 (1.25-1.35)	<1E-100	1.19-1.42	1.24 (1.14-1.34)	0.30	24	18/15.4	0.39
BMI	per 5kg/m^2 increase	Rectal CA inc, men	17	23,167 / 4,293,489	1.08 (1.05-1.12)	1.09 (1.06-1.13)	1.1E-7	1.04-1.15	1.08 (1.02-1.13)	0.66	6	3/3.2	NP
BMI	per 5kg/m^2 increase	Biliary tract system CA inc	10	6,961 / 6,008,270	1.88 (1.60-2.21)	1.56 (1.34-1.81)	1.3E-8	1.13-2.15	1.38 (1.13-1.68)	0.24	25	4/9.00	NP
BMI	per 5kg/m^2 increase	Pancreatic CA inc	22	8,987 / 4,978,230	1.09 (1.03-1.16)	1.10 (1.06-1.14)	2.2E-7	1.01-1.21	1.06 (1.02-1.10)	0.34	25	4/3.4	0.77

WG	per 5kg increase	Breast CA inc, PoMP, HRT-	7	10,283 / 342,249	1.14 (1.11-1.18)	1.11 (1.09-1.13)	5.7E-23	1.07-1.15	1.08 (1.03-1.14)	0.66	17	5/4.5	1.00
WHR	per 0.1 unit increase	Endometrial CA inc	5	2,447 / 394,340	1.33 (1.18-1.51)	1.21 (1.13-1.29)	1.0E-8	1.09-1.34	1.16 (1.04-1.32)	0.54	0	3/4.4	NP
BMI	per 5kg/m^2 increase	Endometrial CA inc, PrMP	6	5,981 / 2,558,935	1.53 (1.48-1.58)	1.49 (1.39-1.61)	3.1E-27	1.27-1.76	1.36 (1.11-1.67)	0.56	20	5/4.2	0.67
BMI	per 5kg/m^2 increase	Kidney CA inc, men	9	8,983 / 3,945,950	1.19 (1.13-1.24)	1.24 (1.17-1.32)	5.9E-12	1.10-1.41	1.19 (1.06-1.33)	0.16	22	5/4.0	0.52
BMI	per 5kg/m^2 increase	Kidney CA inc, women	10	8,795 / 4,252,718	1.35 (1.28-1.42)	1.33 (1.25-1.42)	7.2E-18	1.13-1.57	1.28 (1.12-1.46)	0.39	45	9/6.9	0.19
BMI	per 5kg/m^2 increase	Multiple myeloma inc	13	14,634 / 7,429,478	1.11 (1.07-1.15)	1.12 (1.09-1.15)	2.8E-15	1.08-1.15	1.09 (1.03-1.17)	0.65	0	4/3.5	0.76
BMI	per 5kg/m^2 increase	Multiple myeloma inc, women	6	7,183 / 3,607,950	1.11 (1.07-1.15)	1.11 (1.08-1.15)	2.8E-10	1.06-1.17	1.12 (1.02-1.23)	0.82	0	2/1.7	0.67

Associations supported by highly suggestive evidence

BMI	per 5kg/m^2 increase	Oesophageal SqCa inc	5	3,545 / 6,369,699	0.77 (0.70-0.85)	0.63 (0.53-0.75)	1.7E-7	0.34-1.15	0.68 (0.52-0.91)	0.26	80	5/3.5	0.33
BMI	per 5kg/m^2 increase	Oesophageal SqCa inc, women	2	1,758 / 3,224,247	0.61 (0.54-0.70)	0.57 (0.48-0.67)	1.2E-10	NA	0.57 (0.31-1.05)	NA	58	2/2.0	1.00
BMI	per 5kg/m^2 increase	Colon CA inc	44	82,766 / 9,434,650	1.03 (1.01-1.05)	1.22 (1.17-1.28)	3.9E-16	0.95-1.57	1.08 (1.05-1.11)	<0.01	84	27/4.4	<0.01
WC	per 10cm increase	Colon CA inc	8	3,371 / 869,185	1.14 (1.05-1.25)	1.25 (1.15-1.35)	3.0E-8	1.01-1.54	1.18 (1.07-1.31)	0.06	50	7/2.4	<0.01
BMI	per 5kg/m^2 increase	Liver CA inc	19	17,281 / 4,977,849	1.12 (1.05-1.19)	1.37 (1.23-1.53)	2.4E-8	0.90-2.07	1.12 (1.04-1.20)	0.05	81	10/4.7	0.01
BMI	per 5kg/m^2 increase	Lung CA inc	15	12,591 / 2,833,306	0.86 (0.82-0.90)	0.77 (0.72-0.83)	8.0E-13	0.61-0.97	0.84 (0.78-0.92)	0.05	71	10/7.1	0.19
BMI	per 5kg/m^2 increase	Lung CA inc, men	10	7,569 / 1,387,620	0.84 (0.79-0.88)	0.79 (0.73-0.85)	1.7E-10	0.65-0.95	0.83 (0.75-0.92)	0.07	53	7/5.0	0.23
BMI	per 5kg/m^2 increase	Breast CA inc, PoMP	29	27,700 / 2,318,992	1.18 (1.15-1.22)	1.13 (1.09-1.17)	4.1E-10	0.97-1.31	1.06 (1.03-1.10)	0.67	61	12/16.6	NP
BMI iya	per 5kg/m^2 increase	Endometrial CA inc	9	4,345 / 631,915	1.23 (1.11-1.35)	1.45 (1.28-1.64)	1.9E-9	0.98-2.15	1.33 (1.14-1.55)	0.41	75	8/5.2	0.09
BMI	per 5kg/m^2 increase	Endometrial CA inc	28	22,320 / 6,445,255	1.65 (1.60-1.71)	1.54 (1.47-1.61)	<1.0E-100	1.26-1.89	1.41 (1.26-1.57)	0.35	81	26/25.2	1.00
WC	per 10cm increase	Endometrial CA inc	4	1,524 / 315,770	1.28 (1.19-1.37)	1.27 (1.17-1.39)	7.3E-8	0.88-1.85	1.19 (1.03-1.38)	0.59	70	3/2.9	1.00
WG	per 5kg increase	Endometrial CA inc	7	2,806 / 460,901	1.17 (1.12-1.22)	1.16 (1.12-1.20)	3.7E-18	1.06-1.27	1.13 (1.05-1.22)	0.95	47	6/2.8	0.02
Weight	per 5kg increase	Endometrial CA inc	7	1,778 / 342,382	1.11 (1.08-1.15)	1.17 (1.13-1.22)	7.7E-15	1.04-1.31	1.15 (1.06-1.25)	0.29	62	6/1.0	<0.01
BMI	per 5kg/m^2 increase	Endometrial CA inc, PoMP	6	10,075 / 2,558,935	1.51 (1.45-1.58)	1.60 (1.40-1.83)	1.4E-11	1.01-2.53	1.52 (1.16-1.98)	0.88	89	6/5.0	0.60
BMI	per 5kg/m^2 increase	Endometrial CA inc, Type I	3	7,125 / 1,102,927	1.58 (1.53-1.62)	1.75 (1.51-2.03)	1.8E-13	0.30-10.24	1.71 (1.06-2.77)	0.26	82	3/2.9	1.00
BMI	per 5kg/m^2 increase	Endometrial CA inc, Type II	3	1,059 / 1,102,927	1.35 (1.25-1.46)	1.59 (1.29-1.78)	5.7E-7	0.24-9.67	1.47 (1.03-2.08)	0.52	76	3/1.5	0.25
BMI	per 5kg/m^2 increase	Kidney CA inc	19	17,778 / 8,198,668	1.19 (1.13-1.24)	1.30 (1.23-1.36)	9.8E-25	1.12-1.50	1.23 (1.12-1.34)	0.11	49	14/7.9	<0.01

Associations supported by suggestive evidence

BMI	per 5kg/m^2 increase	Oesophageal SqCa inc, men	3	1,787 / 3,145,452	0.77 (0.70-0.85)	0.71 (0.61-0.84)	6.1E-5	0.14-3.69	0.72 (0.52-0.98)	0.03	44	3/1.8	0.28
BMI	per 5kg/m^2 increase	Colon CA inc, women	20	39,945 / 4,221,511	1.03 (1.01-1.05)	1.12 (1.06-1.17)	6.9E-6	0.96-1.30	1.05 (1.02-1.09)	<0.01	59	9/2.1	<0.01
WC	per 10cm increase	Colon CA inc, men	5	1,869 / 469,320	1.15 (1.01-1.31)	1.33 (1.18-1.50)	1.6E-6	0.95-1.86	1.23 (1.04-1.44)	0.51	45	4/1.5	0.03
WC	per 10cm increase	Colon CA inc, women	3	1,502 / 399,865	1.14 (1.05-1.25)	1.16 (1.08-1.23)	1.1E-5	0.76-1.76	1.16 (1.01-1.32)	0.25	0	3/1.0	0.04
WHR	per 0.1 unit increase	Colon CA inc	8	3,132 / 941,955	1.24 (1.10-1.39)	1.29 (1.17-1.43)	1.1E-6	0.97-1.72	1.17 (1.05-1.31)	0.16	53	6/4.2	0.29
WHR	per 0.1 unit increase	Colon CA inc, men	4	1,563 / 461,754	1.24 (1.05-1.46)	1.43 (1.19-1.71)	1.0E-6	0.71-2.87	1.33 (1.05-1.68)	0.32	56	4/2.1	0.13
WHR	per 0.1 unit increase	Colon CA inc, women	4	1,569 / 480,201	1.24 (1.10-1.39)	1.20 (1.08-1.33)	5.0E-5	0.86-1.69	1.12 (0.98-1.28)	0.90	30	2/2.1	NP
WG	per 5kg increase	Colon CA inc, men	4	1,718 / 142,085	1.10 (1.03-1.17)	1.09 (1.05-1.14)	4.1E-5	1.00-1.19	1.07 (1.00-1.14)	0.56	0	2/0.8	0.12
WG	per 5kg increase	Colon CA inc	7	2.909 / 298,174	1.10 (1.03-1.17)	1.07 (1.03-1.10)	8.3E-5	1.02-1.11	1.04 (1.00-1.09)	0.88	0	2/1.3	0.50
WG	per 1kg increase / year	Colorectal CA inc	17	27,638 / 1,974,918	1.31 (1.18-1.45)	1.20 (1.10-1.31)	3.9E-5	0.95-1.52	1.11 (1.03-1.21)	0.72	37	4/15.3	NP

BMI	per 5kg/m^2 increase	Rectal CA inc	31	43,196 / 7,495,868	1.01 (0.98-1.04)	1.07 (1.03-1.10)	5.9E-5	0.98-1.16	1.03 (1.00-1.05)	0.35	27	4/1.7	0.09
WC	per 10cm increase	Pancreatic CA inc	5	1,365 / 1,154,996	1.08 (0.98-1.18)	1.11 (1.05-1.18)	2.0E-4	1.02-1.22	1.11 (1.02-1.20)	0.11	0	2/0.5	0.09
WHR	per 0.1 unit increase	Pancreatic CA inc	4	1,438 / 1,109,945	1.32 (1.12-1.56)	1.20 (1.09-1.31)	9.6E-5	0.96-1.50	1.14 (1.01-1.28)	0.54	7	2/3.0	NP
BMI	per 5kg/m^2 increase	Lung CA inc, smokers	4	6,185 / 475,940	0.82 (0.77-0.87)	0.77 (0.69-0.86)	7.8E-6	0.48-1.24	0.81 (0.68-0.97)	0.12	78	4/2.9	0.58
BMI	per 5kg/m^2 increase	Breast CA inc, PrMP	20	20,820 / 2,200,787	0.91 (0.86-0.97)	0.92 (0.88-0.97)	4.6E-4	0.81-1.05	0.95 (0.90-0.99)	0.28	38	7/6.3	0.81
BMI	per 5kg/m^2 increase	Ovarian CA inc	24	17,734 / 16,343,135	0.97 (0.93-1.01)	1.08 (1.04-1.12)	8.6E-5	0.96-1.21	1.03 (1.00-1.06)	0.07	48	6/1.8	<0.01
BMI iya	per 5kg/m^2 increase	Ovarian CA inc	6	9,452 / 11,085,425	1.16 (1.04-1.29)	1.12 (1.05-1.19)	3.8E-4	1.03-1.23	1.10 (1.01-1.19)	0.60	0	1/2.5	NP
BMI	per 5kg/m^2 increase	Prostate CA inc, loc	12	19,130 / 1,033,009	0.96 (0.93-0.98)	0.94 (0.91-0.97)	4.6E-4	0.88-1.01	0.95 (0.91-0.99)	0.46	20	3/1.7	0.40
BMI	per 5kg/m^2 increase	Prostate CA mort	6	6,817 / 1,263,483	1.08 (1.04-1.12)	1.15 (1.06-1.25)	8.3E-4	0.92-1.44	1.10 (1.02-1.18)	0.30	59	4/1.6	0.049
BMI	per 5kg/m^2 increase	Thyroid CA inc	7	6,716 / 5,316,380	1.12 (1.05-1.19)	1.23 (1.10-1.39)	4.3E-4	0.89-1.70	1.13 (1.03-1.24)	0.09	62	4/2.4	0.24
BMI	per 5kg/m^2 increase	Thyroid CA inc, women	3	3,190 / 2,159,238	1.12 (1.05-1.19)	1.14 (1.06-1.22)	4.0E-4	0.67-1.93	1.15 (0.99-1.33)	0.41	3	2/1.1	0.30
BMI	per 5kg/m^2 increase	NHL inc	23	29,064 / 9,056,494	1.04 (0.99-1.09)	1.07 (1.04-1.10)	1.9E-6	1.02-1.12	1.05 (1.02-1.09)	0.26	9	5/2.7	0.18
BMI	per 5kg/m^2 increase	Multiple myeloma inc, men	7	7,451 / 3,821,528	1.13 (1.07-1.19)	1.12 (1.06-1.18)	1.1E-4	1.02-1.22	1.07 (0.98-1.17)	0.76	5	2/2.0	NP
BMI	per 5kg/m^2 increase	Leukemia inc	8	15,117 / 6,758,260	1.07 (1.02-1.12)	1.12 (1.05-1.18)	2.3E-4	0.96-1.30	1.07 (1.01-1.14)	0.22	51	4/2.4	0.25

Associations supported by weak evidence

BMI	per 5kg/m^2 increase	Oesophageal AdCa inc, men	5	996 / 3,196,747	1.67 (1.44-1.92)	1.52 (1.33-1.74)	1.3E-9	1.09-2.11	1.31 (1.04-1.64)	0.37	24	4/3.6	1.00
BMI	per 5kg/m^2 increase	Oesophageal AdCa inc, women	3	922 / 3234247	1.54 (1.26-1.89)	1.51 (1.30-1.75)	2.8E-8	0.59-3.86	1.49 (1.05-2.12)	0.44	0	2/2.7	NP
BMI	per 5kg/m^2 increase	Lung CA inc, women	5	5,022 / 1,445,686	0.86 (0.82-0.90)	0.76 (0.61-0.94	0.01	0.36-1.63	0.88 (0.75-1.03)	0.46	86	3/3.0	1.00
BMI	per 5kg/m^2 increase	Melanoma inc, men	6	6,647 / 2,808,095	1.16 (1.08-1.26)	1.17 (1.05-1.30)	4.9E-3	0.89-1.52	1.11 (0.96-1.27)	0.89	46	3/2.5	0.69
BMI	per 5kg/m^2 increase	Melanoma inc, women	5	7,148 / 2,886,890	0.97 (0.91-1.00)	0.96 (0.93-1.00)	0.03	0.90-1.02	0.97 (0.93-1.01)	0.28	0	1/0.5	0.40
BMI	per 5kg/m^2 increase	Endometrial CA inc: PostMP, HRT-	3	699 / 360,326	1.61 (1.41-1.85)	1.90 (1.56-2.30)	9.2E-11	0.19-18.56	1.80 (1.06-3.07)	<0.01	77	3/2.9	1.00
BMI	per 5kg/m^2 increase	Endometrial CA inc: PostMP, HRT+	6	791 / 679,271	1.25 (1.05-1.47)	1.18 (1.07-1.31)	1.7E-3	1.02-1.37	1.16 (1.02-1.31)	0.77	0	1/1.7	NP
WG	per 5kg increase	Endometrial CA inc: PostMP, HRT-	2	285 / 33,340	1.36 (1.25-1.46)	1.38 (1.28-1.49)	3.6E-17	NA	1.40 (0.97-2.02)	NA	0	2/1.0	0.50
WG	per 5kg increase	Endometrial CA inc: PostMP, HRT+	2	334 / 35,333	1.09 (1.00-1.18)	1.09 (1.02-1.17)	0.01	NA	1.09 (0.99-1.20)	NA	0	1/0.2	0.18
HC	per 10 cm increase	Endometrial CA inc	2	831 / 255,650	1.32 (1.20-1.45)	1.29 (1.19-1.41)	2.9E-09	NA	1.23 (0.98-1.54)	NA	0	1/1.7	NP
BMI	per 5kg/m^2 increase	Endometrial CA mort	3	962 / 1,781,648	1.42 (1.33-1.63)	1.46 (1.29-1.65)	1.7E-9	0.48-4.48	1.35 (0.90-2.05)	0.64	29	2/2.0	1.00
Weight	per 5kg increase	Ovarian CA inc	4	1,281 / 405,655	1.02 (1.00-1.05)	1.03 (1.01-1.05)	1.3E-3	0.98-1.08	1.03 (1.00-1.05)	0.42	7	1/0.2	0.20
WG	per 5kg increase	Ovarian CA inc: PostMP, HRT-	2	217 / 23,984	1.16 (1.03-1.31)	1.13 (1.03-1.24)	8.5E-3	NA	1.11 (0.99-1.25)	NA	0	1/0.3	0.25
BMI	per 5kg/m^2 increase	Prostate CA inc, adv	13	7,067 / 1,080,790	1.00 (0.94-1.08)	1.09 (1.02-1.16)	0.01	0.92-1.29	1.04 (0.99-1.09)	0.02	40	3/0.7	0.02
WG	per 5kg increase	Prostate CA inc, high screening rate	2	4,894 / 67,335	0.96 (0.92-1.00)	0.95 (0.92-0.99)	7.0E-3	NA	0.95 (0.90-1.01)	NA	0	1/0.4	0.25
BMI	per 5kg/m^2 increase	Thyroid CA inc, men	4	3,526 / 3,157,142	1.09 (0.98-1.22)	1.32 (1.04-1.69)	0.02	0.46-3.84	1.12 (1.00-1.25)	0.23	77	2/1.0	0.27
BMI	per 5kg/m^2 increase	NHL mort	7	5,920 / 3,511,893	1.18 (1.09-1.29)	1.14 (1.04-1.25)	5.3E-3	0.90-1.45	1.10 (1.01-1.20)	0.57	53	3/3.4	NP
BMI	per 5kg/m^2 increase	Leukemia inc, men	5	7,827 / 3,497,392	1.09 (1.02-1.17)	1.08 (1.02-1.13)	8.4E-3	0.99-1.17	1.06 (0.98-1.14)	0.47	0	1/1.5	NP
BMI	per 5kg/m^2 increase	Leukemia inc, women	3	7,290 / 3,260,868	1.07 (1.02-1.12)	1.17 (1.04-1.33)	9.8E-3	0.28-4.93	1.10 (1.00-1.21)	0.20	79	3/1.1	0.05

Abbreviations: AdCa, adenocarcinoma ;adv, advanced; BMI, Body Mass Index; biliary tract system cancer, includes cancers of gallbladder, extrahepatic bile duct and ampulla of Vateri; BMI iya, Body Mass Index in young adulthood; CA, cancer; CI, confidence interval; HC, hip circumference; HRT, hormone replacement therapy; inc, incidence; mort, mortality; NA: Not available, due to <3 included studies; NHL, Non-Hodgkin lymphoma; NP: Not Pertinent, because the estimated is larger than the observed, and there is no evidence of excess statistical significance based on the assumption made for the plausible effect size; SqCa, squamous cell carcinoma; PrMP, premenopausal; PoMP, postmenopausal; RR, relative risk; WC, waist circumference; WG, weight gain; WHR, waist-to-hip ratio.
* Number of studies
# Relative risk and 95% confidence interval of largest study (smallest SE) in each meta-analysis.
† Random effects refer to summary risk ratio (95% CI) using the random-effects model.
¶ P value of summary random effects estimate.
‡ P-value from the Egger's regression asymmetry test.
§ Expected number of statistically significant studies using the point estimate of the largest study (smallest standard error) as the plausible effect size.
** Observed/Expected number of statistically significant studies
|| P value of the excess statistical significance test.
All statistical tests were two-sided.

Supplementary Table 6: Details of evidence grading for meta-analyses associating both continuous and categorical contrasts of obesity and risk of cancer incidence or mortality — only cohort studies included.

Obesity indices	Exposure contrast	Outcome	N*	Sample size Cases/Cohort±	Largest study#	Random effects Summary RR (95% CI)†	Random P-value¶	95% Prediction interval	10% credibility RR (95% CI)	Egger's P‡	I^2 (%)	Excess significance§ O/E**	Excess significance§ P-value ||
**Associations supported by strong evidence**													
BMI	per 5kg/m^2 increase	Oesophageal AdCa inc	8	1,918 / 6,430,994	1.67 (1.44-1.92)	1.53 (1.41-1.67)	1.4E-22	1.38-1.71	1.36 (1.12-1.65)	0.19	0	6/6.5	NP
BMI	30+ vs. < 25	Gastrc cardia and oesophageal AdCa	9	2,391 / 5,887,984	1.93 (1.56-2.39)	2.19 (1.84-2.58)	9.4E-20	1.46-3.25	1.93 (1.35-2.76)	0.29	34	8/8.4	NP
BMI	25-29.99 vs. < 25	Gastrc cardia and oesophageal AdCa	10	2,832 / 6,265,992	1.35 (1.11-1.64)	1.48 (1.35-1.61)	3.3E-17	1.33-1.64	1.41 (1.18-1.68)	0.50	0	6/6.7	NP
BMI	per 5kg/m^2 increase	Colon CA inc, men	24	42,821 / 5,213,139	1.27 (1.23-1.31)	1.30 (1.25-1.35)	<1E-100	1.19-1.42	1.24 (1.14-1.34)	0.30	24	18/15.4	0.39
WG	high gain vs. stable weight	Colorectal CA inc	19	28,530 / 2,110,272	1.26 (1.15-1.38)	1.18 (1.12-1.25)	3.7E-9	1.11-1.26	1.11 (1.03-1.20)	0.83	0	2/15.8	NP
BMI	per 5kg/m^2 increase	Rectal CA inc, men	17	23,167 / 4,293,489	1.08 (1.05-1.12)	1.09 (1.06-1.13)	1.1E-7	1.04-1.15	1.08 (1.02-1.13)	0.66	6	3/3.2	NP

BMI	30+ vs. < 25	Rectal CA inc, men	11	12,109 / 2,178,376	1.27 (1.16-1.38)	1.28 (1.19-1.39)	4.8E-10	1.17-1.40	1.27 (1.06-1.51)	0.44	0	5.3	NP
BMI	30+ vs. < 25	Gallbladder CA inc	13	5,516 / 12,517,024	1.88 (1.60-2.21)	1.69 (1.48-1.92)	3.6E-15	1.32-2.15	1.43 (1.15-1.79)	0.11	14	7/10.4	NP
BMI	per 5kg/m^2 increase	Biliary tract system CA inc	10	6,961 / 6,008,270	1.88 (1.60-2.21)	1.56 (1.34-1.81)	1.3E-8	1.13-2.15	1.38 (1.13-1.68)	0.24	25	4/9.00	NP
BMI	per 5kg/m^2 increase	Pancreatic CA inc	22	8,987 / 4,978,230	1.09 (1.03-1.16)	1.10 (1.06-1.14)	2.2E-7	1.01-1.21	1.06 (1.02-1.10)	0.34	25	4/3.4	0.77
WG	per 5kg increase	Breast CA inc, PoMP HRT -	7	10,283 / 342,249	1.14 (1.11-1.18)	1.11 (1.09-1.13)	5.7E-23	1.07-1.15	1.08 (1.03-1.14)	0.66	17	5/4.5	1.00
WG	highest vs. lowest category	Breast CA inc, PoMP HRT -	7	10,283 / 342,249	1.98 (1.55-2.53)	1.75 (1.53-2.00)	7.8E-17	1.47-2.08	1.57 (1.19-2.06)	0.43	0	5/6.9	NP
WHR	per 0.1 unit increase	Endometrial CA inc	5	2,447 / 394,340	1.33 (1.18-1.51)	1.21 (1.13-1.29)	1.0E-8	1.09-1.34	1.16 (1.04-1.32)	0.54	0	3/4.4	NP
BMI	per 5kg/m^2 increase	Endometrial CA inc: PrMP	6	5,981 / 2,558,935	1.53 (1.48-1.58)	1.49 (1.39-1.61)	3.1E-27	1.27-1.76	1.36 (1.11-1.67)	0.56	20	5/4.2	0.67
BMI	30+ vs. < 25	Ovarian CA inc	13	6,947 / 20,560,388 pyrs	1.27 (1.19-1.36)	1.27 (1.17-1.38)	2.6E-8	1.09-1.47	1.16 (1.02-1.30)	0.88	12	3/5.3	NP
BMI	30+ vs. < 25	Kidney CA inc	31	29,979 / 15,477,631	1.85 (1.66-2.06)	1.79 (1.64-1.95)	3.4E-38	1.31-2.43	1.52 (1.32-1.75)	0.53	44	22/30.2	NP
BMI	per 5kg/m^2 increase	Kidney CA inc, men	9	8,983 / 3,945,950	1.19 (1.13-1.24)	1.24 (1.17-1.32)	5.9E-12	1.10-1.41	1.19 (1.06-1.33)	0.16	22	5/4.0	0.52
BMI	per 5kg/m^2 increase	Kidney CA inc, women	10	8,795 / 4,252,718	1.35 (1.28-1.42)	1.33 (1.25-1.42)	7.2E-18	1.13-1.57	1.28 (1.12-1.46)	0.39	45	9/6.9	0.19
BMI	25-29.99 vs. < 25	Multiple myeloma inc	20	15,990 / 8,510,803	1.14 (1.06-1.22)	1.12 (1.07-1.18)	3.4E-7	1.07-1.18	1.10 (1.02-1.18)	0.72	0	2/5.3	NP
BMI	per 5kg/m^2 increase	Multiple myeloma inc	13	14,634 / 7,429,478	1.11 (1.07-1.15)	1.12 (1.09-1.15)	2.8E-15	1.08-1.15	1.09 (1.03-1.17)	0.65	0	4/3.5	0.76
BMI	per 5kg/m^2 increase	Multiple myeloma inc, women	6	7,183 / 3,607,950	1.11 (1.07-1.15)	1.11 (1.08-1.15)	2.8E-10	1.06-1.17	1.12 (1.02-1.23)	0.82	0	2/1.7	0.67
BMI	30+ vs. < 25	Multiple myeloma mort	7	3,239 / 3,604,489	1.46 (1.16-1.84)	1.54 (1.35-1.76)	2.1E-10	1.29-1.83	1.50 (1.12-2.02)	0.32	0	4/4.7	NP

Associations supported by highly suggestive evidence

BMI	per 5kg/m^2 increase	Oesophageal SqCa inc	5	3,545 / 6,369,699	0.77 (0.70-0.85)	0.63 (0.53-0.75)	1.71E-7	0.34-1.15	0.68 (0.52-0.91)	0.26	80	5/3.5	0.33
BMI	per 5kg/m^2 increase	Oesophageal SqCa inc, women	2	1,758 / 3,224,247	0.61 (0.54-0.70)	0.57 (0.48-0.67)	1.2E-10	NA	0.57 (0.31-1.05)	NA	58	2/2.0	1.00
BMI	per 5kg/m^2 increase	Colon CA inc	44	82,766 / 9,434,650	1.03 (1.01-1.05)	1.22 (1.17-1.28)	3.9E-16	0.95-1.57	1.08 (1.05-1.11)	<0.01	84	27/4.4	<0.01
BMI	BMI 30+ vs. < 25	Colon CA inc, men	14	17,387 / 2,192,572	1.49 (1.39-1.60)	1.53 (1.44-1.63)	<1E-100	1.43-1.64	1.60 (1.28-1.98)	0.02	0	9/11.1	NP
BMI	BMI 30+ vs. < 25	Colon CA inc	28	37,136 / 3,946,598	1.07 (1.02-1.12)	1.44 (1.28-1.62)	1.8E-9	0.91-2.28	1.15 (1.06-1.24)	0.01	76	15/4.4	<0.01
WC	per 10cm increase	Colon CA inc	8	3,371 / 869,185	1.14 (1.05-1.25)	1.25 (1.15-1.35)	3.0E-8	1.01-1.54	1.18 (1.07-1.31)	0.063	50	7/2.4	<0.01
BMI	Obese vs. normal	Colorectal CA inc	72	166,030 / 15,623,976	1.06 (1.02-1.10)	1.33 (1.25-1.42)	1.7E-19	0.94-1.50	1.11 (1.06-1.16)	0.001	69	31/14.2	<0.01
WC	highest vs. lowest category	Colorectal CA inc	19	12,566 / 2,041,675	1.32 (1.11-1.56)	1.45 (1.33-1.59)	9.0E-16	1.24-1.70	1.35 (1.19-1.55)	0.015	9	13/14.5	NP
BMI	30+ vs. < 25	Liver CA inc	19	22,378 / 8,574,903	1.33 (1.19-1.48)	1.83 (1.59-2.11)	6.5E-17	1.10-3.04	1.51 (1.26-1.81)	0.03	68	15/1.1	0.07
BMI	per 5kg/m^2 increase	Liver CA inc	19	17,281 / 4,977,849	1.12 (1.05-1.19)	1.37 (1.23-1.53)	2.4E-8	0.90-2.07	1.12 (1.04-1.20)	0.05	81	10/4.7	0.01
BMI	per 5kg/m^2 increase	Lung CA inc	15	12,591 / 2,833,306	0.86 (0.82-0.90)	0.77 (0.72-0.83)	8.0E-13	0.61-0.97	0.84 (0.78-0.92)	0.05	71	10/7.1	0.19
BMI	per 5kg/m^2 increase	Lung CA inc, men	10	7,569 / 1,387,620	0.84 (0.79-0.88)	0.79 (0.73-0.85)	1.7E-10	0.65-0.95	0.83 (0.75-0.92)	0.07	53	7/5.0	0.23
BMI	per 5kg/m^2 increase	Breast CA inc, PoMP	29	27,700 / 2,318,992	1.18 (1.15-1.22)	1.13 (1.09-1.17)	4.1E-10	0.97-1.31	1.06 (1.03-1.10)	0.67	61	12/16.6	NP
BMI	30+ vs. < 25	Endometrial CA inc	6	4,327 / 1,485,506	2.73 (2.48-2.99)	3.10 (2.63-3.65)	<1E-100	1.92-5.00	2.99 (1.49-6.02)	0.24	66	6/6.0	1.00
BMI iya	per 5kg/m^2 increase	Endometrial CA inc	9	4,345 / 631,915	1.23 (1.11-1.35)	1.45 (1.28-1.64)	1.9E-9	0.98-2.15	1.33 (1.14-1.55)	0.41	75	8/5.2	0.09
BMI	per 5kg/m^2 increase	Endometrial CA inc	28	22,320 / 6,445,255	1.65 (1.60-1.71)	1.54 (1.47-1.61)	<1E-100	1.26-1.89	1.41 (1.26-1.57)	0.35	81	26/25.2	1.00
WC	per 10cm increase	Endometrial CA inc	4	1,524 / 315,770	1.28 (1.19-1.37)	1.27 (1.17-1.39)	7.3E-8	0.88-1.85	1.19 (1.03-1.38)	0.59	70	3/2.9	1.00
WG	per 5kg increase	Endometrial CA inc	7	2,806 / 460,901	1.17 (1.12-1.22)	1.16 (1.12-1.20)	3.7E-18	1.06-1.27	1.13 (1.05-1.22)	0.95	47	6/2.8	0.02
Weight	per 5kg increase	Endometrial CA inc	7	1,778 / 342,382	1.11 (1.08-1.15)	1.17 (1.13-1.22)	7.7E-15	1.04-1.31	1.15 (1.06-1.25)	0.29	62	6/1.0	<0.01
BMI	per 5kg/m^2 increase	Endometrial CA inc, PoMP	6	10,075 / 2,558,935	1.51 (1.45-1.58)	1.60 (1.40-1.83)	1.4E-11	1.01-2.53	1.52 (1.16-1.98)	0.88	89	6/5.0	0.60
BMI	per 5kg/m^2 increase	Endometrial CA inc, Type I	3	7,125 / 1,102,927	1.58 (1.53-1.62)	1.75 (1.51-2.03)	1.8E-13	0.30-10.24	1.71 (1.06-2.77)	0.26	82	3/2.9	1.00
BMI	per 5kg/m^2 increase	Endometrial CA inc, Type II	3	1,059 / 1,102,927	1.35 (1.25-1.46)	1.59 (1.29-1.78)	5.7E-7	0.24-9.67	1.47 (1.03-2.08)	0.52	76	3/1.5	0.25
BMI	obese vs. normal weight	Prostate CA mort	10	14,179 / 2,339,669	1.20 (1.06-1.36)	1.24 (1.15-1.33)	1.8E-8	1.13-1.35	1.22 (1.09-1.37)	0.03	0	4/5.7	NP
BMI	25-29.99 vs. < 25	Kidney CA inc	43	30,501 / 18,988,116	1.18 (1.11-1.26)	1.35 (1.27-1.43)	5.0E-22	1.09-1.66	1.25 (1.16-1.35)	0.003	36	19/19.0	NP

BMI	per 5kg/m^2 increase	Kidney CA inc	19	17,778 / 8,198,668	1.19 (1.13-1.24)	1.30 (1.23-1.36)	9.8E-25	1.12-1.50	1.23 (1.12-1.34)	0.11	49	14/7.9	<0.01
BMI	30+ vs. < 25	Thyroid CA inc	24	16,117 / 22,270,022	1.17 (1.11-1.23)	1.29 (1.21-1.37)	5.6E-14	1.10-1.50	1.22 (1.12-1.33)	<0.01	21	9/11.3	NP
BMI	30+ vs. < 25	Leukemia inc	25	51,914 / 21,228,740	1.42 (1.31-1.54)	1.26 (1.16-1.37)	1.6E-8	0.98-1.62	1.17 (1.07-1.27)	0.29	43	7/19.3	NP

Associations supported by suggestive evidence

BMI	per 5kg/m^2 increase	Oesophageal SqCa, men	3	1,787 / 3,145,452	0.77 (0.70-0.85)	0.71 (0.61-0.84)	6.1E-5	0.14-3.69	0.72 (0.52-0.98)	0.03	44	3/1.8	0.28
BMI	per 5kg/m^2 increase	Colon CA inc, women	20	39,945 / 4,221,511	1.03 (1.01-1.05)	1.12 (1.06-1.17)	6.9E-6	0.96-1.30	1.05 (1.02-1.09)	<0.01	59	9/2.1	<0.01
WC	per 10cm increase	Colon CA inc, men	5	1,869 / 469,320	1.15 (1.01-1.31)	1.33 (1.18-1.50)	1.6E-6	0.95-1.86	1.23 (1.04-1.44)	0.51	45	4/1.5	0.03
WC	per 10cm increase	Colon CA inc, women	3	1,502 / 399,865	1.14 (1.05-1.25)	1.16 (1.08-1.23)	1.1E-5	0.76-1.76	1.16 (1.01-1.32)	0.25	0	3/1.0	0.04
WHR	per 0.1 unit increase	Colon CA inc	8	3,132 / 941,955	1.24 (1.10-1.39)	1.29 (1.17-1.43)	1.1E-6	0.97-1.72	1.17 (1.05-1.31)	0.16	53	6/4.2	0.29
WHR	per 0.1 unit increase	Colon CA inc, men	4	1,563 / 461,754	1.24 (1.05-1.46)	1.43 (1.19-1.71)	1.0E-5	0.71-2.87	1.33 (1.05-1.68)	0.32	56	4/2.1	0.13
WHR	per 0.1 unit increase	Colon CA inc, women	4	1,569 / 480,201	1.24 (1.10-1.39)	1.20 (1.08-1.33)	5.0E-5	0.86-1.69	1.12 (0.98-1.28)	0.90	30	2/2.1	NP
BMI	Obese vs. normal**	Colon CA inc; distal colon, men	7	1,685 / 643,569	1.77 (1.33-2.35)	1.70 (1.36-2.11)	2.3E-6	1.11-2.60	1.53 (1.12-2.09)	0.64	17	3/6.5	NP
BMI	Obese vs. normal**	Colon CA inc; distal colon	20	4,359 / 1,320,435	0.96 (0.75-1.23)	1.59 (1.34-1.89)	1.2E-7	0.94-2.69	1.25 (1.07-1.46)	0.49	44	7/1.3	<0.01
BMI	Obese vs. normal**	Colon CA inc; proximal colon, men	7	1,720 / 643.569	1.18 (0.86-1.62)	1.44 (1.18-1.77)	4.4E-4	1.10-1.89	1.30 (1.03-1.64)	0.44	0	1/2.0	NP
BMI	Obese vs. normal**	Colon CA inc; proximal colon	20	4,680 / 1,320,435	1.02 (0.84-1.24)	1.33 (1.12-1.57)	9.6E-4	0.84-2.09	1.14 (1.00-1.29)	0.12	33	3/1.1	0.05
BMI	Obese vs. normal**	Colon CA inc; distal colon, women	12	2,565 / 669,300	0.96 (0.75-1.23)	1.53 (1.19-1.97)	8.3E-4	0.74-3.19	1.17 (0.97-1.40)	0.51	56	4/0.7	<0.01
WG	per 5kg increase	Colon CA inc, men	4	1,718 / 142,085	1.10 (1.03-1.17)	1.09 (1.05-1.14)	4.1E-5	1.00-1.19	1.07 (1.00-1.14)	0.56	0	2/0.8	0.12
WG	per 5kg increase	Colon CA inc	7	2.909 / 298,174	1.10 (1.03-1.17)	1.07 (1.03-1.10)	8.3E-5	1.02-1.11	1.04 (1.00-1.09)	0.88	0	2/1.3	0.50
WG	per 1kg increase / year	Colorectal CA inc	17	27,638 / 1,974,918	1.31 (1.18-1.45)	1.20 (1.10-1.31)	3.9E-5	0.95-1.52	1.11 (1.03-1.21)	0.72	37	4/15.3	NP
BMI	30+ vs. < 25	Rectal CA inc	21	39,384 / 6,768,071	1.04 (0.97-1.11)	1.21 (1.10-1.34)	1.8E-4	0.97-1.52	1.07 (1.01-1.14)	0.08	31	3/2.9	1.00
BMI	per 5kg/m^2 increase	Rectal CA inc	31	43,196 / 7,495,868	1.01 (0.98-1.04)	1.07 (1.03-1.10)	5.9E-5	0.98-1.16	1.03 (1.00-1.05)	0.35	27	4/1.7	0.09
BMI	30+ vs. < 25	Cholangiocarcinoma inc	6	1,478 / 10,201,493	1.36 (0.92-2.02)	1.77 (1.32-2.38)	1.5E-4	1.07-2.93	1.61 (1.15-2.25)	0.09	7	2/3.2	NP
WC	per 10cm increase	Pancreatic CA inc	5	1,365 / 1,154,996	1.08 (0.98-1.18)	1.11 (1.05-1.18)	2.0E-4	1.02-1.22	1.11 (1.02-1.20)	0.11	0	2/0.5	0.09
WHR	per 0.1 unit increase	Pancreatic CA inc	4	1,438 / 1,109,945	1.32 (1.12-1.56)	1.20 (1.09-1.31)	9.6E-5	0.96-1.50	1.14 (1.01-1.28)	0.54	7	2/3.0	NP
BMI	per 5kg/m^2 increase	Lung CA inc, smokers	4	6,185 / 475,940	0.82 (0.77-0.87)	0.77 (0.69-0.86)	7.8E-6	0.48-1.24	0.81 (0.68-0.97)	0.12	78	4/2.9	0.58
BMI	25+ vs. <25	Lung CA inc	20	13,116 / 7,854,110	0.97 (0.93-1.01)	0.79 (0.73-0.85)	9.9E-10	0.59-1.04	0.94 (0.90-0.98)	0.02	80	10/1.4	<0.01
Obesity±±	highest vs. lowest category	Breast CA inc, ER+ PR+, PoMP	4	3,220 / 142,655	1.38 (1.12-1.71)	1.74 (1.34-2.25)	3.1E-5	0.60-5.06	1.55 (1.09-2.21)	0.70	69	3/4.0	NP
BMI	30+ vs. < 25	Breast CA inc, PoMP	8	11,882 / 873,142	1.19 (1.10-1.27)	1.16 (1.08-1.25)	8.1E-5	0.93-1.44	1.09 (1.02-1.16)	0.37	65	4/6.4	NP
BMI	25-29.99 vs. < 25	Breast CA inc, PoMP	8	13,878 / 1,008,448	1.06 (0.99-1.13)	1.12 (1.06-1.18)	5.6E-5	0.96-1.30	1.08 (1.02-1.13)	0.16	57	4/2.1	0.12
BMI	per 5kg/m^2 increase	Breast CA inc, PrMP	20	20,820 / 2,200,787	0.91 (0.86-0.97)	0.92 (0.88-0.97)	4.6E-4	0.81-1.05	0.95 (0.90-0.99)	0.28	38	7/6.3	0.81
BMI	per 5kg/m^2 increase	Ovarian CA inc	24	17,734 / 16,343,135	0.97 (0.93-1.01)	1.08 (1.04-1.12)	8.6E-5	0.96-1.21	1.03 (1.00-1.06)	0.07	48	6/1.8	<0.01
BMI iya	per 5kg/m^2 increase	Ovarian CA inc	6	9,452 / 11,085,425	1.16 (1.04-1.29)	1.12 (1.05-1.19)	3.8E-4	1.03-1.23	1.10 (1.01-1.19)	0.60	0	1/2.5	NP
BMI	per 5kg/m^2 increase	Prostate CA inc, loc	12	19,130 / 1,033,009	0.96 (0.93-0.98)	0.94 (0.91-0.97)	4.6E-4	0.88-1.01	0.95 (0.91-0.99)	0.46	20	3/1.7	0.40
BMI	per 5kg/m^2 increase	Prostate CA mort	6	6,817 / 1,263,483	1.08 (1.04-1.12)	1.15 (1.06-1.25)	8.3E-4	0.92-1.44	1.10 (1.02-1.18)	0.30	59	4/1.6	0.049
BMI	30+ vs. < 25	Bladder CA inc	26	73,491 / 26,538,464	1.13 (1.06-1.20)	1.09 (1.04-1.15)	9.0E-4	0.98-1.21	1.06 (1.00-1.12)	0.71	15	4/13.4	NP
BMI	per 5kg/m^2 increase	Thyroid CA inc	7	6,716 / 5,316,380	1.12 (1.05-1.19)	1.23 (1.10-1.39)	4.3E-4	0.89-1.70	1.13 (1.03-1.24)	0.09	62	4/2.4	0.24
BMI	per 5kg/m^2 increase	Thyroid CA inc, women	3	3,190 / 2,159,238	1.12 (1.05-1.19)	1.14 (1.06-1.22)	4.0E-4	0.67-1.93	1.15 (0.99-1.33)	0.41	3	2/1.1	0.30
BMI	per 5kg/m^2 increase	NHL inc	23	29,064 / 9,056,494	1.04 (0.99-1.09)	1.07 (1.04-1.10)	1.9E-6	1.02-1.12	1.05 (1.02-1.09)	0.26	9	5/2.7	0.18
BMI	per 5kg/m^2 increase	Multiple myeloma inc, men	7	7,451 / 3,821,528	1.13 (1.07-1.19)	1.12 (1.06-1.18)	1.1E-4	1.02-1.22	1.07 (0.98-1.17)	0.76	5	2/2.0	NP
BMI	per 5kg/m^2 increase	Leukemia inc	8	15,117 / 6,758,260	1.07 (1.02-1.12)	1.12 (1.05-1.18)	2.3E-4	0.96-1.30	1.07 (1.01-1.14)	0.22	51	4/2.4	0.25
BMI	30+ vs. < 25	Leukaemia inc, CLL	5	6,547 / 13,038,983	1.30 (1.13-1.49)	1.25 (1.11-1.41)	2.3E-4	0.92-1.70	1.17 (1.02-1.34)	0.78	31	2/4.2	NP

BMI	30+ vs. < 25	Leukaemia inc, AML	6	4,804 / 13,375,364	1.17 (1.00-1.36)	1.52 (1.19-1.95)	8.9E-4	0.69-3.38	1.20 (1.03-1.40)	0.27	79	4/3.4	0.61
BMI	30+ vs. < 25	Leukemia mort	8	6,527 / 4,205,316	1.37 (1.13-1.67)	1.28 (1.11-1.49)	7.5E-4	0.93-1.77	1.18 (1.02-1.37)	0.99	30	4/6.6	NP
**Associations supported by weak evidence**													
BS	surgery vs. no surgery	Overall CA inc	4	1,745 / 31,807	0.75 (0.64-0.88)	0.40 (0.22-0.71)	1.8E-3	0.03-6.14	0.69 (0.49-0.97)	0.03	94	4/3.3	1.00
BMI	per 5kg/m^2 increase	Oesophageal AdCa inc, men	5	996 / 3,196,747	1.67 (1.44-1.92)	1.52 (1.33-1.74)	1.3E-9	1.09-2.11	1.31 (1.04-1.64)	0.37	24	4/3.6	1.00
BMI	per 5kg/m^2 increase	Oesophageal AdCa inc, women	3	922 / 3234247	1.54 (1.26-1.89)	1.51 (1.30-1.75)	2.8E-8	0.59-3.86	1.49 (1.05-2.12)	0.44	0	2/2.7	NP
CeAd##	high vs. low	Oesophageal AdCa inc	2	341 / 565,067	1.81 (1.24-2.64)	1.78 (1.26-2.52)	1.0E-3	NA	1.73 (0.93-1.21)	0.66	0	1/1.9	NP
BMI	30+ vs. < 25	Colon CA inc, women	14	19,749 / 1,754,026	1.07 (1.02-1.12)	1.22 (1.07-1.40)	3.4E-3	0.85-1.75	1.09 (1.01-1.19)	0.04	51	6/2.2	<0.01
BS	surgery vs. no surgery	Colorectal CA inc	5	1,013 / 182,298	0.79 (0.57-1.10)	0.77 (0.62-0.96)	0.02	0.54-1.10	0.78 (0.61-0.99)	0.06	0	0/2.00	NP
BMI	25-29.99 vs. < 25	Liver CA inc	14	16,382 / 3,976,906	1.07 (1.00-1.15)	1.18 (1.06-1.31)	1.7E-3	0.88-1.58	1.07 (1.01-1.14)	0.08	61	4/2.6	0.31
BMI	25-29.99 vs. < 25	Cholangiocarcinoma inc	7	1,753 / 2,332,590	1.40 (1.11-1.72)	1.29 (1.11-1.51)	1.3E-3	1.05-1.58	1.22 (1.00-1.48)	0.22	0	1/5.4	NP
BMI	per 5kg/m^2 increase	Lung CA inc, women	5	5,022 / 1,445,686	0.86 (0.82-0.90)	0.76 (0.61-0.94	0.01	0.36-1.63	0.88 (0.75-1.03)	0.46	86	3/3.0	1.00
BMI	25-29.99 vs. < 25	Melanoma inc, men	6	1,736 / 2,208,423	1.27 (1.12-1.45)	1.41 (1.09-1.83)	9.6E-3	0.75-2.64	1.26 (0.99-1.61)	0.16	35	2/2.6	NP
BMI	30+ vs. < 25	Melanoma inc, men	7	3,839 / 1,399,090	1.29 (1.14-1.48)	1.31 (1.10-1.57)	3.1E-3	0.89-1.93	1.24 (0.98-1.57)	0.89	26	3/4.5	NP
BMI	25+ vs. <25, ASE	Melanoma inc, women	5	1,215 / 803,360	0.99 (0.65-1.50)	0.80 (0.65-0.99)	0.04	0.56-1.13	0.84 (0.67-1.07)	0.12	0	1/0.3	0.23
BMI	per 5kg/m^2 increase	Melanoma inc, men	6	6,647 / 2,808,095	1.16 (1.08-1.26)	1.17 (1.05-1.30)	4.9E-3	0.89-1.52	1.11 (0.96-1.27)	0.89	46	3/2.5	0.69
BMI	per 5kg/m^2 increase	Melanoma inc, women	5	7,148 / 2,886,890	0.97 (0.91-1.00)	0.96 (0.93-1.00)	0.03	0.90-1.02	0.97 (0.93-1.01)	0.28	0	1/0.5	0.40
Obesity±±	highest vs. lowest cat.**	Breast CA inc, ER- PR+, PoMP	2	2,127 / 88,952	1.46 (0.58-3.68)	2.03 (1.04-3.95)	0.04	NA	1.73 (0.77-3.85)	NA	61	1/2.0	NP
Obesity±±	highest vs. lowest cat.**	Breast CA inc, ER+ PR-, PoMP	2	2,127 / 88,952	0.76 (0.49-1.17)	0.64 (0.42-0.95)	0.04	NA	0.72 (0.48-1.07)	NA	27	1/0.1	0.10
BMI	25-29.99 vs. < 25	Cervical CA inc	2	1453 / 5318484	1.10 (1.03-1.17)	1.10 (1.03-1.17)	4.3E-3	NA	1.08 (0.94-1.25)	NA	36	1/0.6	0.49
BS	surgery vs. no surgery	Endometrial CA inc	3	12,288 / 958,988	0.48 (0.43-0.55)	0.39 (0.19-0.79)	8.6E-3	0.0-1314.2	0.55 (0.25-1.20)	0.74	80	2/2.5	NP
BMI	25-29.99 vs. < 25	Endometrial CA inc	7	4,548 / 1,489,424	1.79 (1.65-1.95)	1.60 (1.10-2.33)	0.01	0.42-6.09	1.67 (1.13-2.45)	0.71	96	7/6.9	1.00
HC	per 10 cm increase	Endometrial CA inc	2	831 / 255,650	1.32 (1.20-1.45)	1.29 (1.19-1.41)	2.9E-09	NA	1.23 (0.98-1.54)	NA	0	1/1.7	NP
BMI	per 5kg/m^2 increase	Endometrial CA inc: PoMP HRT-	3	699 / 360,326	1.61 (1.41-1.85)	1.90 (1.56-2.30)	9.2E-11	0.19-18.56	1.80 (1.06-3.07)	<0.01	77	3/2.9	1.00
BMI	per 5kg/m^2 increase	Endometrial CA inc: PoMP HRT+	6	791 / 679,271	1.25 (1.05-1.47)	1.18 (1.07-1.31)	1.7E-3	1.02-1.37	1.16 (1.02-1.31)	0.77	0	1/1.7	NP
WG	per 5kg increase	Endometrial CA inc: PoMP, HRT-	2	285 / 33,340	1.36 (1.25-1.46)	1.38 (1.28-1.49)	3.6E-17	NA	1.40 (0.97-2.02)	NA	0	2/1.0	0.50
WG	per 5kg increase	Endometrial CA inc: PoMP, HRT+	2	334 / 35,333	1.09 (1.00-1.18)	1.09 (1.02-1.17)	0.01	NA	1.09 (0.99-1.20)	NA	0	1/0.2	0.18
BMI	per 5kg/m^2 increase	Endometrial CA mort	3	962 / 1,781,648	1.42 (1.33-1.63)	1.46 (1.29-1.65)	1.7E-9	0.48-4.48	1.35 (0.90-2.05)	0.64	29	2/2.0	1.00
Weight	per 5kg increase	Ovarian CA inc	4	1,281 / 405,655	1.02 (1.00-1.05)	1.03 (1.01-1.05)	1.3E-3	0.98-1.08	1.03 (1.00-1.05)	0.42	7	1/0.2	0.20
WG	per 5kg increase	Ovarian CA inc: PostMP, HRT-	2	217 / 23,984	1.16 (1.03-1.31)	1.13 (1.03-1.24)	8.5E-3	NA	1.11 (0.99-1.25)	NA	0	0.27	0.25
BMI	30+ vs. < 25	Ovarian CA inc, PoMP	5	1,116 / 1,858,520 pyrs	1.02 (0.82-1.26)	1.23 (1.03-1.47)	0.03	0.72-2.09	1.12 (0.97-1.29)	0.52	46	0.3	0.14
BMI	30+ vs. < 25	Ovarian CA inc, PrMP	3	284 / 1,758,718 pyrs	1.56 (1.14-2.16)	1.57 (1.20-2.06)	9.7E-4	0.27-9.02	1.54 (0.95-2.51)	0.61	0	1.7	NP
BMI	per 5kg/m^2 increase	Prostate CA inc, adv	13	7,067 / 1,080,790	1.00 (0.94-1.08)	1.09 (1.02-1.16)	0.01	0.92-1.29	1.04 (0.99-1.09)	0.02	40	3/0.7	0.02
WG	per 5kg increase	Prostate CA inc, high screening rate	2	4,894 / 67,335	0.96 (0.92-1.00)	0.95 (0.92-0.99)	7.0E-3	NA	0.95 (0.90-1.01)	NA	0	1/0.4	0.25
WG	highest vs. lowest category	Kidney CA inc	3	1,780 / 781,293	1.20 (0.90-1.70)	1.42 (1.11-1.81)	4.7E-3	0.23-8.71	1.28 (0.97-1.69)	0.89	10	1/2.0	NP
BMI	25-29.99 vs. < 25	Bladder CA inc	26	28,745 / 12,562,510	1.05 (1.00-1.10)	1.07 (1.01-1.14)	0.03	0.90-1.27	1.04 (0.99-1.08)	0.29	38	5/3.7	0.41
BMI	30+ vs. < 25	Meningeoma inc	4	799 / 1,995,708	1.40 (1.08-1.87)	1.55 (1.28-1.86)	4.4E-6	1.03-2.33	1.49 (1.09-2.04)	0.54	0	2/2.6	NP
BMI	per 5kg/m^2 increase	Thyroid CA inc, men	4	3,526 / 3,157,142	1.09 (0.98-1.22)	1.32 (1.04-1.69)	0.02	0.46-3.84	1.12 (1.00-1.25)	0.23	77	2/1.0	0.27
BMI	30+ vs. < 25	Hodgkin's lymphoma inc	4	2,716 / 4,828,366	1.66 (1.28-2.14)	1.41 (1.14-1.75)	1.9E-3	0.73-2.72	1.24 (0.96-1.59)	0.74	22	1/3.5	NP
BMI	30+ vs. < 25	NHL inc	15	24,421 / 7,611,421	1.09 (1.00-1.19)	1.19 (1.04-1.37)	0.01	0.78-1.80	1.09 (0.99-1.19)	0.91	66	5/4.5	0.78
BMI	per 5kg/m^2 increase	NHL mort	7	5,920 / 3,511,893	1.18 (1.09-1.29)	1.14 (1.04-1.25)	5.3E-3	0.90-1.45	1.10 (1.01-1.20)	0.57	53	3/3.4	NP

BMI	25-29.99 vs. < 25	Diff large B-cell lymphoma inc	10	2,445 / 2,348,783	1.29 (1.04-1.61)	1.13 (1.02-1.25)	0.02	1.00-1.27	1.10 (0.98-1.23)	0.37	0	1/5.7	NP
BMI	30+ vs. < 25	Diff large B-cell lymphoma inc	12	3,093 / 2,981,742	1.64 (1.25-2.16)	1.25 (1.08-1.44)	2.6E-3	0.89-1.74	1.14 (1.00-1.32)	0.05	27	3/11.6	NP
BMI	30+ vs. < 25	Multiple myeloma inc	19	15,887 / 7,729,520	1.31 (1.16-1.48)	1.21 (1.08-1.35)	1.0E-3	0.90-1.62	1.14 (1.01-1.28)	0.56	34	5/11.2	NP
BMI	25-29.99 vs. < 25	Multiple myeloma mort	8	3,337 / 3,714,187	1.18 (1.01-1.39)	1.15 (1.04-1.26)	6.0E-3	1.02-1.30	1.12 (0.99-1.27)	0.46	0	1/2.8	NP
BMI	per 5kg/m^2 increase	Leukemia inc, men	5	7,827 / 3,497,392	1.09 (1.02-1.17)	1.08 (1.02-1.13)	8.4E-3	0.99-1.17	1.06 (0.98-1.14)	0.47	0	1/1.5	NP
BMI	per 5kg/m^2 increase	Leukemia inc, women	3	7,290 / 3,260,868	1.07 (1.02-1.12)	1.17 (1.04-1.33)	9.8E-3	0.28-4.93	1.10 (1.00-1.21)	0.20	79	3/1.1	0.05
BMI	30+ vs. < 25	Leukemia inc, ALL	5	588 / 13,337,737	1.33 (0.82-2.15)	1.65 (1.16-2.35)	5.1E-3	0.85-3.23	1.40 (0.96-2.04)	0.67	7	1/1.9	NP
BMI	30+ vs. < 25	Leukemia inc, CML	5	2,530 / 13,337,737	1.15 (0.92-1.45)	1.26 (1.09-1.46)	1.7E-3	1.00-1.60	1.20 (1.03-1.41)	0.47	0	1/1.9	NP

Abbreviations: AdCa, adenocarcinoma; adv, advanced; ALL, acute lymphatic leukemia; AML, acutel myeloic leukemia; ASE, adjusted for sunlight exposure; biliary tract system cancer, includes cancers of gallbladder, extrahepatic bile duct and ampulla of Vateri; BMI, Body Mass Index; BMI iya, Body Mass Index in young adulthood; BS, bariatric surgery; CA, cancer; CI, confidence interval; CLL, chronic lymphatic leukemia; CML, chronic myeloic leukemia; ER = estrogen receptor status; HC, hip circumference; HRT, hormone replacement therapy; inc, incidence; loc, localised; mort, mortality; NA: Not available, due to <3 included studies; NHL, Non-Hodgkin lymphoma; NP: Not Pertinent, because the estimated is larger than the observed, and there is no evidence of excess statistical significance based on the assumption made for the plausible effect size; PoMP, postmenopausal; PR = progesterone receptor status; PrMP, premenopausal; pyrs, preson-years; RR, relative risk; SqCa, squamous cell carcinoma; WC, waist circumference; WG, weight gain; WHR, waist-to-hip ratio;

* Number of studies
± Sum of all study cohorts in the meta-analysis. In case the same study appears more than once (e.g. for both men and women, and proportions of men and women in the study cohort was not given), the sum given here may too large.
# Relative risk and 95% confidence interval of largest study (smallest SE) in each meta-analysis.
† Random effects refer to summary risk ratio (95% CI) using the random-effects model.
¶ P value of summary random effects estimate.
‡ P-value from the Egger's regression asymmetry test.
§ Expected number of statistically significant studies using the point estimate of the largest study (smallest standard error) as the plausible effect size.
** Observed/Expected number of statistically significant studies
|| P value of the excess statistical significance test.
** cut-offs in categories varied between included studies
±± BMI, weight gain or weight change, varies between included studies
Central adiposity: dependent categorical comparison of visceral adipose tissue area. if that was unavailable, WHR (or waist thigh ratio) or last WC was used.

All statistical tests were two-sided.

Supplementary table 7: Details of evidence grading for meta-analyses associating both continuous and categorical contrasts of obesity and risk of cancer incidence or mortality — all study types studies included***.

Obesity indices	Exposure contrast	Outcome	N*	Sample size Cases/Cohort±	Largest study#	Random effects Summary RR (95% CI)†	Random P-value¶	95% Prediction interval	10% credibility RR (95% CI)	Egger's P‡	I^2 (%)	Excess significance§ O/E**	Excess significance§ P-value ¦¦
**Associations supported by strong evidence**													
BMI	per 5kg/m^2 increase	Oesophageal AdCa inc	8	1,918 / 6,430,994	1.67 (1.44-1.92)	1.53 (1.41-1.67)	1.4E-22	1.38-1.71	1.36 (1.12-1.65)	0.19	0	6/6.5	NP
BMI	per 5kg/m^2 increase	Colon CA inc, men	24	42,821 / 5,213,139	1.27 (1.23-1.31)	1.30 (1.25-1.35)	<1E-100	1.19-1.42	1.24 (1.14-1.34)	0.30	24	18/15.4	0.39
WG	high gain vs. stable weight	Colorectal CA inc	22	35,187 / 2,124,596	1.26 (1.15-1.38)	1.22 (1.14-1.30)	2.5E-8	1.03-1.43	1.12 (1.04-1.21)	0.70	22	4/18.7	NP
BMI	per 5kg/m^2 increase	Rectal CA inc, men	18	23,480 / 4,296,566	1.08 (1.05-1.12)	1.09 (1.06-1.12)	2.2E-8	1.04-1.13	1.07 (1.02-1.12)	0.77	3	3/3.31	NP
BMI	30+ vs. < 25	Rectal CA inc, men	11	12,109 / 2,178,376	1.27 (1.16-1.38)	1.28 (1.19-1.39)	4.8E-10	1.17-1.40	1.27 (1.06-1.51)	0.44	0	5.3	NP
BMI	30+ vs <25	Gallbladder CA inc	17	6,073 / 12,520,206	1.88 (1.60-2.21)	1.66 (1.47-1.88)	9.8E-16	1.32-2.09	1.39 (1.14-1.70)	0.05	12	8/13.7	NP
BMI	per 5kg/m^2 increase	Pancreatic CA inc	23	9,439 / 4,981,369	1.09 (1.03-1.16)	1.10 (1.06-1.14)	2.5E-8	1.01-1.19	1.06 (1.03-1.10)	0.33	21	4/3.59	0.77
BMI	25-29.99 vs. < 25	Melanoma inc, men	18	4,793 / 2,215,979	1.27 (1.12-1.45)	1.31 (1.18-1.45)	2.3E-7	1.17-1.46	1.30 (1.10-1.54)	0.12	0	3/6.67	NP
WG	per 5 kg increase	Breast CA inc, PoMP HRT -	7	10,283 / 342,249	1.14 (1.11-1.18)	1.11 (1.09-1.13)	5.7E-23	1.07-1.15	1.08 (1.03-1.14)	0.66	17	5/4.47	1.00
WG	highest vs. lowest cat.	Breast CA inc, PoMP HRT -	7	10,283 / 342,249	1.98 (1.55-2.53)	1.75 (1.53-2.00)	7.8E-17	1.47-2.08	1.57 (1.19-2.06)	0.43	0	5/6.94	NP
WHR	per 0.1 unit increase	Endometrial CA inc	5	2,447 / 394,340	1.33 (1.18-1.51)	1.21 (1.13-1.29)	1.0E-8	1.09-1.34	1.16 (1.04-1.32)	0.54	0	3/4.44	NP
BMI	per 5kg/m^2 increase	Endometrial CA inc: PrMP	6	5,981 / 2,558,935	1.53 (1.48-1.58)	1.49 (1.39-1.61)	3.1E-27	1.27-1.76	1.36 (1.11-1.67)	0.56	20	5/4.2	0.67
BMI	30+ vs. < 25	Ovarian CA inc	13	6,947 / 20,560,388 pyrs	1.27 (1.19-1.36)	1.27 (1.17-1.38)	2.6E-8	1.09-1.47	1.16 (1.02-1.30)	0.88	12	3/5.3	NP
BMI	30+ vs <25	Kidney CA inc	31	29,979 / 15,477,631	1.85 (1.66-2.06)	1.79 (1.64-1.95)	3.4E-38	1.31-2.43	1.52 (1.32-1.75)	0.53	44	22/30.2	NP
BMI	per 5kg/m^2 increase	Kidney CA inc, men	11	9,277 / 3,946,646	1.19 (1.13-1.24)	1.24 (1.15-1.34	9.0E-9	1.05-1.47	1.16 (1.05-1.29)	0.20	36	6/4.26	0.36
BMI	per 5kg/m^2 increase	Kidney CA inc, women	12	9,243 / 4,253,611	1.35 (1.28-1.42)	1.34 (1.25-1.43)	5.8E-19	1.14-1.58	1.26 (1.11-1.42)	0.46	44	10/7.73	0.23
BMI	30+ vs <25	Meningeoma inc	6	2,069 / 1,998,356	1.29 (1.01-1.65)	1.45 (1.26-1.67)	2.7E-7	1.19-1.78	1.42 (1.13-1.78)	0.20	0	3/3.09	NP
BMI	per 5kg/m^2 increase	Multiple myeloma inc	13	14,634 / 7,429,478	1.11 (1.07-1.15)	1.12 (1.09-1.15)	2.8E-15	1.08-1.15	1.09 (1.03-1.17)	0.65	0	4/3.49	0.76
BMI	per 5kg/m^2 increase	Multiple myeloma inc, women	6	7,183 / 3,607,950	1.11 (1.07-1.15)	1.11 (1.08-1.15)	2.8E-10	1.06-1.17	1.12 (1.02-1.23)	0.82	0	2/1.67	0.67
BMI	25-29.99 vs. < 25	Multiple myeloma inc	20	15,990 / 8,510,803	1.14 (1.06-1.22)	1.12 (1.07-1.18)	3.4E-7	1.07-1.18	1.10 (1.02-1.18)	0.72	0	2/5.27	NP
BMI	30+ vs <25	Multiple myeloma mort	7	3,239 / 3,604,489	1.46 (1.16-1.84)	1.54 (1.35-1.76)	2.1E-10	1.29-1.83	1.50 (1.12-2.02)	0.32	0	4/4.69	NP

Associations supported by highly suggestive evidence

BMI	per 5kg/m^2 increase	Oesophageal SqCa inc	5	3,545 / 6,369,699	0.77 (0.70-0.85)	0.63 (0.53-0.75)	1.7E-7	0.34-1.15	0.68 (0.52-0.91)	0.26	80	5/3.46	0.33
BMI	per 5kg/m^2 increase	Oesophageal SqCa inc, women	2	1,758 / 3,224,247	0.61 (0.54-0.70)	0.57 (0.48-0.67)	1.2E-10	N/A	0.57 (0.31-1.05)	N/A	58	2/1.99	1.00
BMI	30+ vs. < 25	Gastrc cardia and oesophageal AdCa	13	3.029 / 5,890,512	1.93 (1.56-2.39)	2.34 (1.95-2.81)	7.3E-20	1.36-4.02	2.01 (1.46-2.77)	0.07	54	11/11.4	NP
BMI	25-29.99 vs. < 25	Gastrc cardia and oesophageal AdCa	23	5,385 / 6,276,175	1.35 (1.11-1.64)	1.71 (1.50-1.96)	2.9E-15	1.03-2.85	1.37 (1.20-1.55)	<0.01	64	14/12.4	0.51
BMI	per 5kg/m^2 increase	Colon CA inc	45	82,839 / 9,434,867	1.03 (1.01-1.05)	1.23 (1.17-1.29)	1.1E-16	0.95-1.58	1.08 (1.05-1.11)	<0.01	84	28/4.44	<0.01
WC	per 10cm increase	Colon CA inc	8	3,371 / 869,185	1.14 (1.05-1.25)	1.25 (1.15-1.35)	3.0E-8	1.01-1.54	1.18 (1.07-1.31)	0.06	50	7/2.41	<0.01
BMI	BMI 30+ vs. < 25	Colon CA inc, men	14	17,387 / 2,192,572	1.49 (1.39-1.60)	1.53 (1.44-1.63)	<1E-100	1.43-1.64	1.60 (1.28-1.98)	0.02	0	9/11.1	NP
BMI	BMI 30+ vs. < 25	Colon CA inc	28	37,136 / 3,946,598	1.07 (1.02-1.12)	1.44 (1.28-1.62)	1.8E-9	0.91-2.28	1.15 (1.06-1.24)	0.01	76	15/4.4	<0.01
BMI	Obese vs. normal weight	Colorectal CA inc	72	166,030 / 15,623,976	1.06 (1.02-1.10)	1.33 (1.25-1.42)	1.7E-19	0.94-1.50	1.11 (1.06-1.16)	<0.01	69	31/14.2	<0.01
WC	highest vs. lowest category	Colorectal CA inc	19	12,566 / 2,041,675	1.32 (1.11-1.56)	1.45 (1.33-1.59)	9.0EE-16	1.24-1.70	1.35 (1.19-1.55)	0.02	9	13/14.5	NP
WG	per 1kg increase / year	Colorectal CA inc	20	34,295 / 1,989,242	1.31 (1.18-1.45)	1.24 (1.14-1.36)	7.8E-07	0.95-1.63	1.12 (1.04-1.21)	0.56	47	6/18.3	NP
BMI	30+ vs. < 25	Liver CA inc	19	22,378 / 8,574,903	1.33 (1.19-1.48)	1.83 (1.59-2.11)	6.5E-17	1.10-3.04	1.51 (1.26-1.81)	0.03	68	15/10.9	0.07
BMI	per 5kg/m^2 increase	Liver CA inc	21	17,642 / 5,003,033	1.12 (1.05-1.19)	1.39 (1.25-1.55)	1.8E-9	0.92-2.10	1.12 (1.05-1.21)	0.02	80	12/4.91	<0.01
BMI	per 5kg/m^2 increase	Lung CA inc	17	13,323 / 2,836,216	0.86 (0.82-0.90)	0.77 (0.72-0.83)	9.9E-13	0.60-0.98	0.86 (0.79-0.93)	0.07	73	11/7.62	0.14
BMI	per 5kg/m^2 increase	Lung CA inc, men	11	7,935 / 1,389,075	0.84 (0.79-0.88)	0.76 (0.70-0.83)	7.5E-11	0.61-0.96	0.83 (0.75-0.91)	0.03	62	8/5.36	0.14
BMI	per 5kg/m^2 increase	Breast CA inc, PoMP	31	28,599 / 2,321,914	1.18 (1.15-1.22)	1.12 (1.08-1.16)	3.4E-09	0.96-1.31	1.06 (1.03-1.09)	0.44	62	12/17.3	NP
Obe±±	highest vs. lowest cat.**	Breast CA inc, PoMP, ER+PR+	10	12,460 / 163,259	1.38 (1.12-1.71)	1.82 (1.55-2.14)	2.8E-13	1.12-2.96	1.54 (1.23-1.94)	0.43	61	7/9.85	NP
BMI	25-29.99 vs. < 25	Endometrial CA inc	18	8,606 / 1,502,322	1.79 (1.65-1.95)	1.61 (1.38-1.87)	1.2E-9	0.84-3.08	1.25 (1.11-1.42)	0.59	92	16/16.5	NP
BMI	30+ vs. < 25	Endometrial CA inc	14	7,176 / 1,494,091	2.73 (2.48-2.99)	2.52 (2.12-3.00)	8.5E-26	1.30-4.88	1.64 (1.27-2.11)	0.44	90	14/13.9	1.00
BMI iya	per 5kg/m^2 increase	Endometrial CA inc	9	4,345 / 631,915	1.23 (1.11-1.35)	1.45 (1.28-1.64)	1.9E-09	0.98-2.15	1.33 (1.14-1.55)	0.41	75	8/5.20	0.09
BMI	per 5kg/m^2 increase	Endometrial CA inc	28	22,320 / 6,445,255	1.65 (1.60-1.71)	1.54 (1.47-1.61)	<1E-100	1.26-1.89	1.41 (1.26-1.57)	0.35	81	26/25.2	1.00
WC	per 10cm increase	Endometrial CA inc	4	1,524 / 315,770	1.28 (1.19-1.37)	1.27 (1.17-1.39)	7.3E-08	0.88-1.85	1.19 (1.03-1.38)	0.59	70	3/2.92	1.00
WG	per 5kg increase	Endometrial CA inc	7	2,806 / 460,901	1.17 (1.12-1.22)	1.16 (1.12-1.20)	3.7E-18	1.06-1.27	1.13 (1.05-1.22)	0.95	47	6/2.75	0.02
Weight	per 5kg increase	Endometrial CA inc	8	1,841 / 343,719	1.11 (1.08-1.15)	1.18 (1.14-1.23)	1.9E-15	1.05-1.34	1.16 (1.07-1.26)	0.14	66	7/1.11	<0.01
BMI	per 5kg/m^2 increase	Endometrial CA inc, PoMP	6	10,075 / 2,558,935	1.51 (1.45-1.58)	1.60 (1.40-1.83)	1.4E-11	1.01-2.53	1.52 (1.16-1.98)	0.88	89	6/5.0	0.60
BMI	per 5kg/m^2 increase	Endometrial CA inc, Type I	3	7,125 / 1,102,927	1.58 (1.53-1.62)	1.75 (1.51-2.03)	1.8E-13	0.30-10.2	1.71 (1.06-2.77)	0.26	82	3/2.9	1.00
BMI	per 5kg/m^2 increase	Endometrial CA inc, Type II	3	1,059 / 1,102,927	1.35 (1.25-1.46)	1.59 (1.29-1.78)	5.7E-7	0.24-9.67	1.47 (1.03-2.08)	0.52	76	3/1.5	0.25
BMI	obese vs. normal weight	Prostate cancer mort	10	14,179 / 2,339,669	1.20 (1.06-1.36)	1.24 (1.15-1.33)	1.8E-8	1.13-1.35	1.22 (1.09-1.37)	0.03	0	4/5.73	NP
BMI	25-29.99 vs. < 25	Kidney CA inc	43	30,501 / 18,988,116	1.18 (1.11-1.26)	1.35 (1.27-1.43)	5.0E-22	1.09-1.66	1.25 (1.16-1.35)	<0.01	36	19/19.0	1.00
BMI	per 5kg/m^2 increase	Kidney CA inc	23	18,520 / 8,200,257	1.19 (1.13-1.24)	1.30 (1.23-1.37)	8.1E-24	1.11-1.52	1.20 (1.11-1.30)	0.14	51	16/8.53	<0.01
BMI	30+ vs. < 25	Thyroid CA inc	32	18,152 / 22,274,032	1.17 (1.11-1.23)	1.32 (1.24-1.42)	2.1E-15	1.10-1.59	1.24 (1.14-1.35)	<0.01	25	12/12.9	NP
BMI	30+ vs. < 25	Leukemia inc	25	51,914 / 21,228,740	1.42 (1.31-1.54)	1.26 (1.16-1.37)	1.6E-8	0.98-1.62	1.17 (1.07-1.27)	0.29	43	7/19.3	NP

Associations supported by suggestive evidence

BMI	per 5kg/m^2 increase	Oesophageal SqCa, men	3	1,787 / 3,145,452	0.77 (0.70-0.85)	0.71 (0.61-0.84)	6.1E-5	0.14-3.69	0.72 (0.52-0.98)	0.03	44	3/1.81	0.28
BMI	per 5kg/m^2 increase	Colon CA inc, women	21	40,018 / 4,221,728	1.03 (1.01-1.05)	1.12 (1.07-1.18)	3.3E-6	0.96-1.31	1.05 (1.02-1.09)	<0.01	61	10/2.10	<0.01

WC	per 10cm increase	Colon CA inc, men	5	1,869 / 469,320	1.15 (1.01-1.31)	1.33 (1.18-1.50)	1.6E-6	0.95-1.86	1.23 (1.04-1.44)	0.51	45	4/1.50	0.03
WC	per 10cm increase	Colon CA inc, women	3	1,502 / 399,865	1.14 (1.05-1.25)	1.16 (1.08-1.23)	1.1E-5	0.76-1.76	1.16 (1.01-1.32)	0.25	0	3/1.04	0.04
WHR	per 0.1 unit increase	Colon CA inc	8	3,132 / 941,955	1.24 (1.10-1.39)	1.29 (1.17-1.43)	1.1E-6	0.97-1.72	1.17 (1.05-1.31)	0.16	53	6/4.17	0.29
WHR	per 0.1 unit increase	Colon CA inc, men	4	1,563 / 461,754	1.24 (1.05-1.46)	1.43 (1.19-1.71)	9.98E-5	0.71-2.87	1.33 (1.05-1.68)	0.32	56	4/2.07	0.13
WHR	per 0.1 unit increase	Colon CA inc, women	4	1,569 / 480,201	1.24 (1.10-1.39)	1.20 (1.08-1.33)	5.0E-4	0.86-1.69	1.12 (0.98-1.28)	0.90	30	2/2.10	NP
BMI	Obese vs. normal**	Colon CA inc; distal colon, men	7	1,685 / 643,569	1.77 (1.33-2.35)	1.70 (1.36-2.11)	2.3E-6	1.11-2.60	1.53 (1.12-2.09)	0.64	17	3/6.5	NP
BMI	Obese vs. normal**	Colon CA inc; distal colon	20	4,359 / 1,320,435	0.96 (0.75-1.23)	1.59 (1.34-1.89)	1.2E-7	0.94-2.69	1.25 (1.07-1.46)	0.49	44	7/1.3	<0.01
BMI	Obese vs. normal**	Colon CA inc; proximal colon, men	7	1,720 / 643.569	1.18 (0.86-1.62)	1.44 (1.18-1.77)	4.4E-4	1.10-1.89	1.30 (1.03-1.64)	0.44	0	1/2.0	NP
BMI	Obese vs. normal**	Colon CA inc; proximal colon	20	4,680 / 1,320,435	1.02 (0.84-1.24)	1.33 (1.12-1.57)	9.6E-4	0.84-2.09	1.14 (1.00-1.29)	0.12	33	3/1.1	0.05
BMI	Obese vs. normal**	Colon CA inc; distal colon, women	12	2,565 / 669,300	0.96 (0.75-1.23)	1.53 (1.19-1.97)	8.3E-4	0.74-3.19	1.17 (0.97-1.40)	0.51	56	4/0.7	<0.01
WG	per 5kg increase	Colon CA inc, men	4	1,718 / 142,085	1.10 (1.03-1.17)	1.09 (1.05-1.14)	4.1E-5	1.00-1.19	1.07 (1.00-1.14)	0.56	0	2/0.8	0.12
WG	per 5kg increase	Colon CA inc	7	2.909 / 298,174	1.10 (1.03-1.17)	1.07 (1.03-1.10)	8.3E-5	1.02-1.11	1.04 (1.00-1.09)	0.88	0	2/1.3	0.50
BMI	30+ vs. < 25	Rectal CA inc	21	39,384 / 6,768,071	1.04 (0.97-1.11)	1.21 (1.10-1.34)	1.8E-4	0.97-1.52	1.07 (1.01-1.14)	0.08	31	3/2.87	1.00
BMI	per 5kg/m^2 increase	Rectal CA inc	32	43,509 / 7,498,945	1.01 (0.98-1.04)	1.07 (1.03-1.10)	5.4E-5	0.99-1.15	1.03 (1.00-1.05)	0.38	24	4/1.75	0.09
BMI	25-29.99 vs. < 25	Cholangiocarcinoma inc	8	2,125 / 2,338,722	1.40 (1.11-1.78)	1.30 (1.13-1.49)	2.4E-4	1.09-1.54	1.23 (1.04-1.48)	0.17	0	1/6.31	NP
BMI	30+ vs. < 25	Cholangiocarcinoma inc	12	6,137 / 10,612,420	0.90 (0.70-1.20)	1.52 (1.23-1.89)	1.1E-4	0.83-2.79	1.28 (1.05-1.55)	0.06	51	5/2.88	0.18
BMI	per 5kg/m^2 increase	Biliary tract system CA inc	12	7,147 / 6,083,814	1.88 (1.60-2.21)	1.48 (1.24-1.75)	7.9E-6	0.98-2.24	1.31 (1.09-1.59)	0.09	36	4/10.8	NP
WC	per 10cm increase	Pancreatic CA inc	5	1,365 / 1,154,996	1.08 (0.98-1.18)	1.11 (1.05-1.18)	2E-4	1.02-1.22	1.11 (1.02-1.20)	0.11	0	2/0.54	0.09
WHR	per 0.1 unit increase	Pancreatic CA inc	4	1,438 / 1,109,945	1.32 (1.12-1.56)	1.20 (1.09-1.31)	9.6E-5	0.96-1.50	1.14 (1.01-1.28)	0.54	7	2/3.04	NP
BMI	per 5kg/m^2 increase	Lung CA inc, smokers	5	6,392 / 476,678	0.82 (0.77-0.87)	0.76 (0.67-0.85)	5.5E-6	0.51-1.11	0.80 (0.68-0.95)	0.05	77	5/3.1	0.16
BMI	25+ vs. <25	Lung CA inc, overall	31	26,066 / 7,928,345	0.97 (0.93-1.01)	0.74 (0.68-0.80)	2.6E-14	0.52-1.05	0.93 (0.89-0.97)	<0.01	83	17/2.27	<0.01
BMI	25+ vs. <25, ASE	Melanoma inc, men	7	2,656 / 142,675	1.60 (0.89-2.89)	1.68 (1.29-2.17)	9E-5	1.19-2.35	1.59 (1.17-2.17)	0.81	0	2/6.86	NP
BMI	30+ vs. < 25	Melanoma inc, men	15	5,899 / 1,404,137	1.29 (1.14-1.48)	1.33 (1.14-1.55)	4.0E-4	0.95-1.85	1.23 (1.02-1.49)	0.81	23	5/7.51	NP
BMI	30+ vs. < 25	Breast CA inc, PoMP	8	11,882 / 873,142	1.19 (1.10-1.27)	1.16 (1.08-1.25)	8.1E-5	0.93-1.44	1.09 (1.02-1.16)	0.37	65	4/6.4	NP
BMI	25-29.99 vs. < 25	Breast CA inc, PoMP	8	13,878 / 1,008,448	1.06 (0.99-1.13)	1.12 (1.06-1.18)	5.6E-5	0.96-1.30	1.08 (1.02-1.13)	0.16	57	4/2.1	0.12
BMI	per 5kg/m^2 increase	Breast CA inc, PrMP	20	20,820 / 2,200,787	0.91 (0.86-0.97)	0.92 (0.88-0.96)	4.6E-4	0.81-1.05	0.95 (0.90-0.99)	0.28	38	7/6.3	0.81
WHR	per 0.1 unit increase	Breast CA inc, PrMP	13	8,344 / 149,996	1.23 (1.17-1.29)	1.10 (1.04-1.16)	4.6E-4	0.91-1.32	1.04 (1.01-1.07)	0.99	76	5/6.68	NP
BMI	per 5kg/m^2 increase	Ovarian CA inc	25	17,856 / 16,343,368	0.97 (0.93-1.01)	1.07 (1.03-1.12)	4.6E-4	0.94-1.22	1.03 (1.00-1.07)	0.22	53	7/1.85	<0.01
BMI iya	per 5kg/m^2 increase	Ovarian Ca inc	6	9,452 / 11,085,425	1.16 (1.04-1.29)	1.12 (1.05-1.19)	3.8E-4	1.03-1.23	1.10 (1.01-1.19)	0.60	0	1/2.48	NP
BMI	per 5kg/m^2 increase	Prostate CA inc, localised	12	19,130 / 1,033,009	0.96 (0.93-0.98)	0.94 (0.91-0.97)	4.6E-4	0.88-1.01	0.95 (0.91-0.99)	0.46	20	3/1.71	0.40
BMI	per 5kg/m^2 increase	Prostate cancer mort	6	6,817 / 1,263,483	1.08 (1.04-1.12)	1.15 (1.06-1.25)	8.3E-4	0.92-1.44	1.10 (1.02-1.18)	0.30	59	4/1.62	0.049
BMI	30+ vs. < 25	Bladder CA inc	26	73,491 / 26,538,464	1.13 (1.06-1.20)	1.09 (1.04-1.15)	9.0E-4	0.98-1.21	1.06 (1.00-1.12)	0.71	15	4/13.4	NP
BMI	per 5kg/m^2 increase	Thyroid CA inc	7	6,716 / 5,316,380	1.12 (1.05-1.19)	1.23 (1.10-1.39)	4.3E-4	0.89-1.70	1.13 (1.03-1.24)	0.09	62	4/2.40	0.24
BMI	per 5kg/m^2 increase	Thyroid CA inc, women	3	3,190 / 2,159,238	1.12 (1.05-1.19)	1.14 (1.06-1.22)	4.0E-4	0.67-1.93	1.15 (0.99-1.33)	0.41	3	2/1.10	0.30
BMI	per 5kg/m^2 increase	NHL inc	23	29,064 / 9,056,494	1.04 (0.99-1.09)	1.07 (1.04-1.10)	1.9E-6	1.02-1.12	1.05 (1.02-1.09)	0.26	9	5/2.74	0.18
BMI	30+ vs. < 25	Diff large B-cell lymphoma inc	24	26,831 / 3,129,606	1.45 (1.17-1.80)	1.29 (1.16-1.43)	1.8E-6	0.94-1.77	1.17 (1.06-1.31)	0.13	32	6/21.8	NP

BMI	per 5kg/m^2 increase	Multiple myeloma inc, men	7	7,451 / 3,821,528	1.13 (1.07-1.19)	1.12 (1.06-1.18)	1.1E-4	1.02-1.22	1.07 (0.98-1.17)	0.76	5	2/2.03	NP
BMI	per 5kg/m^2 increase	Leukemia inc	8	15,117 / 6,758,260	1.07 (1.02-1.12)	1.12 (1.05-1.18)	2.3E-4	0.96-1.30	1.07 (1.01-1.14)	0.22	51	4/2.37	0.25
BMI	30+ vs. < 25	Leukaemia inc, CLL	5	6,547 / 13,038,983	1.30 (1.13-1.49)	1.25 (1.11-1.41)	2.3E-4	0.92-1.70	1.17 (1.02-1.34)	0.78	31	2/4.2	NP
BMI	30+ vs. < 25	Leukaemia inc, AML	6	4,804 / 13,375,364	1.17 (1.00-1.36)	1.52 (1.19-1.95)	8.9E-4	0.69-3.38	1.20 (1.03-1.40)	0.27	79	4/3.4	0.61
BMI	30+ vs. < 25	Leukemia mort	8	6,527 / 4,205,316	1.37 (1.13-1.67)	1.28 (1.11-1.49)	7.5E-4	0.93-1.77	1.18 (1.02-1.37)	0.99	30	4/6.61	NP

Associations supported by weak evidence

BS	surgery vs. no surgery	Overall CA inc	4	1,745 / 31,807	0.75 (0.64-0.88)	0.40 (0.22-0.71)	1.9E-3	0.03-6.14	0.69 (0.49-0.97)	0.03	94	4/3.29	1.00
BMI	per 5kg/m^2 increase	Oesophageal AdCa inc, men	5	996 / 3,196,747	1.67 (1.44-1.92)	1.52 (1.33-1.74)	1.3E-9	1.09-2.11	1.31 (1.04-1.64)	0.37	24	4/3.6	1.00
BMI	per 5kg/m^2 increase	Oesophageal AdCa inc, women	3	922 / 3234247	1.54 (1.26-1.89)	1.51 (1.30-1.75)	2.8E-8	0.59-3.86	1.49 (1.05-2.12)	0.44	0	2/2.7	NP
CeAd##	high vs. low	Oesophageal AdCa inc	5	614 / 568285	1.81 (1.24-2.64)	2.51 (1.56-4.04)	1.5E-4	0.55-11.4	1.96 (1.18-3.26)	0.66	62	4/3.8	1.00
BMI	30+ vs. < 25	Gastric CA inc	14	56,508 / 5,158,082	1.23 (1.02-1.49)	1.13 (1.03-1.24)	8.8E-3	0.98-1.31	1.10 (0.99-1.22)	0.52	8	2/9.50	NP
BMI	30+ vs. < 25	Colon CA inc, women	14	19,749 / 1,754,026	1.07 (1.02-1.12)	1.22 (1.07-1.40)	3.4E-3	0.85-1.75	1.09 (1.01-1.19)	0.04	51	6/2.2	<0.01
BS	surgery vs. no surgery	Colorectal CA inc	5	1,013 / 182,298	0.79 (0.57-1.10)	0.77 (0.62-0.96)	0.022	0.54-1.10	0.78 (0.61-0.99)	0.06	0	0/1.97	NP
BMI	25-29.99 vs. < 25	Liver CA inc	14	16,382 / 3,976,906	1.07 (1.00-1.15)	1.18 (1.06-1.31)	1.7E-3	0.88-1.58	1.07 (1.01-1.14)	0.08	61	4/2.57	0.31
BMI	per 5kg/m^2 increase	Lung CA inc, women	6	5,388 / 1,447,141	0.86 (0.82-0.90)	0.80 (0.66-0.98)	0.027	0.42-1.53	0.92 (0.80-1.06)	0.66	85	3/2.96	1.00
BMI	25-29.99 vs. < 25	Melanoma inc	31	12,109 / 5,319,318	1.05 (0.95-1.16)	1.13 (1.02-1.26)	0.022	0.82-1.55	1.03 (0.97-1.10)	0.02	41	4/2.37	0.29
BMI	25+ vs. <25, ASE	Melanoma inc	18	5,308 / 950,355	0.99 (0.65-1.50)	1.24 (1.01-1.52)	0.045	0.63-2.45	1.12 (0.94-1.32)	0.03	49	4/0.91	0.01
BMI	per 5kg/m^2 increase	Melanoma inc, men	6	6,647 / 2,808,095	1.16 (1.08-1.26)	1.17 (1.05-1.30)	5.0E-3	0.89-1.52	1.11 (0.96-1.27)	0.89	46	3/2.46	0.69
BMI	per 5kg/m^2 increase	Melanoma inc, women	5	7,148 / 2,886,890	0.97 (0.91-1.00)	0.96 (0.93-1.00)	0.03	0.90-1.02	0.97 (0.93-1.01)	0.28	0	1/0.49	0.40
Obe±±	highest vs. lowest cat.**	Breast CA inc, PoMP, ER- PR+	4	5,760 / 96,656	2.20 (0.88-5.49)	2.01 (1.22-3.31)	6.5E-3	0.67-6.04	1.80 (0.98-3.28)	0.69	0	1/4.00	NP
Obe±±	highest vs. lowest cat.**	Breast CA inc, PrMP, ER+ PR+	6	8,678 / 17,365	0.77 (0.62-0.96)	0.80 (0.70-0.92)	1.4E-3	0.66-0.97	0.84 (0.70-1.00)	0.78	0	1/5.29	5.29
WHR	highest vs lowest cat.	Breast CA inc, PoMP	12	6,350 / 97,498	1.22 (0.96-1.55)	1.46 (1.15-1.85)	2.1E-3	0.69-3.07	1.15 (1.00-1.33)	0.05	65	4/5.11	NP
WHR	highest vs lowest cat.	Breast CA inc, PrMP	11	3,522 / 62,839	1.70 (1.30-2.30)	1.76 (1.25-1.47)	1.5E-3	0.55-5.60	1.27 (0.99-1.64)	0.03	78	8/9.23	NP
BMI	Obese vs. normal**	Breast CA inc, triple negative	5	933 / 158,657	1.24 (0.98-1.57)	1.24 (1.06-1.46)	8.2E-3	0.96-1.61	1.24 (1.03-1.49)	0.89	0	0/1.7	NP
BMI	30+ vs. < 25	Cervical CA inc	9	2,557 / 5,369,546	1.21 (1.06-1.37)	1.45 (1.15-1.83)	1.1E-3	0.78-2.72	1.23 (1.04-1.46)	0.17	57	4/2.66	0.46
BS	surgery vs. no surgery	Endometrial CA inc	3	12,288 / 958,988	0.48 (0.43-0.55)	0.39 (0.19-0.79)	8.6E-3	0.0-1314	0.55 (0.25-1.20)	0.74	80	2/2.48	NP
HC	per 10 cm increase	Endometrial CA inc	2	831 / 255650	1.32 (1.20-1.45)	1.29 (1.19-1.41)	2.9E-9	NA	1.23 (0.98-1.54)	NA	0	1/1.7	NP
BMI	per 5kg/m^2 increase	Endometrial CA inc: HRT-	3	699 / 360,326	1.61 (1.41-1.85)	1.90 (1.56-2.30)	9.2E-11	0.19-18.6	1.80 (1.06-3.07)	0.005	77	3/2.9	1.00
BMI	per 5kg/m^2 increase	Endometrial CA inc: HRT+	6	791 / 679,271	1.25 (1.05-1.47)	1.18 (1.07-1.31)	1.7E-3	1.02-1.37	1.16 (1.02-1.31)	0.77	0	1/1.7	NP
WG	per 5kg increase	Endometrial CA inc: PoMP, HRT-	2	285 / 33,340	1.36 (1.25-1.46)	1.38 (1.28-1.49)	3.6E-17	NA	1.40 (0.97-2.02)	NA	0	2/1.0	0.50
WG	per 5kg increase	Endometrial CA inc: PoMP, HRT+	2	334 / 35,333	1.09 (1.00-1.18)	1.09 (1.02-1.17)	0.01	NA	1.09 (0.99-1.20)	NA	0	1/0.2	0.18
BMI	per 5kg/m^2 increase	Endometrial CA mort	3	962 / 1781648	1.42 (1.33-1.63)	1.46 (1.29-1.65)	1.7E-9	0.48-4.48	1.35 (0.90-2.05)	0.64	29	2/2	1.00
WG	per 5kg increase	Ovarian CA inc: PostMP, HRT-	2	217 / 23,984	1.16 (1.03-1.31)	1.13 (1.03-1.24)	8.5E-3	NA	1.11 (0.99-1.25)	NA	0	0.27	0.25
BMI	30+ vs. < 25	Ovarian CA inc, PoMP	5	1,116 / 1,858,520 pyrs	1.02 (0.82-1.26)	1.23 (1.03-1.47)	0.03	0.72-2.09	1.12 (0.97-1.29)	0.52	46	0.3	0.14
BMI	30+ vs. < 25	Ovarian CA inc, PrMP	3	284 / 1,758,718 pyrs	1.56 (1.14-2.16)	1.57 (1.20-2.06)	9.7E-4	0.27-9.02	1.54 (0.95-2.51)	0.61	0	1.7	NP
Weight	per 5kg increase	Ovarian CA inc	4	1,281 / 405,655	1.02 (1.00-1.05)	1.03 (1.01-1.05)	1.4E-3	0.98-1.08	1.03 (1.00-1.05)	0.42	7	1/0.22	0.20

BMI	per 5kg/m^2 increase	Prostate CA inc, advanced	13	7,067 / 1,080,790	1.00 (0.94-1.08)	1.09 (1.02-1.16)	0.01	0.92-1.29	1.04 (0.99-1.09)	0.02	40	3/0.65	0.02
WG	per 5kg increase	Prostate CA inc, high screening rate	2	4,894 / 67,335	0.96 (0.92-1.00)	0.95 (0.92-0.99)	7.0E-3	NA	0.95 (0.90-1.01)	NA	0	1/0.4	0.25
WG	highest vs. lowest cat.	Kidney CA inc	3	1,780 / 781,293	1.20 (0.90-1.70)	1.42 (1.11-1.81)	4.7E-3	0.23-8.71	1.28 (0.97-1.69)	0.89	10	1/2.02	NP
BMI	BMI 25-29.99 vs. < 25	Bladder CA inc	26	28,745 / 12,562,510	1.05 (1.00-1.10)	1.07 (1.01-1.14)	0.03	0.90-1.27	1.04 (0.99-1.08)	0.29	38	5/3.72	0.41
BMI	per 5kg/m^2 increase	Thyroid CA inc, men	4	3,526 / 3,157,142	1.09 (0.98-1.22)	1.32 (1.04-1.69)	0.025	0.46-3.84	1.12 (1.00-1.25)	0.23	77	2/1.02	0.27
BMI	30+ vs. < 25	Hodgkin's lymphoma inc	4	2,716 / 4,828,366	1.66 (1.28-2.14)	1.41 (1.14-1.75)	1.9E-3	0.73-2.72	1.24 (0.96-1.59)	0.74	22	1/3.54	NP
BMI	25-29.99 vs. < 25	NHL inc	23	35,955 / 7,644,651	1.03 (0.97-1.10)	1.07 (1.01-1.14)	0.024	0.87-1.33	1.03 (1.00-1.07)	0.40	57	4/2.15	0.27
BMI	30+ vs. < 25	NHL inc	23	35,955 / 7,644,651	1.09 (1.00-1.19)	1.20 (1.07-1.34)	1.4E-3	0.78-1.84	1.05 (0.97-1.15)	0.50	72	9/6.87	0.36
BMI	per 5kg/m^2 increase	NHL mort	7	5,920 / 3,511,893	1.18 (1.09-1.29)	1.14 (1.04-1.25)	5.3E-3	0.90-1.45	1.10 (1.01-1.20)	0.57	53	3/3.37	NP
BMI	25-29.99 vs. < 25	Diff large B-cell lymphoma inc	19	16,766 / 2,437,709	1.26 (1.08-1.47)	1.14 (1.04-1.24)	3.6E-3	0.89-1.45	1.10 (1.01-1.20)	0.08	33	6/11.8	NP
BMI	30+ vs. < 25	Multiple myeloma inc	19	15,887 / 7,729,520	1.31 (1.16-1.48)	1.21 (1.08-1.35)	1.0E-3	0.90-1.62	1.14 (1.01-1.28)	0.56	34	5/11.2	NP
BMI	25-29.99 vs. < 25	Multiple myeloma mort	8	3,337 / 3,714,187	1.18 (1.01-1.39)	1.15 (1.04-1.26)	6.0E-3	1.02-1.30	1.12 (0.99-1.27)	0.46	0	1/2.81	NP
BMI	per 5kg/m^2 increase	Leukemia inc, men	5	7,827 / 3,497,392	1.09 (1.02-1.17)	1.08 (1.02-1.13)	8.4E-3	0.99-1.17	1.06 (0.98-1.14)	0.47	0	1/1.54	NP
BMI	per 5kg/m^2 increase	Leukemia inc, women	3	7,290 / 3,260,868	1.07 (1.02-1.12)	1.17 (1.04-1.33)	9.8E-3	0.28-4.93	1.10 (1.00-1.21)	0.20	79	3/1.09	0.05
BMI	30+ vs. < 25	Leukemia inc, ALL	5	588 / 13,337,737	1.33 (0.82-2.15)	1.65 (1.16-2.35)	5.1E-3	0.85-3.23	1.40 (0.96-2.04)	0.67	7	1/1.9	NP
BMI	30+ vs. < 25	Leukemia inc, CML	5	2,530 / 13,337,737	1.15 (0.92-1.45)	1.26 (1.09-1.46)	1.7E-3	1.00-1.60	1.20 (1.03-1.41)	0.47	0	1/1.9	NP

Abbreviations: AdCa, adenocarcinoma; ALL, acute lymphatic leukemia; AML, acutel myeloic leukemia; ASE, adjusted for sunlight exposure; biliary tract system cancer, includes cancers of gallbladder, extrahepatic bile duct and ampulla of Vateri; BMI, Body Mass Index; BMI iya, Body Mass Index in young adulthood; BS, bariatric surgery; CA, cancer; CI, confidence interval; CLL, chronic lymphatic leukemia; CML, chronic myeloic leukemia; ER = estrogen receptor status; HC, hip circumference; HRT, hormone replacement therapy; inc, incidence; mort, mortality; NA: Not available, due to <3 included studies; NHL, Non-Hodgkin lymphoma; NP: Not Pertinent, because the estimated is larger than the observed, and there is no evidence of excess statistical significance based on the assumption made for the plausible effect size; PoMP, postmenopausal; PR = progesterone receptor status; PrMP, premenopausal; pyrs, person-years; RR, relative risk; SqCa, squamous cell carcinoma; WC, waist circumference; WG, weight gain; WHR, waist-to-hip ratio;

* Number of studies
± Sum of all study cohorts in the meta-analysis. In case the same study appears more than once (e.g. for both men and wom, and proportions of men and wom in the study cohort was not given), the sum given here may too large.
# Relative risk and 95% confidence interval of largest study (smallest SE) in each meta-analysis.
† Random effects refer to summary risk ratio (95% CI) using the random-effects model.
¶ P value of summary random effects estimate.
‡ P-value from the Egger's regression asymmetry test.
§ Expected number of statistically significant studies using the point estimate of the largest study (smallest standard error) as the plausible effect size.
** Observed/Expected number of statistically significant studies
±± BMI, weight gain or weight change, varies between included studies
|| P value of the excess statistical significance test.
Central adiposity: dependent categorical comparison of visceral adipose tissue area. if that was unavailable, WHR (or waist thigh ratio) or last WC was used.
*** Only associations meeting at least weak evidence are included in the table

All statistical tests were two-sided.

Search Algorithms used

1) Pubmed:

1: obesity OR obese OR BMI OR “body mass index” OR bariatric surgery OR hip circumference OR waist circumference OR “weight gain” OR “waist to hip ratio” OR weight OR height OR body fat*
2: cancer OR carcinoma OR neoplasia OR tumor OR neoplasm OR maligna*
3: meta-analysis OR systematic review
4: 1 AND 2 AND 3

2) EMBASE:

1: obesity/ OR obese/ OR BMI/ OR body mass index/ OR bariatric surgery/ OR hip circumference/ OR waist circumference/ OR weight gain/ OR waist to hip ratio/ OR weight/ OR height/ OR body fat/
2: cancer/ OR carcinoma/ OR neoplasia/ OR tumor/ OR neoplasm/ OR maligna/
3: meta-analysis OR systematic review
4: 1 AND 2 AND 3

3) Cochrane database of systematic reviews:

1: obesity or obese or BMI or “body mass index” or bariatric surgery or hip circumference or waist circumference or “weight gain” or "waist to hip ratio" or weight or height or body fat:ti,ab,kw
2: cancer or carcinoma or neoplasia or tumor or neoplasm or maligna:ti,ab,kw
3: #1 and #2; in Cochrane Reviews (Reviews only) and Other Reviews
